# Acupoint stimulation methods for premature ovarian insufficiency: a systematic review and network meta-analysis of randomized controlled trials

**DOI:** 10.3389/fendo.2025.1604563

**Published:** 2025-07-18

**Authors:** Zhenping Du, Guancheng Ye, Jingyuan Wei, Shutong Li, Shan Zhao, Jun Wang

**Affiliations:** ^1^ Department of Acupuncture, Dongzhimen Hospital, Beijing University of Chinese Medicine, Beijing, China; ^2^ Department of Rheumatology, Dongzhimen Hospital, Beijing University of Chinese Medicine, Beijing, China

**Keywords:** premature ovarian insufficiency, POI, acupoint stimulation methods, network meta-analysis, NMA, systematic review

## Abstract

**Objective:**

The aims of this study were to evaluate and rank the efficacy of different acupoint stimulation (AS) therapies in the treatment of premature ovarian insufficiency (POI) by network meta-analysis (NMA) and to explore the optimal AS regimen for improving sex hormone levels and clinical symptoms.

**Methods:**

Randomized controlled trials (RCTs) in English and Chinese up to November 2024 were searched in eight databases, including the Cochrane Library, PubMed, and the China National Knowledge Infrastructure (CNKI). Included studies were patients with POI diagnosed according to established international or Chinese guidelines. The intervention compared AS therapies (e.g., acupuncture, moxibustion, and electroacupuncture) with conventional treatments (oral herbs or hormone replacement therapy) or/and placebo. Outcome indicators included antral follicle count (AFC), follicle-stimulating hormone (FSH) levels, luteinizing hormone (LH) levels, estradiol (E_2_) levels, and Kupperman scores. The outcome indicators were evaluated on days 2–5 of the menstrual period. Exclusion criteria included non-RCTs, duplicate publications, non-peer-reviewed literature, studies with unclear outcome/baseline data, studies with a non-POI diagnosis, studies with a before-and-after own-control design, non-human studies, and studies with missing continuous data. Literature quality and risk of bias were assessed using Review Manager (RevMan), Version 5.3. Statistical analysis was performed using Stata 16.0 software, mvmeta package, and surface under the cumulative ranking curve (SUCRA).

**Results:**

A total of 51 RCTs were retrieved, including 3,754 patients. A methodological assessment of 51 RCTs revealed generalized risks: 9.8% of RCTs used incorrect randomization methods; 29% of RCTs did not describe explicit randomization; all RCTs did not have explicit allocation concealment; 98% of RCTs did not mention blinding; all RCTs reported complete outcome data; and 98% of RCTs had unclear risks. Therapies combining multiple AS therapies (especially moxibustion-related therapies) improved ovarian function significantly. Acupuncture combined with moxibustion (Acu + Moxi) was effective in increasing AFC [mean difference (MD) = 2.04, 95% confidence interval (CI) (1.31, 2.77); SUCRA = 75.1%]. Auricular seed therapy (AST) was most effective in reducing FSH [MD = 3.03, 95% CI (0.45, 5.60); SUCRA = 83.7%]. Acupuncture (Acu) was effective in reducing LH levels [MD = 1.52, 95% CI (0.27, 2.77); SUCRA = 58.3%]. Moxibustion combined with tuina (Moxi + MT) [MD = 11.92, 95% CI (8.19, 15.65); SUCRA = 100%] and moxibustion combined with acupressure [MD = 3.20, 95% CI (0.16, 6.24); SUCRA = 81.4%] significantly increased E_2_ levels. Moxibustion combined with tuina (Moxi + MT) had the best effect on Kupperman score improvement [MD = 4.63, 95% CI (0.58, 8.68); SUCRA = 92.1%]. Adverse events for all therapies involved in this study were mild and resolved with symptomatic management.

**Conclusion:**

AS therapies (especially those combined with moxibustion) can safely and effectively improve hormone levels and clinical symptoms in patients with POI, supporting their clinical application as complementary and alternative medicine (CAM).

**Systematic Review Registration:**

https://www.crd.york.ac.uk/PROSPERO/, identifier CRD42024612169.

## 1 Introduction

Premature ovarian insufficiency (POI), previously known as premature ovarian failure (POF) (1), is a clinical syndrome characterized by the cessation of ovarian function before the age of 40. It is clinically defined as the absence of menstruation for more than 4 months in women under 40, accompanied by elevated serum follicle-stimulating hormone (FSH) levels (serum FSH >25 IU/L in two consecutive menstrual cycles) (2). According to the latest statistical data, the global incidence rate of POI is 3.7% (95% confidence interval: 3.1% to 4.3%) (3). POI impacts women’s overall health and fertility. If left untreated, it can lead to a decrease in bone density and thus an increased risk of osteoporosis, as well as an increased risk of cardiovascular disease, and is also associated with possible cognitive dysfunction in women with POI who undergo surgery. Furthermore, POI is linked to higher overall mortality rates (2). Notably, the psychosocial burden caused by POI is particularly significant, with patients exhibiting a three to four times higher prevalence of major depressive disorder compared to healthy women of the same age (4). This underscores the multidimensional health burden of POI and the urgent need for effective interventions.

Ovarian function depends on precisely regulated developmental processes initiated during embryogenesis. Following female sex determination, coordinated molecular signaling directs ovarian differentiation and the establishment of the oocyte pool. Disruptions in this cascade may cause ovarian dysgenesis (“streak gonads”), representing the severe end of the POI spectrum. The ovaries’ primary role—maintaining oocyte integrity through decades of dormancy—requires robust protection against oxidative stress and DNA damage. Compromise of these protective mechanisms and dysregulated folliculogenesis contribute to POI pathogenesis (2). The main causes of POI are genetic abnormalities, autoimmune diseases, mitochondrial dysfunction, medical factors (e.g., chemotherapy, radiotherapy, and surgery), and environmental factors (e.g., viral infections and toxins) (5, 6). Currently, prevention and treatment options for POI remain severely limited. Traditional hormone replacement therapy (HRT) has proven ineffective in restoring ovarian function (7). Novel approaches, such as *in vitro* activation, mitochondrial activation, stem cell and exosome therapies, biomaterial strategies, and intraovarian infusion of platelet-rich plasma, remain in experimental stages and are undergoing rigorous scientific scrutiny to establish their efficacy and safety before being accepted as viable clinical options (8).

Acupoint stimulation (AS) therapy is an important component of complementary and alternative medicine (CAM) and has shown promising efficacy in the management of POI (9). TCM’s AS therapy stimulates specific acupoints in the human body through physical or chemical methods to regulate the body’s qi, blood, meridians, and internal organs, thereby preventing and treating diseases and promoting health. Stimulation methods include acupuncture, moxibustion, catgut implantation at acupoint (CIAA), electroacupuncture (EA), auricular seed therapy (AST), and other therapies. Extensive preclinical studies have demonstrated that AS’s mechanism of action in the treatment of POI includes unblocking the meridians, promoting blood circulation, regulating the hypothalamic–pituitary–ovarian (HPO) axis, improving hormone levels, improving the generation of the anti-apoptotic factor Bcl-2 in the ovarian granulosa cells, and decreasing the production of the pro-apoptotic factor Bax (10). AS demonstrates distinct advantages in mitigating the clinical symptoms of POI, regulating sex hormone levels, revitalizing residual follicles, and restoring menstrual cycles (11, 12).

In recent years, there has been an increase in the number and size of randomized controlled trials (RCTs) focusing on POIs, and several systematic reviews have confirmed the efficacy and safety of AS (13–15). However, most of the original studies focused only on the efficacy of a single AS therapy or a combination of two AS therapies for POI, and systematic reviews only summarized the cumulative effect of all AS treatments rather than the differences between various AS therapies. They did not evaluate the outcomes of all existing AS therapies included in the systematic reviews, such as manual acupuncture, moxibustion, EA, acupoint catgut embedding, and auricular acupuncture. Therefore, there is a pressing need to identify the most effective AS therapy for treating POI. Bayesian network meta-analysis (NMA) provides a robust framework for comparisons between multiple therapies by integrating prior distributions and probabilistic rankings (16). Using NMA, we compared and ranked the efficacy of all existing AS therapies used to treat POI. Our findings provide robust evidence to support the use of AS and provide guidance for the management of POI and the development of health policies.

## 2 Materials and methods

This meta-analysis was conducted following the Preferred Reporting Items for Systematic Reviews and Meta-Analyses (PRISMA) guidelines (**Supplementary Table S1**) and has been registered on the International Prospective Register of Systematic Reviews (PROSPERO) (registration number: CRD42024612169).

### 2.1 Search strategy

To retrieve relevant articles, we searched databases, including the Cochrane Library, Web of Science, PubMed, Embase, Chinese Scientific and Technological Journal Database (VIP), China National Knowledge Infrastructure (CNKI), Wanfang Database (WF), and China Biomedical Literature Database (CBM), from their inception to 28 November 2024. The search strategy is the Medical Subject Headings (MeSH) and free text terms, with the objective of published studies. **Table 1** demonstrates the search strategies applied when searching the PubMed database. **Supplementary Table S2** shows the search strategy for all databases in detail.

**Table 1:** Search strategy (through PubMed).

ID	Search terms	Results
#1	(“Primary Ovarian Insufficiency”[Mesh]) OR (premature ovarian failure [Title/Abstract]	5,310
#2	needle*[Title/Abstract] OR acupuncture point*[Title/Abstract] OR acupoint*[Title/Abstract] OR ‘Point, Acupuncture’[Title/Abstract]	157,561
#3	“Acupuncture”[Mesh] OR “Acupuncture Therapy”[Mesh] OR “Acupuncture, Ear”[Mesh] OR “Acupuncture Points”[Mesh] OR “Electroacupuncture”[Mesh] OR “Moxibustion”[Mesh] OR “Massage”[Mesh])	38,801
#4	#2 OR #3	185,553
#5	#1 AND #4	54

### 2.2 Inclusion and exclusion criteria

#### 2.2.1 Type of studies

Publicly available RCTs were language-limited to Chinese or English.

#### 2.2.2 Type of participants

Eligible study subjects were patients with a clinical diagnosis of POI based on the diagnostic criteria for POI as proposed in the international published guidelines for the management of POI (1, 3), *Chinese Obstetrics and Gynecology*, or the ninth edition of *Obstetrics and Gynecology* (17, 18).

#### 2.2.3 Type of interventions

The observation group used at least one AS therapy or a combination of two or more AS therapies. The control group was given conventional drug therapy (oral herbal or HRT) or/and placebo. There were no restrictions on treatment sites and duration, needles, materials, drug formulations, or dosages for the two groups.

#### 2.2.4 Type of outcome indicator

All studies are required to include any of the outcome indicators listed as follows: (a) antral follicle count (AFC) to assess ovarian reserve function; (b) FSH and luteinizing hormone (LH) to assess ovarian function and endocrine status; (c) estradiol (E_2_) used to reflect ovarian reserve function and follicular development; and (d) Kupperman scores, which were used to evaluate the severity of symptoms similar to the perimenopausal syndrome in patients with POI. The outcome indicators were evaluated on days 2–5 of the menstrual period.

#### 2.2.5 Exclusion criteria

The following criteria were used to exclude studies: (a) studies that are not RCTs; (b) instances of repeated publication of the same study; (c) non-periodical literature, including conference papers, abstracts, and dissertations; (d) studies lacking clear outcome measures or baseline data; (e) studies without a clear POI diagnosis or those involving the integration of other diseases; (f) pre–post studies within the same patient group; (g) non-human testing (including animal testing and cellular testing); and (h) studies where data are not presented in terms of mean and standard deviations.

### 2.3 Data extraction and quality evaluation

To ensure the accuracy of the data, two researchers (Du and Ye) checked and verified with each other after independently extracting the data. In the event of inconsistent results, discrepancies were resolved either through discussion or, when necessary, by consulting an independent third party (Wei) to reach a consensus. The extracted data included the following: (a) basic information; (b) baseline characteristics of participants and the treatment methods applied; (c) key elements to assess the risk of bias; and (d) outcome measurements and associated data.

### 2.4 Risk of bias of the included studies

Two researchers (Du and Ye) independently assessed the risk of bias of the included RCTs and cross-checked their evaluations to ensure consistency. In case of disagreement, a third researcher (Wei) was consulted for reassessment. The risk of bias assessment of the included studies was conducted using the tool recommended in the *Cochrane Handbook for Systematic Reviews* (Version 5.1.0).

### 2.5 Statistical analysis

This study employed the mvmeta package in Stata 16.0 and Stata 12.0 software for network and routine meta-analyses, using mean ± standard deviation as effect size metrics for quantitative data analysis. All effect indicators were presented with mean difference (MD) and 95% confidence intervals (CIs). Literature quality and risk of bias were evaluated via Review Manager (RevMan), Version 5.3. Clinical and methodological features were compared to evaluate evidence network similarity, with outcome comparisons between any two interventions presented in league tables. A significance level of α = 0.05 was adopted. Heterogeneity was quantified using the *I*² statistic, with values exceeding 50% indicating substantial heterogeneity. Fixed-effects models were used when *p* ≥ 0.05 and *I*² ≤ 50%; otherwise, random-effects models were applied. When significant heterogeneity was detected, effect sizes were aggregated using a random-effects model, and subgroup analyses were performed for all studies according to different interventions, with sensitivity analyses performed using a dropout method for subgroups with inhibitions. The robustness of the results was assessed by systematically excluding high-risk studies or subgroups exhibiting severe heterogeneity on a leave-one-out method. Surface under the cumulative ranking curve (SUCRA) values (0%–100%) probabilistically ranked intervention efficacy, with higher values indicating greater likelihood of being the optimal treatment (19, 20).

## 3 Results

### 3.1 Included articles

**Figure 1:**
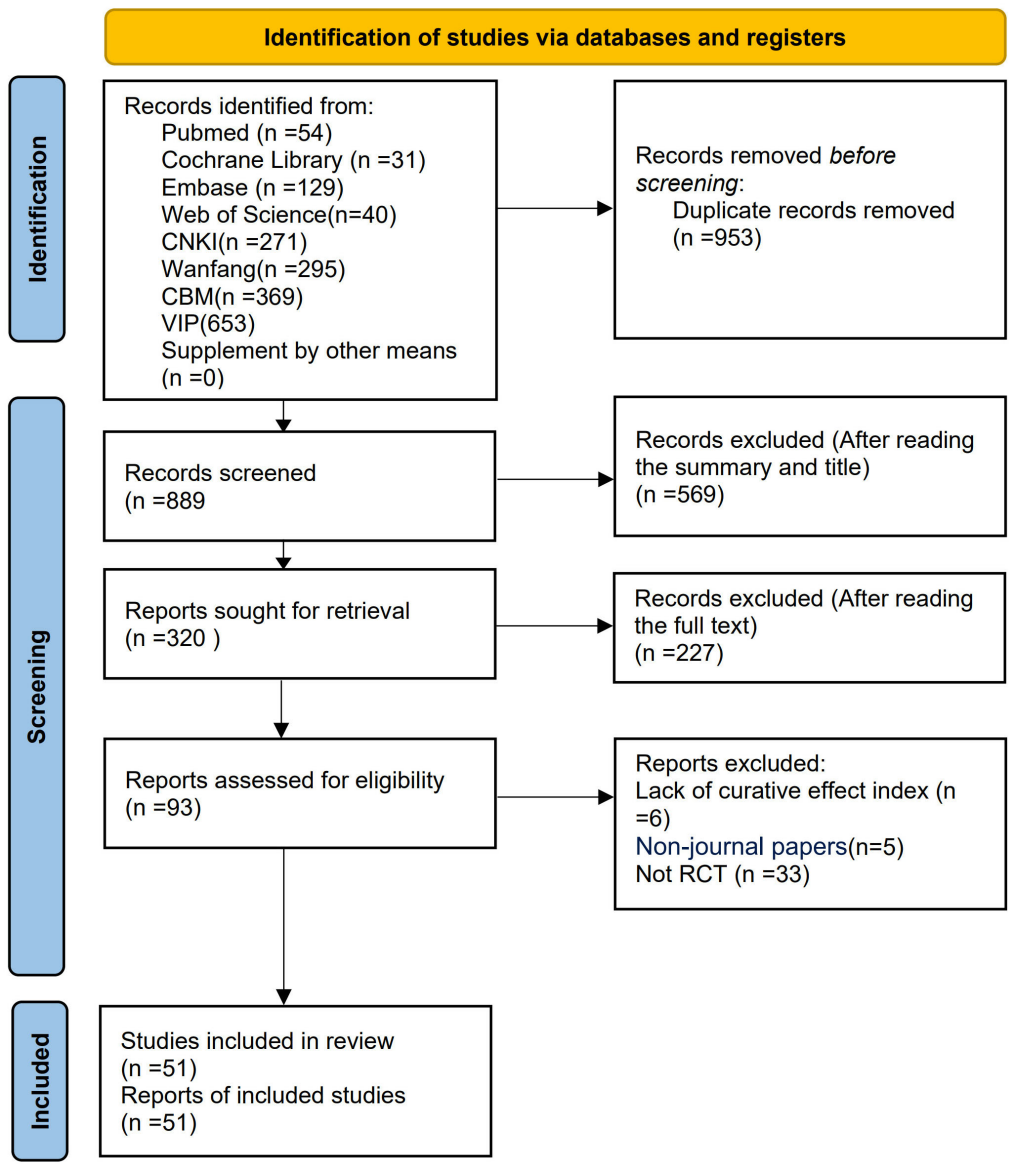
Literature screening process and results.

The preliminary search yielded a total of 1,842 relevant articles across multiple databases, including the Cochrane Library (*n* = 31), Web of Science (*n* = 40), PubMed (*n* = 54), Embase (*n* = 129), VIP (*n* = 653), CNKI (*n* = 271), WF (*n* = 295), and CBM (*n* = 369). After multiple rounds of rigorous screening, 51 studies (21–71) were ultimately included in this meta-analysis. The publication dates of these studies spanned from 1999 to 2024. The detailed literature screening flowchart and results are presented in **Figure 1**.

### 3.2 Study characteristics

**Figure 2:**
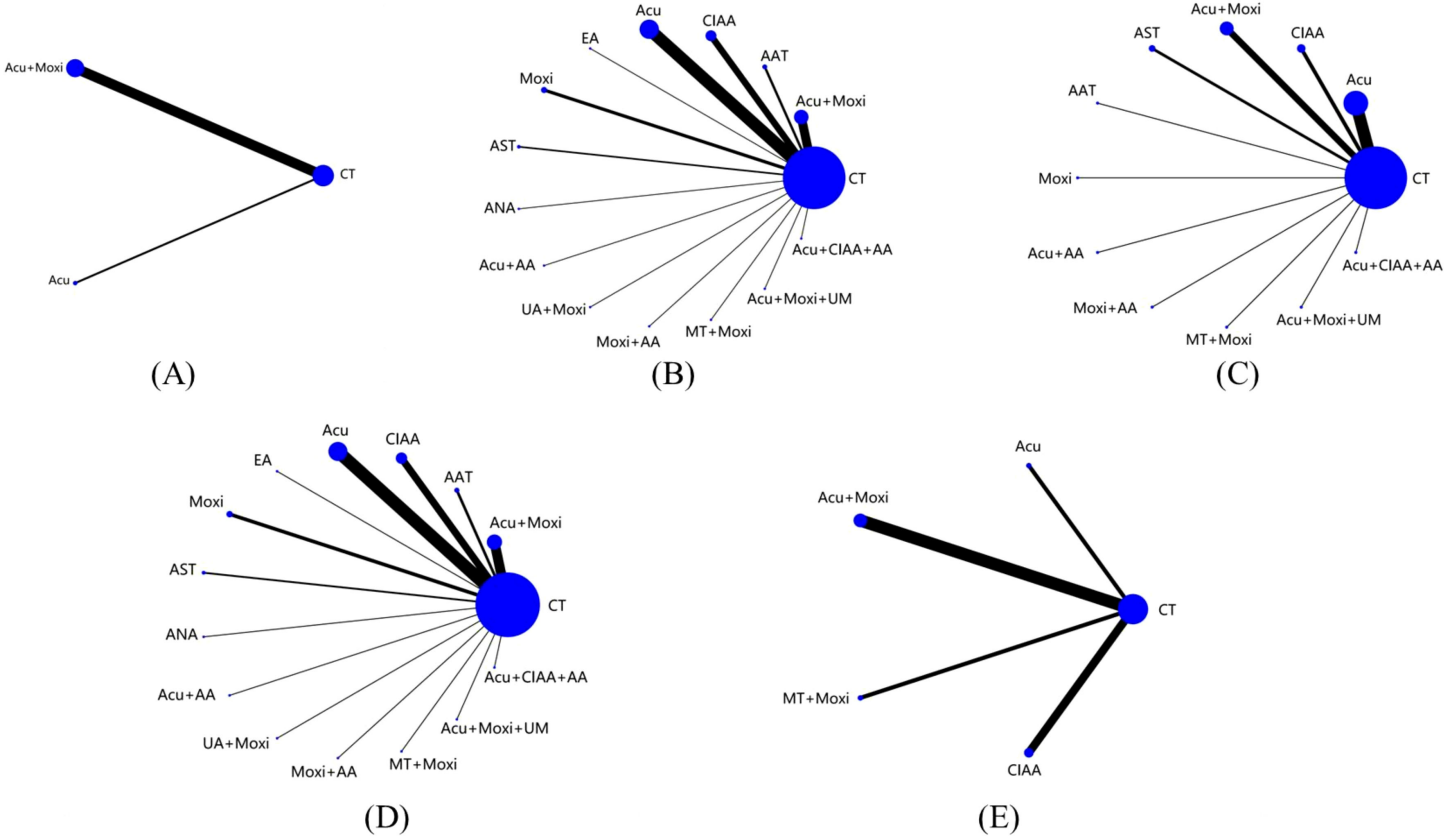
Evidence network of all studies on different outcome indicators. **(A)** Antral follicle count (AFC); **(B)** follicle-stimulating hormone (FSH); **(C)** luteinizing hormone (LH); **(D)** estradiol (E_2_); **(E)** Kupperman score. Line thickness indicates study count; node size shows sample size per treatment. CT, conventional therapy; Acu, acupuncture; Moxi, moxibustion; AAT, abdominal acupuncture therapy; CIAA, catgut implantation at acupoint; EA, electroacupuncture; AST, auricular seed therapy; ANA, awn needle acupuncture; UA, umbilical acupuncture; AA, acupoint application; MT, massage therapy; UM, umbilical moxibustion.

This NMA included a total of 51 studies involving 3,754 patients, all of which were RCTs (21–71). Twelve various methods of treatment were included in these studies: conventional treatment (CT), acupuncture (Acu), moxibustion (Moxi), abdominal acupuncture therapy (AAT), CIAA, EA, AST, awn needle acupuncture (ANA), umbilical acupuncture (UA), massage therapy (MT), acupoint application (AA), and umbilical moxibustion (UM). (**Supplementary Table S3** details the abbreviations and definitions of different therapies and conventional treatment.) For investigations using a three-arm experimental design, subgroup merging was used during the data processing phase. Data analysis was then conducted using a two-arm trial design. **Supplementary Table S4** provides further details about the included RCTs. A comprehensive network diagram illustrating various comparisons across all outcomes is shown in **Figure 2**.

### 3.3 Literature quality evaluation

This study included a total of 51 RCTs, with their quality comprehensively evaluated. Regarding the method of random sequence generation, five studies (42, 44, 51, 59, 70) assigned patients based on order of registration, visit sequence, or outpatient or inpatient number, resulting in the possibility of selection bias, which was judged to be high risk. Conversely, 31 studies (21, 25–29, 33–37, 39, 43, 47, 48, 50, 53–56, 58, 60–62, 64, 66–68, 71) were considered low risk because they employed random number tables or computer-based random sequence generators for participant allocation. However, 15 studies (22–24, 30–32, 38, 40, 41, 45, 46, 49, 52, 57, 65) mentioned randomization but did not describe the method of randomization and were therefore classified as having unclear risks.

**Figure 3:**
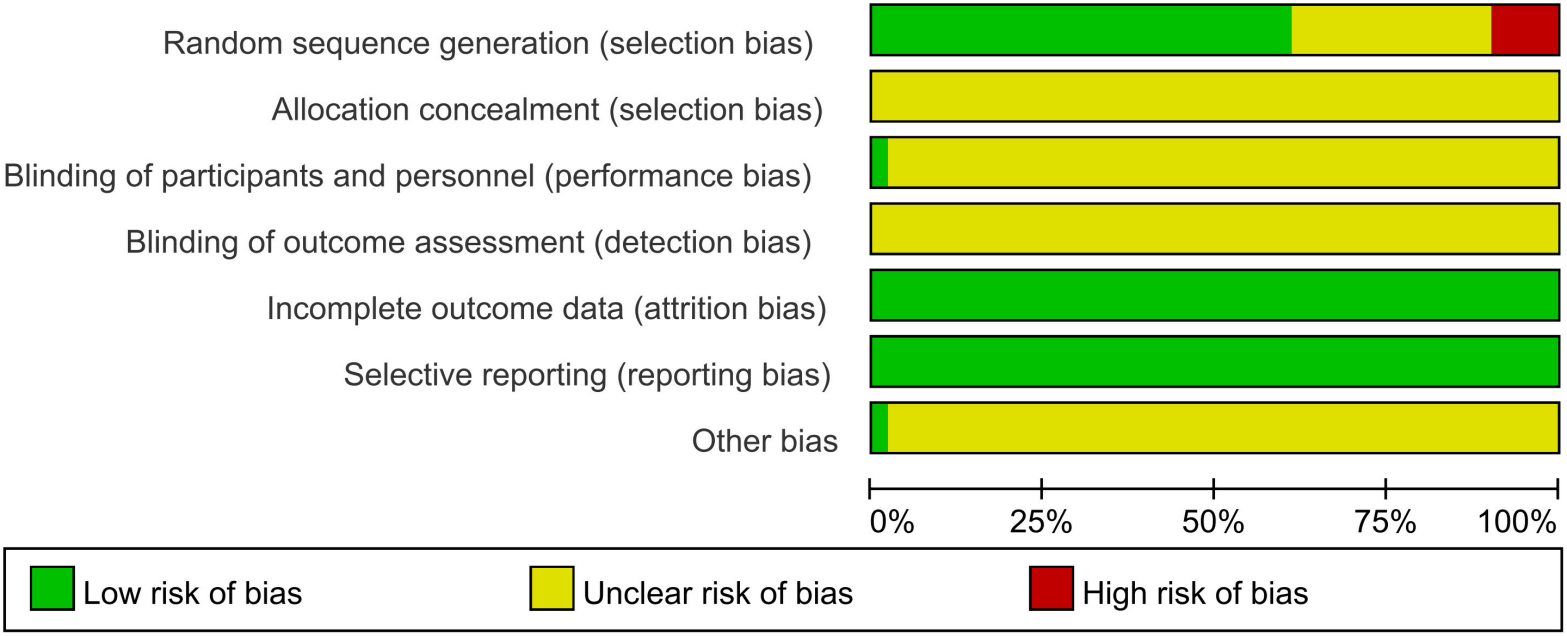
Risk of bias assessment.

Concerning allocation concealment, all studies (21–71) failed to clearly describe their allocation concealment methods, resulting in an unclear risk rating. Only one study (30) implemented blinding of participants and investigators, assessed as low risk, while the remaining studies (21–29, 31–71) did not mention blinding and were rated as unclear risk. All studies (21–71) maintained complete datasets. The presence of selective reporting bias was determined by verifying that the methods were aligned with the presented outcomes. All studies (21–71) adequately reported their findings and were deemed low risk in this aspect. One study (58) demonstrated a rigorous design with no apparent sources of bias not covered by other domains, rated as low risk, while the remaining studies (21–57, 59–71) retained unclear risks regarding other potential bias sources. **Figure 3** illustrates the risk of bias assessment conducted with RevMan 5.3. Specific details of the evaluation are shown in **Supplementary Table S5**.

### 3.4 Outcome indicators

#### 3.4.1 Antral follicle count

**Figure 4:**
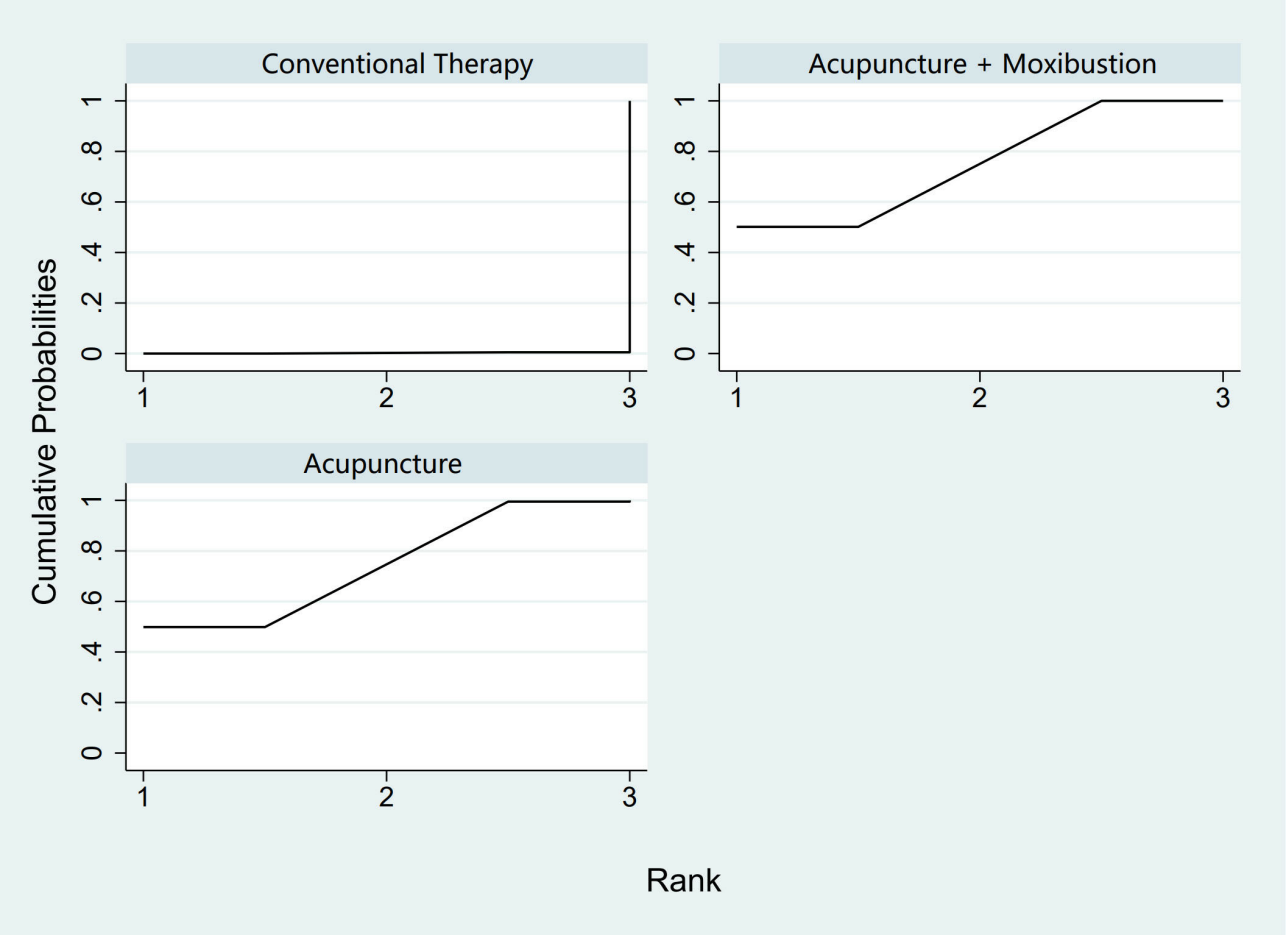
Efficacy ranking and cumulative probability graph of antral follicle count treated (AFC) by different types of intervention.

In the evaluation of AFC, six studies (23, 25, 34, 43, 54, 58) involving 401 patients were included. The NMA focused on two types of AS treatments and their effectiveness compared with conventional drug therapy or placebo. Compared with the control group, the treatments that showed significant improvements in AFC scores were as follows: Acu + Moxi therapy [MD = 2.04, 95% CI (1.31, 2.77)] and Acu therapy [MD = 2.02, 95% CI (0.45, 3.59)]. The pairwise comparison results are shown in **Supplementary Table S6.1**. The SUCRA analysis showed that Acu + Moxi had the highest probability of elevating AFC (75.1%), followed by Acu (74.7%). These results are presented in **Table 2** and **Figure 4**.

**Table 2:** The SUCRA values of each treatment modality.

Treatment	E_2_	Rank	FSH	Rank	LH	Rank	AFC	Rank	Kupperman score	Rank
CT	17.7	15	18.9	15	23	12	0.3	3	22	4
Acu + Moxi	61.6	3	53	6	49.8	6	75.1	1	45.3	3
AAT	59.7	4	42	11	48.8	7				
CIAA	52.9	6	47.3	7	64.6	2			73.8	2
Acu	53.6	5	63.3	4	58.3	5	74.7	2	16.8	5
EA	23.7	14	27.4	14						
Moxi	40	12	46.1	9	33.3	11				
AST	30.1	13	83.7	1	37.8	10				
ANA	50.6	7	40.5	13						
Acu + AA	41.9	11	41.6	12	41.1	9				
UA + Moxi	45.1	9	44.4	10						
Moxi + AA	81.4	2	69.8	2	77.1	1				
MT + Moxi	100	1	58.1	5	64	3			92.1	1
Acu + Moxi + UM	48.2	8	67.2	3	58.9	4				
Acu + CIAA + AA	43.5	10	46.6	8	43.4	8				

CT, conventional therapy; Acu, acupuncture; Moxi, moxibustion; AAT, abdominal acupuncture therapy; CIAA, catgut implantation at acupoint; EA, electroacupuncture; AST, auricular seed therapy; ANA, awn needle acupuncture; UA, umbilical acupuncture; AA, acupoint application; MT, massage therapy; UM, umbilical moxibustion; AFC, antral follicle count; FSH, follicle-stimulating hormone; LH, luteinizing hormone; E_2_, estradiol.

#### 3.4.2 Follicle-stimulating hormone levels

**Figure 5:**
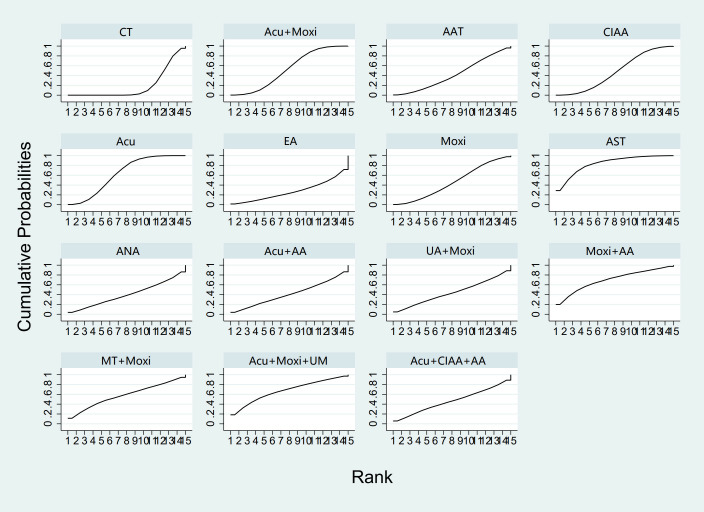
Efficacy ranking and cumulative probability graph of follicle-stimulating hormone (FSH) treated by different types of intervention. CT, conventional therapy; Acu, acupuncture; Moxi, moxibustion; AAT, abdominal acupuncture therapy; CIAA, catgut implantation at acupoint; EA, electroacupuncture; AST, auricular seed therapy; ANA, awn needle acupuncture; UA, umbilical acupuncture; AA, acupoint application; MT, massage therapy; UM, umbilical moxibustion.

In the evaluation of FSH levels, 51 studies (21–71) involving 3,720 participants were included. The meta-analysis assessed 14 types of AS treatments and their effectiveness. Compared with the control group, the therapies that demonstrated significant benefits in FSH were as follows: AST treatment [MD = 3.03, 95% CI (0.45, 5.60)], Acu therapy [MD = 1.64, 95% CI (0.72, 2.56)], and Acu + Moxi therapy [MD = 1.27, 95% CI (0.20, 2.35)]. The pairwise comparison results are shown in **Supplementary Table S6.2**. The SUCRA analysis showed that AST (83.7%) had the highest probability of decreasing FSH levels (83.7%), followed by Moxi + AA (69.8%), Acu + Moxi + UM (67.2%), Acu (63.3%), and Moxi + MT (58.1%). These results are presented in **Table 2** and **Figure 5**.

#### 3.4.3 Luteinizing hormone levels

**Figure 6:**
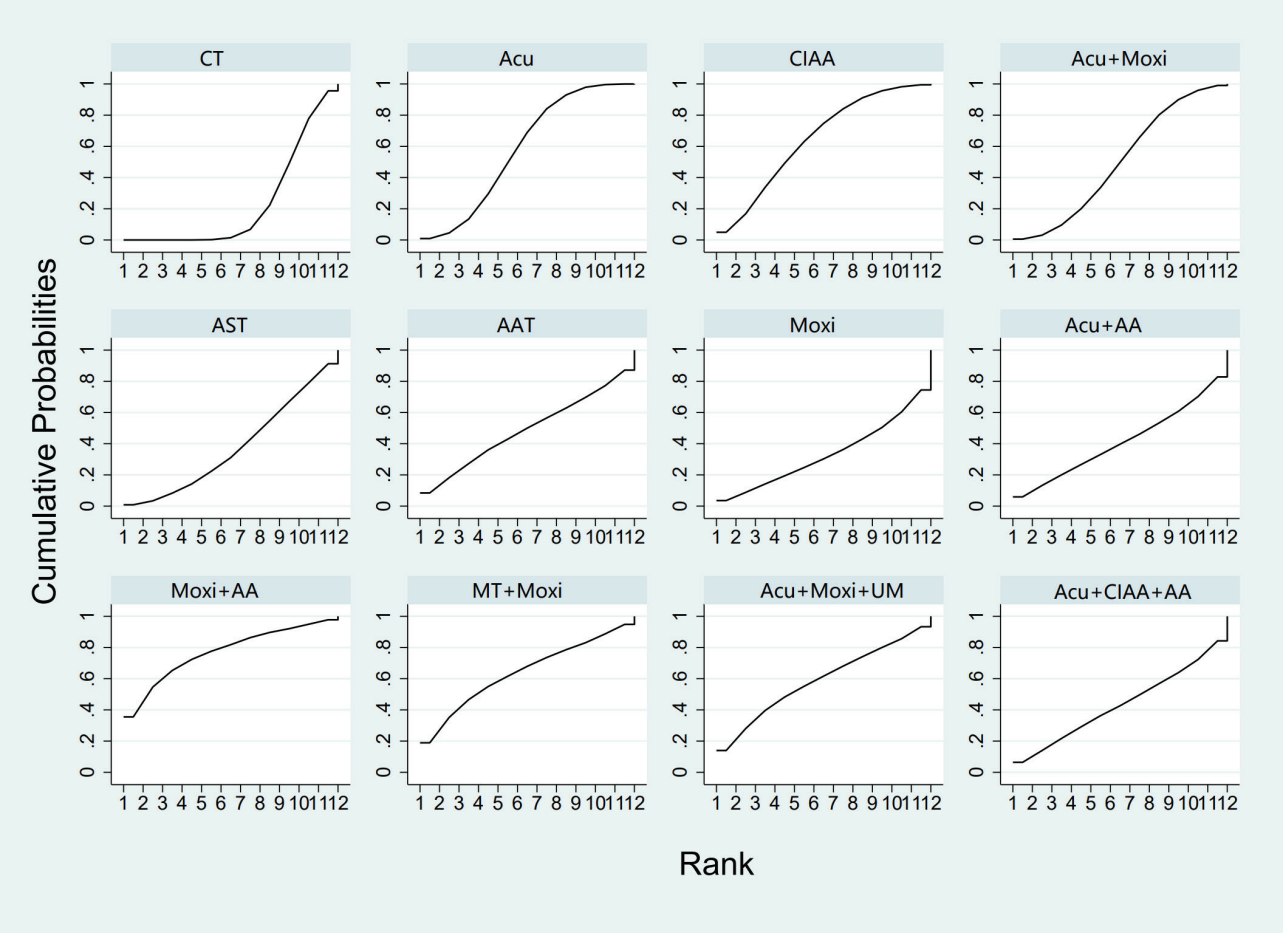
Efficacy ranking and cumulative probability graph of luteinizing hormone (LH) treated by different types of intervention. CT, conventional therapy; Acu, acupuncture; Moxi, moxibustion; AAT, abdominal acupuncture therapy; CIAA, catgut implantation at acupoint; AST, auricular seed therapy; AA, acupoint application; MT, massage therapy; UM, umbilical moxibustion.

In the evaluation of LH levels, 34 studies (23, 26–28, 30, 31, 33, 34, 39, 41, 44–46, 50–56, 58, 59, 61, 62, 64, 67, 70) involving 2,527 participants were included. The meta-analysis assessed 11 types of AS therapies and their effectiveness. Compared with the control group, the treatment that demonstrated significant improvement in LH levels was the Acu therapy [MD = 1.52, 95% CI (0.27, 2.77)]. The pairwise comparison results are shown in **Supplementary Table S6.3**. The SUCRA analysis results showed that among combination therapies, Moxi + AA therapy had the highest probability of reducing LH levels (77.1%), followed by Moxi + MT therapy (64.0%) and Acu + Moxi + UM (58.9%). Among single therapies, the CIAA therapy (64.6%) and the Acu therapy (58.3%) also had certain effects on reducing LH levels. These results are presented in **Table 2** and **Figure 6**.

#### 3.4.4 Estradiol levels

**Figure 7:**
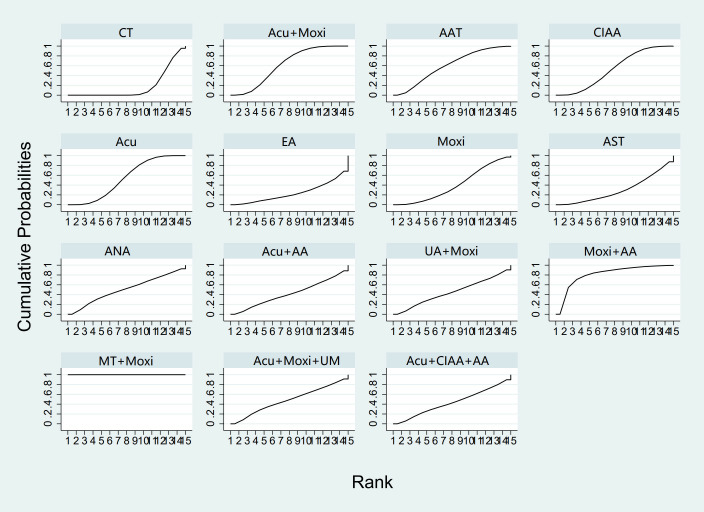
Efficacy ranking and cumulative probability graph of estradiol (E_2_) treated by different types of intervention. CT, conventional therapy; Acu, acupuncture; Moxi, moxibustion; AAT, abdominal acupuncture therapy; CIAA, catgut implantation at acupoint; EA, electroacupuncture; AST, auricular seed therapy; ANA, awn needle acupuncture; UA, umbilical acupuncture; AA, acupoint application; MT, massage therapy; UM, umbilical moxibustion.

In the evaluation of E_2_ levels, 50 studies (21–23, 25–71) involving 3,635 patients were included. The NMA examined 14 types of AS therapies and their effectiveness in comparison to conventional drug therapy or placebo. Compared with the control group, the treatments that demonstrated significant improvements in E_2_ levels were as follows: Moxi + MT therapy [MD = 11.92, 95% CI (8.19, 15.65)], Moxi + AA therapy [MD = 3.20, 95% CI (0.16, 6.24)], Acu + Moxi therapy [MD = 1.53, 95% CI (0.61, 2.44)], Acu therapy [MD = 1.27, 95% CI (0.47, 2.08)], and CIAA therapy [MD = 1.27, 95% CI (0.21, 2.34)]. Pairwise comparisons revealed that MT + Moxi therapy was superior to the other 13 therapies, with significant statistical differences (*p* < 0.05). The pairwise comparison results are shown in **Supplementary Table S6.4**. The SUCRA analysis showed that Moxi + MT therapy had the highest probability of improving E_2_ levels (100%), followed by Moxi + AA (81.4%) and Acu + Moxi (61.6%). E_2_ levels were not significantly improved by other combination therapies. As single therapies, AAT (59.7%), Acu (53.6%), and CIAA (52.9%) also had certain effects on improving E_2_ levels. The detailed results are presented in **Table 2** and **Figure 7**.

#### 3.4.5 Kupperman score

**Figure 8:**
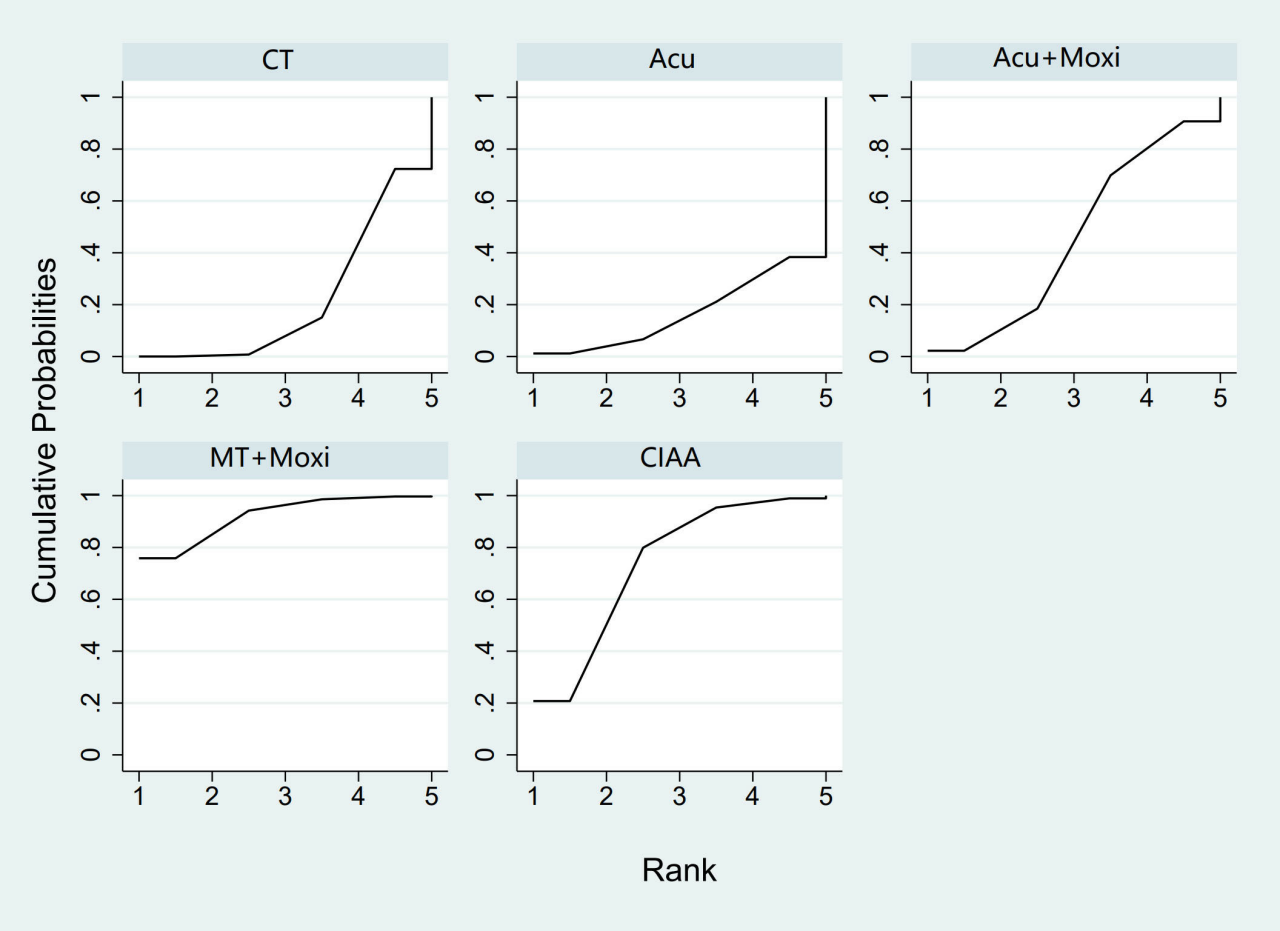
Efficacy ranking and cumulative probability graph of Kupperman scores treated by different types of intervention. CT, conventional therapy; Acu, acupuncture; Moxi, moxibustion; CIAA, catgut implantation at acupoint; MT, massage therapy.

In the evaluation of the Kupperman score, seven studies (30, 32, 46, 50, 54, 66, 70) involving 431 participants were included. The meta-analysis assessed four types of AS therapies and their effectiveness. The treatment that demonstrated significant improvement in the Kupperman scores was Moxi + MT therapy [MD = 4.63, 95% CI (0.58, 8.68)]. The pairwise comparison results are shown in **Supplementary Table S6.5**. The SUCRA analysis showed that Moxi + MT therapy had the highest probability of improving the Kupperman scores (92.1%), followed by CIAA therapy (73.8%). The detailed results are presented in **Table 2** and **Figure 8**.

### 3.5 Adverse events

A total of 13 studies (24, 26, 32, 37, 42, 50, 52–54, 56, 66, 67, 69) reported adverse events. Of these, six studies (24, 26, 37, 42, 53, 69) did not report specific adverse events, and the remaining seven studies (32, 50, 52, 54, 56, 66, 67) reported minor adverse events. All adverse events reported in these seven studies resolved after discontinuation of treatment and symptomatic treatment, which could be judged as mild adverse events, except for the adverse events reported in the study of Hou, where follow-up outcomes were not reported. There was no statistically significant difference in the incidence of adverse events in any of these studies compared with the control group. Specific details are presented in **Table 3**.

**Table 3:** Adverse events by treatment.

Adverse events	CIAA (Cases (%))	Acu (Cases (%))	Acu + moxi (Cases (%))	CT (cases (%))
Nausea		1 (1.1%	1 (1.3%)	12 (6.1%)
Headache				1 (0.5%)
Acupuncture fainting	2 (3.8%)	2 (2.3%)		
Loss of appetite				5 (2.5%)
Subcutaneous bruising	5 (9.6%)	2 (2.3%)		
Diarrhea				2 (1%)
Breast tenderness			15 (19.0%)	
Pruritus			1 (1.3%)	
Bent needle			1 (1.3%)	
Lumbar pain			2 (2.5%)	

CIAA, catgut implantation at acupoint; Acu, acupuncture; Moxi, moxibustion; CT, conventional therapy.

### 3.6 Small sample evaluation

**Figure 9:**
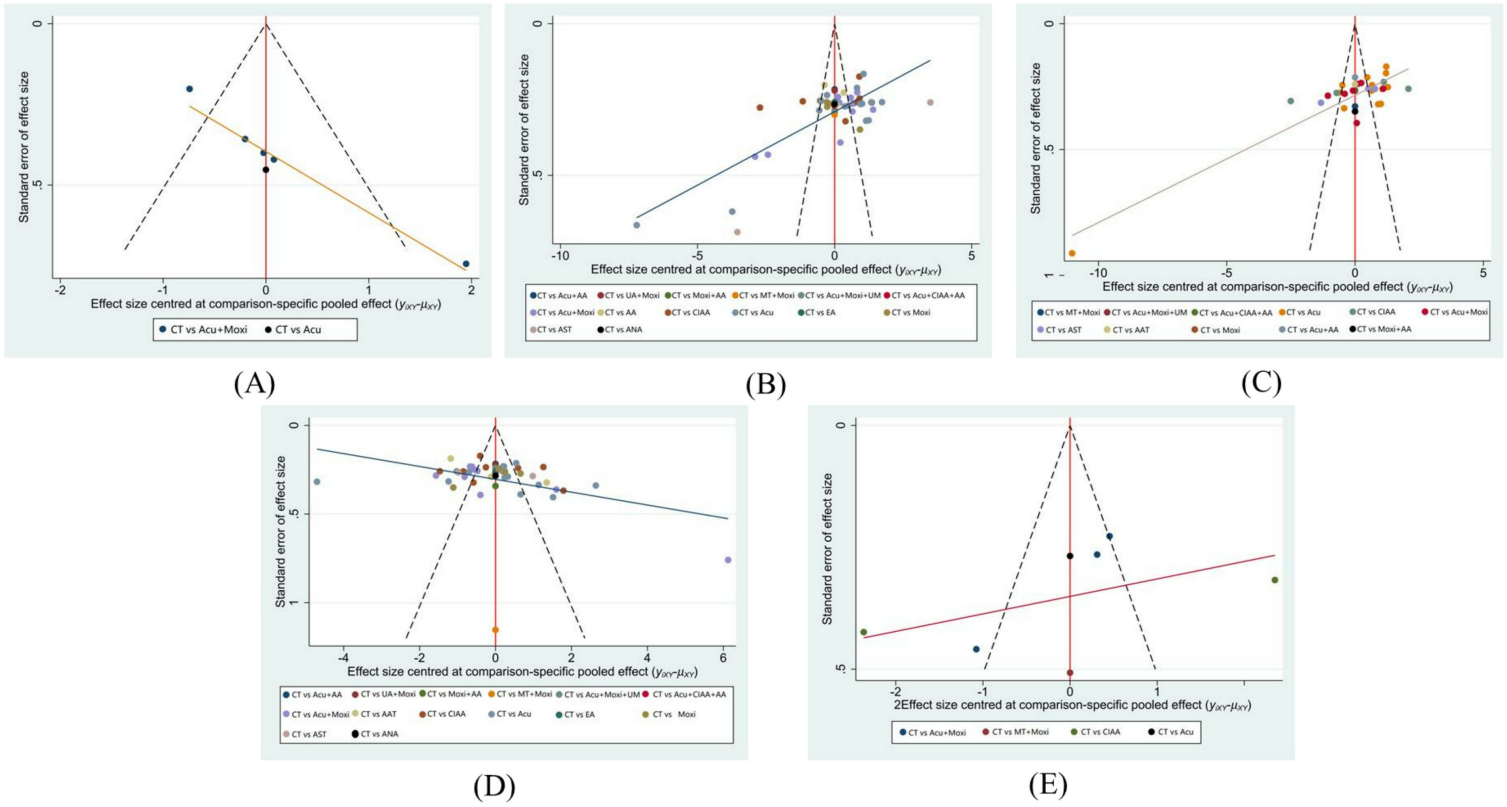
Funnel plot. **(A)** Antral follicle count (AFC); **(B)** follicle-stimulating hormone (FSH); **(C)** luteinizing hormone (LH); **(D)** estradiol (E_2_); **(E)** Kupperman score. CT, conventional therapy; Acu, acupuncture; Moxi, moxibustion; AAT, abdominal acupuncture therapy; CIAA, catgut implantation at acupoint; EA, electroacupuncture; AST, auricular seed therapy; ANA, awn needle acupuncture; UA, umbilical acupuncture; AA, acupoint application; MT, massage therapy; UM, umbilical moxibustion.

The funnel plots for LH and E_2_ demonstrate that the study points are approximately symmetrically distributed on each side of the center axis. This symmetry suggests a relatively low chance of publication bias in this study. By contrast, the funnel plots of AFC, FSH levels, and the Kupperman scores exhibit poorer symmetry, indicating potential biases or impact from small study effects. These findings are presented in **Figure 9**.

### 3.7 Assessment of the heterogeneity

The results of the heterogeneity analysis showed that all studies had an *I* ^2^ ≥ 50%, and therefore, a sensitivity analysis was performed by adopting a leave-one-out method for all outcome indicators, the results of which are presented below. Forest plots and sensitivity analysis plots are presented in **Supplementary Figures S1**–**S10**.

AFC: Du et al. (54) constituted the primary source of heterogeneity. This study employed a differential measurement protocol, using transvaginal ultrasound for married participants and transabdominal ultrasound for unmarried individuals. This methodological approach diverged from other included studies that exclusively utilized transvaginal ultrasound (all enrolled subjects had a history of sexual activity). Additionally, the inherent subjectivity in visual interpretation during AFC quantification contributed to measurement bias.
FSH: Significant heterogeneity in AAT therapy originated from study design discrepancies: Cao et al. (31) and Lai et al. (37) administered AAT therapy as an adjunct to control group therapy, whereas Li et al. (21) directly compared CT versus AAT. Heterogeneity in the Acu therapy [Wu et al. (43)/Yuan et al. (25)] and Moxi therapy [Feng et al. (28)] may be attributable to variations in treatment duration and operational techniques. The CIAA therapy [Zhang et al. (55)] introduced confounding factors using Chinese herbal medicine (rather than HRT) in the control group.
LH: Zhuang (71) exhibited heterogeneity due to suboptimal randomization methodology (odd–even number allocation). Heterogeneity in the Acu + Moxi therapy [Yuan et al. (25)/Wang et al. (64)] potentially stemmed from differences in treatment regimens, intervention protocols, or geographical factors. The CIAA therapy [Zhang et al. (55)] was similarly affected by non-standard control interventions (Chinese herbal medicine).
E_2_: AAT therapy demonstrated design-related heterogeneity analogous to FSH findings [Cao et al. (31)/Lai et al. (37) versus Li et al. (21)]. Intervention variations in the Acu + Moxi therapy [Yuan et al. (25)] and randomization flaws in the Acu therapy [Zhuang (71)] contributed to heterogeneity. The CIAA therapy [Xu et al. (22)] showed low methodological quality owing to unreported randomization and blinding procedures. Substantial treatment duration differences (3 vs. 6 months) explained heterogeneity in Moxi studies [Xu et al. (53)/Feng et al. (28)].

In summary, inadequate standardization of testing methods, differences in intervention protocol design, inconsistent regimens, flawed randomization methods, and confounding interventions in the control group were the primary sources of heterogeneity. Furthermore, the limited number of original studies stems from the inability to reliably identify sources of heterogeneity for the Kupperman score and unspecified metrics. Future investigations with adequately powered samples are required to validate these findings.

## 4 Discussion

### 4.1 Summary of main results

This study represents the first NMA to include all AS therapies in the treatment of POI. Involving a total of 51 studies with 3,754 patients, this meta-analysis examined commonly utilized AS therapies for POI and their effectiveness in comparison to conventional drug therapy or placebo. The results indicate a comparative advantage of these AS therapies over CT, particularly in improving the hormone levels and menopausal symptoms in patients with POI.

Through SUCRA, the NMA assessed the effectiveness of all AS therapies to identify the most optimal treatment plan for POI. The results suggest that moxibustion combined with other AS therapies may be the most effective in improving the hormone levels, such as the AFC, E_2_, and Kupperman scores. AST therapy has been shown to be significantly effective in improving FSH levels in patients with POI. Moxi + MT therapy benefits particularly in improving E_2_ and Kupperman scores in patients with POI.

### 4.2 Explanation of the research results

In summary, POI is a complex disorder that is caused by the interplay of genetic factors, congenital causes, autoimmune disorders, bad habits, and psychological pressure (12, 72), which lead to ovarian hypoplasia by age 40, infertility, and various menopausal symptoms. POI is recognized by elevated levels of gonadotropins and decreased levels of E_2_, which disrupt the normal menstrual cycle and lead to symptoms such as hot flashes, night sweats, insomnia, and decreased bone mineral density (73, 74). This hormonal imbalance can also lead to other related symptoms such as cardiovascular risks and mood disorders (74, 75).

Our research found that combination therapies—especially those based on moxibustion—are more successful than monotherapies in treating POI. Specifically, moxibustion combined with other treatments such as acupuncture or MT has demonstrated superior outcomes in improving the hormone levels and alleviating clinical symptoms compared to monotherapies. Moxibustion is used to warm the meridians and reconcile qi and blood by burning strips or pillars of wormwood upon acupoints near the skin’s surface (10). POI is often accompanied by hormonal imbalances and psychological symptoms. Research shows that moxibustion has been shown to significantly improve E_2_ levels and reduce FSH and LH levels, thereby enhancing ovarian function (76). Research (77) found that acupuncture with moxibustion at the lumbosacral area and acupuncture combined with warm palace moxibustion effectively alleviated the clinical symptoms and the levels of FSH and LH, while improving E_2_ levels in the treatment of patients with POI.

Multiple studies have demonstrated that moxibustion significantly reduces Kupperman scores for clinical symptoms in POI, either alone or in combination with acupuncture, with superior efficacy to pure HRT. The underlying mechanisms are hypothesized to involve multi-target regulation of the neuro–endocrine–immune network, alleviating clinical symptoms such as hot flashes, night sweats, insomnia, and anxiety and promoting menstrual cycle restoration (30, 32, 46, 50, 54, 66, 70). A study (30) showed that tuina–moxibustion combined with Chinese herbal medicine for treating the spleen and kidney yang deficiency type of POI significantly reduced its Kupperman score, which was significantly better than that of the control group. The therapy resulted in a 96.67% improvement in menstruation-related symptoms in patients with POI. Research (54) found that the treatment group (acupuncture combined with herbal UM) showed a significant reduction in Kupperman scores, which were prominently lower than those from the control group, with particularly pronounced improvements in menopause symptoms and anxiety symptoms. The study (66) showed that acupuncture combined with moxibustion in treating the liver and kidney yin deficiency type of POI resulted in a lower Kupperman score than the control group, which remained consistently lower than the control group at 3 months of follow-up. The underlying mechanism of the immediate effect and long-term efficacy may be associated with the role of moxibustion in stabilizing HPO axis homeostasis. Research (50) evaluated acupuncture combined with fire dragon moxibustion in the kidney-deficiency type of POI and found that the treatment group achieved a Kupperman score of 10.13 ± 1.96, significantly lower than that from the Western medicine group (14.52 ± 2.27). The thermal effect of fire dragon moxibustion is proposed to activate cutaneous receptors and enhance local blood circulation, thereby alleviating genitourinary and somatic symptoms.

Nine RCTs (27–29, 32, 43, 45, 50, 59, 69) included in this study had single-group sample sizes of less than 30, which is considered to be a small study. In meta-analysis, studies with smaller sample sizes tend to report larger effect sizes, whereas studies with larger sample sizes report relatively smaller effect sizes, which can lead to small-study effects. The phenomenon suggests the possibility of publication bias, selective bias, or clinical heterogeneity, which can affect the reliability of the results of systematic reviews and meta-analyses. The funnel plots of LH and E_2_ had better symmetry, indicating lower inter-study heterogeneity and better consistency of results, suggesting a low possibility of publication bias. In contrast, there was an asymmetric distribution of AFC, FSH, and Kupperman scores, suggesting that it might be related to the small sample study effect, selective publication, and heterogeneity in methodology, leading to the potential risk of bias they might have.

### 4.3 Therapeutic mechanism

Studies (72, 78) show that acupuncture may improve the ovarian function of POI rats by regulating serum metabolite markers such as divanillyltetrahydrofuran ferulate, trans-ferulic acid, tryptamine, and neuraminic acid to exert antioxidant and antiapoptotic effects, which not only increase vascular permeability by upregulating the content of vascular endothelial growth factors in serum but also inhibit the expression of the JNK-mediated apoptosis pathway-related factors under oxidative stress by downregulating ROS levels in ovarian tissue, slowing down the process of follicular atresia, and improving ovarian function in POI model rats (79, 80). Moreover, research shows that EA could improve ovarian reserve function in POI rats by reducing the number of autophagosomes and autolysosomes, upregulating p62 expression, and downregulating Beclin-1 and LC3 expression, thus inhibiting autophagy of ovarian granulosa cells, and regulating the serum levels of FSH, E_2_, AMH, and INHB (73, 81). Furthermore, studies have validated that moxibustion can improve impaired ovarian function by enhancing steroidogenesis in GCs through the activation of the cAMP/PKA/CREB pathway (74, 82), and improve ovarian reserve in rats with Cy-induced POI by attenuating mitochondrial dysfunction and NLRP3 inflammatory activation (75, 83). Recent studies indicate that Acu + Moxi can effectively improve clinical symptoms, regulate sex hormone levels, increase endometrial thickness, and increase the number of sinus follicles in patients with POI, thus promoting the recovery of ovarian function (84–86).

Autophagy, particularly the autophagic level in ovarian granulosa cells, is closely associated with POI (87). During autophagy regulating the occurrence and development of POIF, numerous autophagy-related genes and signaling pathways are involved, among which the PI3K/PTEN/AKT/mTOR pathway is relatively standard (88, 89). This pathway can influence the activation of primordial follicles by regulating the proliferation of granulosa cells in primordial follicles, and it plays a significant role in the development, growth, and atresia of primordial follicles (90). The study found that high expression of PTEN inhibits the phosphorylation of AKT. At the same time, stimulation of Guanyuan (CV4) and Sanyinjiao (SP6) can relieve the excessive inhibition of AKT by PTEN, ensuring the normal growth and development of follicles and the reserve function of the ovary (87). Research (91) demonstrated that acupuncture significantly increased the mRNA expression levels of PI3K, Akt, and mTOR in the ovaries of POF rats, accompanied by an increase in the number of mature follicles. Another study (92) indicated that acupuncture reduced granulosa cell apoptosis, which was potentially associated with the upregulation of the PI3K/Akt signaling pathway via hormonal regulation. Notably, EA was found to protect the primordial follicle pool and improve fertility in POF mice by downregulating the phosphorylation of PI3K/AKT/mTOR pathway proteins (93). Additionally, moxibustion therapy promoted ovarian function in POF rats, possibly by inhibiting the phosphorylation of the PI3K/Akt/mTOR signaling pathway (94).

### 4.4 Comparison with other studies

Existing research usually compares a particular AS treatment with a traditional medication treatment or placebo, and the present NMA of POI is quite constrained. For example, a meta-analysis in 2024 found that acupuncture substantially decreased serum levels of LH, FSH, and FSH/LH ratio, and elevated E_2_ levels (14). Another meta-analysis in 2024 on acupuncture treatment, which involved 13 RCTs, indicated that acupuncture had proven superiority over non-acupuncture in decreasing FSH levels and increasing E_2_ levels, AMH, and general efficacy in women with confirmed POI (13). However, these studies were mainly focused on acupuncture or acupuncture plus medicine, with less comparison of varied AS therapies. In our work, we include all AS therapies in the management of POI. Differing from traditional meta-analyses, NMA facilitates the identification of optimal therapy options by directly and indirectly comparing and analyzing various therapies. This study included 51 articles covering almost all existing AS therapies for POI. It systematically assessed the improvement effects of multiple AS therapies on ovarian function, sex hormone levels, and clinical symptoms. Our results are consistent with those of other meta-analyses, indicating that AS therapies—specifically acupuncture and moxibustion—are effective and safe in enhancing menopausal symptoms and hormone levels in patients with POI, as well as significantly improving their quality of life (QOL).

### 4.5 Limitations and clinical significance

There are certain limitations in this study. First, the selection criteria were confined to literature in Chinese and English, and papers that were written in other languages were excluded. This restriction may lead to failure to have a global perspective. Although our search focused on peer-reviewed journals, we acknowledge that materials such as conference abstracts and dissertations were not included due to data quality and reproducibility considerations. Second, there were only one or two studies available for some AS therapies. This lack of data might induce bias and limit the generalizability of the results. Third, it is difficult to completely blind individuals owing to the nature of the therapies. Interaction and communication between the patient and the practitioner are frequently necessary for AS therapy, and this might affect how the patient views and reacts to the treatment. Fourth, adverse events are significant for evaluating the safety of acupuncture. However, many original studies have not reported adverse events, and future research should focus on assessing the safety of acupuncture. Future multicenter, standardized RCTs with large samples and long courses of treatment are needed to extend the follow-up period and add evaluation indexes such as anti-Müllerian hormone (AMH), psychological status, and safety to further validate the efficacy and safety of AS therapy.

## 5 Conclusion

The findings from this research indicate that AS therapies are superior to control treatments in improving the hormone levels and menopausal symptoms in patients with POI. AS therapy, as a CAM therapy, can be considered a safe and effective treatment option for managing POI. In addition, AS therapies in combination with moxibustion usually results in more favorable outcomes.

## 6 Differences between the protocol and the systematic review

First, since complementary alternative therapies were too complex, it was decided to limit the intervention to AS therapies. Second, owing to the high inter-study heterogeneity, a sensitivity analysis was performed in this paper using a leave-one-out method to analyze the sources of heterogeneity. Third, because only patients with POI were included and not patients with diminished ovarian reserve (DOR), subgroup analysis could not be performed using DOR and POI as the basis for grouping. Fourth, endometrial thickness was not included in the outcome metrics because of the high variability of endometrial thickness across time.

## Data Availability

The original contributions presented in the study are included in the article/**Supplementary Material**. Further inquiries can be directed to the corresponding author.
