# Supplementary material for: Acupoint stimulation methods for premature ovarian insufficiency: a systematic review and network meta-analysis of randomized controlled trials
Source: Front Endocrinol (Lausanne). 2025 Jul 18;16:1604563. doi: 10.3389/fendo.2025.1604563 (PMC12313507; doi:10.3389/fendo.2025.1604563)
Supplement: Supplementary file 1 [file DataSheet1.docx]

Contents

[Supplementary TABLE S1: PRISMA guideline 1](#_Toc200127770)

[Supplementary TABLE S2: Search Strategy <inception to November 28, 2024> 5](#_Toc200127771)

[TABLE S2.1 Database: PubMed 5](#_Toc200127772)

[TABLE S2.2 Database: Web of science 5](#_Toc200127773)

[TABLE S2.3 Database: Embase 5](#_Toc200127774)

[TABLE S2.4 Database: Cochrane Library 6](#_Toc200127775)

[TABLE S2.5 Database: Chinese database 6](#_Toc200127776)

[Supplementary TABLE S3: Definitions of different therapies and conventional treatment 7](#_Toc200127777)

[Supplementary TABLE S4 Characteristics of the included studies 8](#_Toc200127778)

[Supplementary TABLE S5 Methodological quality evaluation table 9](#_Toc200127779)

[Supplementary TABLE S6 Results of network meta-analysis 11](#_Toc200127780)

[Supplementary TABLE S6.1 Results of network meta-analysis of AFC 11](#_Toc200127781)

[Supplementary TABLE S6.2 Results of network meta-analysis of FSH 12](#_Toc200127782)

[Supplementary TABLE S6.3 Results of network meta-analysis of LH 13](#_Toc200127783)

[Supplementary TABLE S6.4 Results of network meta-analysis of E_2_ 13](#_Toc200127784)

[Supplementary TABLE S6.5 Results of network meta-analysis of Kupperman score 14](#_Toc200127785)

[Supplementary FIGURE: Forest plots & sensitivity analysis plot 16](#_Toc200127786)

# Supplementary TABLE S1: PRISMA guideline

| **Section and Topic** | **Item #** | **Checklist item** | **Location where item is reported** |
| --- | --- | --- | --- |
| **TITLE** | | |  |
| Title | 1 | Identify the report as a systematic review. | Page 1, line 1-3 |
| **ABSTRACT** | | |  |
| Abstract | 2 | See the PRISMA 2020 for Abstracts checklist. | Page 1, line 20-62 |
| **INTRODUCTION** | | |  |
| Rationale | 3 | Describe the rationale for the review in the context of existing knowledge. | Page 3, line 64-119 |
| Objectives | 4 | Provide an explicit statement of the objective(s) or question(s) the review addresses. | Page 3, line 119-125 |
| **METHODS** | | |  |
| Eligibility criteria | 5 | Specify the inclusion and exclusion criteria for the review and how studies were grouped for the syntheses. | Page 4, line 141-169 |
| Information sources | 6 | Specify all databases, registers, websites, organisations, reference lists and other sources searched or consulted to identify studies. Specify the date when each source was last searched or consulted. | Page 4, line 131-140 |
| Search strategy | 7 | Present the full search strategies for all databases, registers and websites, including any filters and limits used. | Supplementary Table S2 |
| Selection process | 8 | Specify the methods used to decide whether a study met the inclusion criteria of the review, including how many reviewers screened each record and each report retrieved, whether they worked independently, and if applicable, details of automation tools used in the process. | Page 5, line 170-177 |
| Data collection process | 9 | Specify the methods used to collect data from reports, including how many reviewers collected data from each report, whether they worked independently, any processes for obtaining or confirming data from study investigators, and if applicable, details of automation tools used in the process. | Page 5, line 170-177 |
| Data items | 10a | List and define all outcomes for which data were sought. Specify whether all results that were compatible with each outcome domain in each study were sought (e.g. for all measures, time points, analyses), and if not, the methods used to decide which results to collect. | Page 4, line 155-161 |
|  | 10b | List and define all other variables for which data were sought (e.g. participant and intervention characteristics, funding sources). Describe any assumptions made about any missing or unclear information. | Page 6, line 216-220 |
| Study risk of bias assessment | 11 | Specify the methods used to assess risk of bias in the included studies, including details of the tool(s) used, how many reviewers assessed each study and whether they worked independently, and if applicable, details of automation tools used in the process. | Page 5, line 178-183 |
| Effect measures | 12 | Specify for each outcome the effect measure(s) (e.g. risk ratio, mean difference) used in the synthesis or presentation of results. | Page 5, line 186-188 |
| Synthesis methods | 13a | Describe the processes used to decide which studies were eligible for each synthesis (e.g. tabulating the study intervention characteristics and comparing against the planned groups for each synthesis (item #5)). | Supplementary Table S3 |
|  | 13b | Describe any methods required to prepare the data for presentation or synthesis, such as handling of missing summary statistics, or data conversions. | Page 5, line 186-188 |
|  | 13c | Describe any methods used to tabulate or visually display results of individual studies and syntheses. | Page 5, line 185-186 Page 6, line 201-202 |
|  | 13d | Describe any methods used to synthesize results and provide a rationale for the choice(s). If meta-analysis was performed, describe the model(s), method(s) to identify the presence and extent of statistical heterogeneity, and software package(s) used. | Page 5-6, line 184-202 |
|  | 13e | Describe any methods used to explore possible causes of heterogeneity among study results (e.g. subgroup analysis, meta-regression). | Page 5-6, line 191-198 |
|  | 13f | Describe any sensitivity analyses conducted to assess robustness of the synthesized results. | Page 6, line 198-200 |
| Reporting bias assessment | 14 | Describe any methods used to assess risk of bias due to missing results in a synthesis (arising from reporting biases). | None |
| Certainty assessment | 15 | Describe any methods used to assess certainty (or confidence) in the body of evidence for an outcome. | Page 5, line 187-188 |
| **RESULTS** | | |  |
| Study selection | 16a | Describe the results of the search and selection process, from the number of records identified in the search to the number of studies included in the review, ideally using a flow diagram. | Figure 1 |
|  | 16b | Cite studies that might appear to meet the inclusion criteria, but which were excluded, and explain why they were excluded. | Figure 1 |
| Study characteristics | 17 | Cite each included study and present its characteristics. | Page 6, line 213-230, Supplementary Table S3 |
| Risk of bias in studies | 18 | Present assessments of risk of bias for each included study. | Supplementary Table S5, Figure 3 |
| Results of individual studies | 19 | For all outcomes, present, for each study: (a) summary statistics for each group (where appropriate) and (b) an effect estimate and its precision (e.g. confidence/credible interval), ideally using structured tables or plots. | Page 7-10, line 255-333 |
| Results of syntheses | 20a | For each synthesis, briefly summarise the characteristics and risk of bias among contributing studies. | Page 6-7, line 231-254 |
|  | 20b | Present results of all statistical syntheses conducted. If meta-analysis was done, present for each the summary estimate and its precision (e.g. confidence/credible interval) and measures of statistical heterogeneity. If comparing groups, describe the direction of the effect. | Table 2, Figure 4-7 |
|  | 20c | Present results of all investigations of possible causes of heterogeneity among study results. | Page 9-10, line 345-379 |
|  | 20d | Present results of all sensitivity analyses conducted to assess the robustness of the synthesized results. | Supplementary Figure S6-S10 |
| Reporting biases | 21 | Present assessments of risk of bias due to missing results (arising from reporting biases) for each synthesis assessed. | Page 9, line 335-344 |
| Certainty of evidence | 22 | Present assessments of certainty (or confidence) in the body of evidence for each outcome assessed. | Supplementary Figure S1-S5 |
| **DISCUSSION** | | |  |
| Discussion | 23a | Provide a general interpretation of the results in the context of other evidence. | Page 10-13, line 385-498 |
|  | 23b | Discuss any limitations of the evidence included in the review. | Page 13-14, line 521-531 |
|  | 23c | Discuss any limitations of the review processes used. | Page 13, line 519-521 |
|  | 23d | Discuss implications of the results for practice, policy, and future research. | Page 14, line 531-541 |
| **OTHER INFORMATION** | | |  |
| Registration and protocol | 24a | Provide registration information for the review, including register name and registration number, or state that the review was not registered. | Page 4, line 130 |
|  | 24b | Indicate where the review protocol can be accessed, or state that a protocol was not prepared. | Page 4, line 130 |
|  | 24c | Describe and explain any amendments to information provided at registration or in the protocol. | Page 14, line 542-550 |
| Support | 25 | Describe sources of financial or non-financial support for the review, and the role of the funders or sponsors in the review. | Page 14, line 559-561 |
| Competing interests | 26 | Declare any competing interests of review authors. | Page 15, line 562-564 |
| Availability of data, code and other materials | 27 | Report which of the following are publicly available and where they can be found: template data collection forms; data extracted from included studies; data used for all analyses; analytic code; any other materials used in the review. | Page 15, line 565-567 |

# Supplementary TABLE S2: Search Strategy <inception to November 28, 2024>

# TABLE S2.1 Database: PubMed

| ID | Search Terms | Results |
| --- | --- | --- |
| #1 | ("Primary Ovarian Insufficiency"[Mesh]) OR (premature ovarian failure [Title/Abstract] | 5310 |
| #2 | needle*[Title/Abstract] OR acupuncture point*[Title/Abstract] OR acupoint*[Title/Abstract] OR 'Point, Acupuncture'[Title/Abstract] | 157561 |
| #3 | "Acupuncture"[Mesh] OR "Acupuncture Therapy"[Mesh] OR "Acupuncture, Ear"[Mesh] OR "Acupuncture Points"[Mesh] OR "Electroacupuncture"[Mesh] OR "Moxibustion"[Mesh] OR "Massage"[Mesh]) | 38801 |
| #4 | #2 OR #3 | 185553 |
| #5 | #1 AND #4 | 54 |

# TABLE S2.2 Database: Web of science

| ID | Search Terms | Results |
| --- | --- | --- |
| #1 | TS = (needl* OR acupuncture point* OR Cupping Treatment* OR Cupping Therap* OR acupoint* OR Massage OR Cupping Therapy OR Acupuncture OR Acupuncture Therapy OR Acupuncture Points OR Electroacupuncture OR Moxibustion) | 163969 |
| #2 | TS = (premature ovarian failure OR primary ovarian Insufficiency) | 4669 |
| #3 | #1 AND #2 | 40 |

# TABLE S2.3 Database: Embase

| ID | Search Terms | Results |
| --- | --- | --- |
| #1 | 'premature ovarian failure'/exp | 7301 |
| #2 | 'premature ovarian failure': ti,ab,kw OR ' primary ovarian Insufficiency ': ti,ab,kw | 4670 |
| #3 | 'acupuncture'/exp OR 'acupressure'/exp OR 'electroacupuncture'/exp OR 'warm acupuncture'/exp OR 'catgut embedding'/exp OR 'auricular acupuncture'/exp OR 'massage'/exp | 77698 |
| #4 | 'acupuncture': ti,ab,kw OR 'acupoint*': ti,ab,kw OR 'needl*': ti,ab,kw OR 'moxibustion': ti,ab,kw OR 'massage': ti,ab,kw OR 'Tuina': ti,ab,kw OR 'cupping': ti,ab,kw | 275574 |
| #5 | #1 OR #2 | 8843 |
| #6 | #3 OR #4 | 300799 |
| #7 | #5 AND #6 | 129 |

# TABLE S2.4 Database: Cochrane Library

| ID | Search Terms | Results |
| --- | --- | --- |
| #1 | MeSH descriptor: [Acupuncture] explode all trees | 225 |
| #2 | MeSH descriptor: [Electroacupuncture] explode all trees | 1204 |
| #3 | MeSH descriptor. [Acupuncture Points] explode all trees | 2849 |
| #4 | MeSH descriptor. [Cupping Therapy] explode all trees | 42 |
| #5 | MeSH descriptor. [Massage] explode all trees | 1744 |
| #6 | MeSH descriptor: [Primary Ovarian Insufficiency] explode all trees | 188 |
| #7 | 'acupuncture': ti,ab,kw OR 'acupoint*': ti.ab.kw OR 'needl*': ti.ab.kw OR 'moxibustion': ti.ab.kw OR 'massage': ti.ab.kw OR "Tuina': ti.ab.kw OR 'cupping': ti.ab.kw | 52274 |
| #8 | 'premature ovarian failure'.ti.ab.kw | 325 |
| #9 | #1 OR #2 OR #3 OR #4 OR #5 OR #7 | 52537 |
| #10 | #6 OR #8 | 415 |
| #11 | #9 OR #10 | 31 |

# TABLE S2.5 Database: Chinese database

| Database | Search Terms | | Results |
| --- | --- | --- | --- |
| CNKI | (TKA = ('针' + '灸' + '穴' + '推拿' + '按摩' + '罐' ) * '卵巢早衰') OR (SU =('针' + '灸' + '穴' + '推拿' + '按摩' + '罐' ) * '卵巢早衰') | | 271 |
| Wanfang | 题名或关键词:(针 or 灸 or 穴 or 推拿 or 按摩 or 罐) and 题名或关键词:(卵巢早衰) | | 295 |
| VIP | (M=针 OR M=灸 OR M=穴 OR M=罐 OR M=推拿 OR M=按摩) AND (M= 卵巢早衰） | | 653 |
| CBM | 1# | "卵巢早衰"[常用字段:智能] | 3342 |
|  | 2# | "针"[常用字段:智能] OR "灸"[常用字段:智能] OR "穴"[常用字段:智能] OR "罐"[常用字段:智能] OR "推拿"[常用字段:智能] OR "按摩"[常用字段:智能] | 989614 |
|  | 3# | 1# and 2# | 369 |

# Supplementary TABLE S3: Definitions of different therapies and conventional treatment

| abbreviation | Full name | Definitions |
| --- | --- | --- |
| CT | conventional treatment | The conventional treatment in this study is conventional hormone treatment. |
| Acu | acupuncture | Acupuncture, also known as needling or acupuncture therapy, is a traditional Chinese medical treatment method that involves stimulating specific points on the body with needles to regulate qi and blood, balance yin and yang, and promote self-healing of the body. |
| Moxi | moxibustion | Moxibustion, also known as moxa therapy or ai zhi, is a traditional Chinese medical treatment method. It involves burning moxa (dried mugwort) at specific points on the body or placing it directly on the skin to apply heat and stimulate acupuncture points, regulating qi and blood circulation, relaxing tendons, and boosting the immune system. It includes main types such as direct moxibustion, indirect moxibustion, and moxa stick moxibustion. |
| AAT | abdominal acupuncture therapy | Abdominal Acupuncture Therapy is an acupuncture treatment based on the meridian theory of Traditional Chinese Medicine (TCM), which regulates the body's visceral functions and meridian qi and blood through stimulation of specific acupuncture points in the abdomen, thereby treating systemic diseases. |
| CIAA | catgut implantation at acupoint | Catgut implantation at acupoint is a traditional Chinese medical treatment method that involves inserting one or more special threads under the skin at specific acupoints on the body, providing continuous stimulation to regulate qi and blood, promote tendon relaxation, and treat diseases. |
| EA | electroacupuncture | Electroacupuncture is a traditional Chinese medical treatment method that involves stimulating specific acupuncture points on the body using special electroacupuncture needles to regulate qi and blood, promote blood circulation, and alleviate pain, among other therapeutic purposes. |
| AST | auricular seed therapy | Auricular Seed Therapy is a traditional Chinese medicine treatment method, which belongs to the ear acupuncture therapy. It is through the specific points in the ear on the paste pressure small beans (usually WangBuLiuXing seeds or other small particles of material), the use of its continuous stimulation of the ear acupuncture points, to achieve the regulation of the human body's internal organs function, treatment of disease and health care purposes. |
| ANA | awn needle acupuncture | Awn needle acupuncture is a special kind of acupuncture therapy that uses awn needles, which are long and thin and can penetrate the skin directly and deeply to the diseased area. Its main function is to dredge the meridians and regulate qi and blood, thus achieving the purpose of treating diseases. |
| UA | umbilical acupuncture | Umbilical Acupuncture is an emerging Chinese medicine acupuncture therapy, founded by Prof. Qi Yong, based on the theory of bio-holography, which regulates the body's qi, blood and visceral functions by acupuncture at specific points in the umbilicus, thus achieving the purpose of treating diseases. |
| MT | massage therapy | Massage Therapy is a holistic health practice that involves the manual manipulation of soft tissues (such as muscles, tendons, ligaments, and fascia) to promote physical and mental well-being. It aims to relieve muscle tension, improve circulation, reduce stress, and enhance overall health and relaxation. |
| AA | acupoint application | Acupoint Application is a traditional Chinese medicine external treatment method, by applying drugs or other substances directly to specific acupoints, utilizing the medicinal effect of the drugs and the stimulating effect of the acupoints to achieve the purpose of preventing and treating diseases. |
| UM | umbilical moxibustion | Umbilical moxibustion is an external treatment that combines the meridian theory of Chinese medicine and moxibustion technique. It achieves therapeutic and healthcare purposes by applying moxa to the umbilicus of the human body (Shenque, CV8), utilizing the warmth stimulation and medicinal effect of moxa to regulate the body's qi and blood and the functions of the internal organs. |

# Supplementary TABLE S4 Characteristics of the included studies

| Author and Year | Average age | | Number of patients | | Intervention | | Treatment duration | Outcomes |
| --- | --- | --- | --- | --- | --- | --- | --- | --- |
|  | T | C | T | C | T | C |  |  |
| Liu (2024) | 35. 04±3. 12 | 34. 79±3. 49 | 36 | 35 | Acu + Moxi | HRT | 3 months | ①②③④ |
| Li (2015) | 32.5±6.4 | 34.2±4.2 | 30 | 30 | Acu | TCM | 3 months | ②④ |
| Xu (2008) | NA | NA | 33 | 33 | CIAA | HRT | 6 months | ②④ |
| Xiao (2015) | 35.4 | 35.4 | 20 | 20 | Acu | HRT | 6 months | ②③④ |
| Chen (2019) | 34.22±4.18 | 34.22±4.18 | 30 | 30 | CIAA | TCM | 6 months | ②③④ |
| Luo (2015) | 30.4±9.0 | 29.34±8.74 | 30 | 30 | EA | HRT | 3 months | ②④ |
| Wu (2018) | 35.6±3.1 | 35.4±3.2 | 34 | 34 | Acu + Moxi | HRT | 3 months | ②③④ |
| Zhou (2022) | 32.19±4.36 | 32.45±4.44 | 30 | 30 | Acu | TCM | 3 months | ①②③④ |
| Wu (2019) | 31.14±3.62 | 32.08±3.21 | 30 | 30 | Acu | HRT | 3 weeks | ②③④⑤ |
| Xu (2019) | 35.2±2.3 | 35.4±1.9 | 35 | 35 | Moxi | TCM | 6 months | ②④ |
| Zhong (2018) | NA | NA | 30 | 30 | AST | TCM | 3 months | ②③④ |
| Yi (2024) | 27.41±2.46 | 27.35±2.11 | 40 | 40 | Acu + Moxi | TCM | 1 month | ①②③④ |
| Cao (2015) | 34.2±2.2 | 34.5±2.6 | 40 | 40 | AAT | TCM | 6 months | ②③④ |
| Feng (2019) | 34 | 33 | 25 | 25 | Moxi | HRT | 3 months | ②③④ |
| Fu (2015) | 33.21±4.28 | 32.17±4.32 | 48 | 48 | Acu | HRT | 6 months | ②③④ |
| Dong (2013) | NA | NA | 30 | 30 | ANA | HRT | 1 month | ②④ |
| Xu (2024) | 32.05±4.96 | 31.65±5.32 | 46 | 46 | Acu + AA | HRT | 3 months | ②③④ |
| Du (2022) | 32.5±4.6 | 32.0±4.5 | 40 | 40 | Acu + Moxi | HRT | 3 months | ①②④⑤ |
| Yu (2019) | 29.2±4.4 | 29.8±4.5 | 45 | 45 | UA + Moxi | HRT | 3 months | ②④ |
| Yang (2021) | 30±4 | 29±5 | 37 | 37 | Moxi | HRT | 1 month | ②④ |
| Wu (2023) | 31.23±3.13 | 31.52±3.08 | 40 | 40 | Moxi + AA | TCM | 1.5 months | ②③④ |
| Zhang (2023) | 34.32±6.127 | 32.88±5.389 | 74 | 74 | CIAA | TCM | 1.5 months | ②④ |
| Wu (2017) | 35.90±3.53 | 35.50±3.82 | 30 | 30 | MT + Moxi | TCM | 2 months | ②③④⑤ |
| Wang (2021) | 31.67±4.59 | 31.91±5.31 | 33 | 33 | Moxi | HRT | 1.5 months | ②④ |
| Wang (2022) | 32.6±3.5 | 32.7±3.8 | 40 | 40 | Acu + Moxi | HRT | 4 months | ②③④ |
| Duan (2024) | 31.35±4.57 | 31.19±4.68 | 34 | 34 | Acu + Moxi | HRT | 6 months | ②③④ |
| Zhang (2023) | 34.8±3.7 | 35.2±4.3 | 40 | 40 | Acu + Moxi + UM | HRT | 3 months | ②③④ |
| Luo (2020) | 38.46±6.83 | 38.13±6.52 | 50 | 50 | CIAA | TCM | 2 months | ②③④⑤ |
| Li (2014) | NA | NA | 33 | 32 | CIAA | HRT | 6 months | ②④ |
| Tan (2017) | 35.4±4.2 | 34.2±4.8 | 22 | 22 | CIAA | HRT | 4 months | ②④⑤ |
| Bian (2016) | 34.94±3.44 | 35.10±3.41 | 30 | 30 | CIAA | HRT | 3 months | ②③④ |
| Liu (2008) | NA | NA | 66 | 66 | CIAA | HRT | 6 months | ②④ |
| Wang (2011) | 35.35±9.62 | 34.92±9.34 | 20 | 20 | Acu | TCM | 3 months | ②③④ |
| Zhu (2021) | 36.13±3.64 | 34.21±3.18 | 25 | 25 | Acu | HRT | 3 months | ②③④ |
| Zhuang (2024) | 31.26±3.12 | 30.87±3.37 | 15 | 15 | Acu + Moxi | HRT | 3 months | ②③④⑤ |
| Xie (2020) | 32.53±3.41 | 32.73±3.52 | 30 | 30 | Acu + Moxi | HRT | 4 months | ②④⑤ |
| Liu (2019) | 34.4±1.3 | 34.2±1.4 | 64 | 64 | Acu | HRT | 6 months | ②③④ |
| Zhao (2024) | 34.86±2.11 | 35.29±2.04 | 31 | 31 | Acu + CIAA + AA | HRT | 3 months | ②③④ |
| Hou (2019) | 33±3 | 33±3 | 48 | 48 | Acu | HRT | 2 months | ②③④ |
| Lu (2015) | 34.25±4.40 | 34.25±4.40 | 32 | 32 | Acu | HRT | 3 months | ②④ |
| Yao (2019) | 35±1 | 36±1 | 52 | 52 | Acu | HRT | 3 months | ②③④ |
| Zhuang (2020) | 25.12±1.31 | 24.52±1.35 | 45 | 45 | Acu | TCM | 2 months | ②③④ |
| Yang (2008) | 35.5 | 35.5 | 30 | 30 | Acu | HRT | 6 months | ②③④ |
| Sha (1999) | 35.5 | 35.5 | 76 | 75 | Acu | HRT | 6 months | ②③④ |
| Yuan (2022) | 30.70±3.04 | ( 30． 90 ± 2． 99 | 30 | 30 | Acu + Moxi | HRT | 3 months | ①②③④ |
| Zhang (2024) | 31.84±4.4 | 32.13±3.92 | 32 | 31 | Acu | HRT | 6 months | ②③④ |
| Lai (2017) | 39.5±8.5 | 39.5±8.5 | 58 | 58 | AAT | TCM | 3 months | ②④ |
| Ye (2014) | NA | NA | 30 | 30 | AST | TCM | 3 months | ②③④ |
| Wu (2018) | 35±4 | 36±4 | 25 | 25 | Acu + Moxi | HRT | 3 months | ①②③④ |
| Liu (2021) | 35.58±3.21 | 35.61±3.18 | 25 | 25 | Acu + Moxi | HRT | 3 months | ②④ |
| Teng (2018) | 31.95±5.38 | 32.71±4.61 | 30 | 30 | Acu | TCM | 1.5 months | ②③ |

Note: T, trial group; C, control group; NA, not available; HRT, hormone replacement therapy; TCM, traditional Chinese medicine; ①antral follicle count (AFC); ②follicle-stimulating hormone (FSH); ③luteinizing hormone (LH); ④estradiol (E_2_); ⑤Kupperman score

# Supplementary TABLE S5 Methodological quality evaluation table

| Articles | Random sequence generation | Allocation concealment | Blinding of participants and personnel | Blinding of outcome assessment | Incomplete outcome data | Selective outcome reporting | Other |
| --- | --- | --- | --- | --- | --- | --- | --- |
| Liu (2024) | Low | Unclear | Unclear | Unclear | Low | Low | Low |
| Li (2015) | Low | Unclear | Unclear | Unclear | Low | Low | Unclear |
| Xu (2008) | Unclear | Unclear | Unclear | Unclear | Low | Low | Unclear |
| Xiao (2015) | Unclear | Unclear | Unclear | Unclear | Low | Low | Unclear |
| Chen (2019) | Unclear | Unclear | Unclear | Unclear | Low | Low | Unclear |
| Luo (2015) | Low | Unclear | Unclear | Unclear | Low | Low | Unclear |
| Wu (2018) | Low | Unclear | Unclear | Unclear | Low | Low | Unclear |
| Zhou (2022) | Low | Unclear | Unclear | Unclear | Low | Low | Unclear |
| Wu (2019) | Unclear | Unclear | Unclear | Unclear | Low | Low | Unclear |
| Xu (2019) | Low | Unclear | Unclear | Unclear | Low | Low | Unclear |
| Zhong (2018) | High | Unclear | Unclear | Unclear | Low | Low | Unclear |
| Yi (2024) | Unclear | Unclear | Unclear | Unclear | Low | Low | Unclear |
| Cao (2015) | Unclear | Unclear | Unclear | Unclear | Low | Low | Unclear |
| Feng (2019) | Low | Unclear | Unclear | Unclear | Low | Low | Unclear |
| Fu (2015) | Unclear | Unclear | Unclear | Unclear | Low | Low | Unclear |
| Dong (2013) | Low | Unclear | Unclear | Unclear | Low | Low | Unclear |
| Xu (2024) | Low | Unclear | Unclear | Unclear | Low | Low | Unclear |
| Du (2022) | Low | Unclear | Unclear | Unclear | Low | Low | Unclear |
| Yu (2019) | Low | Unclear | Unclear | Unclear | Low | Low | Unclear |
| Yang (2021) | Low | Unclear | Unclear | Unclear | Low | Low | Unclear |
| Wu (2023) | Low | Unclear | Unclear | Unclear | Low | Low | Unclear |
| Zhang (2023) | Low | Unclear | Unclear | Unclear | Low | Low | Unclear |
| Wu (2017) | Unclear | Unclear | Low | Unclear | Low | Low | Unclear |
| Wang (2021) | Low | Unclear | Unclear | Unclear | Low | Low | Unclear |
| Wang (2022) | Low | Unclear | Unclear | Unclear | Low | Low | Unclear |
| Duan (2024) | Low | Unclear | Unclear | Unclear | Low | Low | Unclear |
| Zhang (2023) | Low | Unclear | Unclear | Unclear | Low | Low | Unclear |
| Luo (2020) | High | Unclear | Unclear | Unclear | Low | Low | Unclear |
| Li (2014) | Low | Unclear | Unclear | Unclear | Low | Low | Unclear |
| Tan (2017) | Unclear | Unclear | Unclear | Unclear | Low | Low | Unclear |
| Bian (2016) | High | Unclear | Unclear | Unclear | Low | Low | Unclear |
| Liu (2008) | High | Unclear | Unclear | Unclear | Low | Low | Unclear |
| Wang (2011) | High | Unclear | Unclear | Unclear | Low | Low | Unclear |
| Zhu (2021) | Low | Unclear | Unclear | Unclear | Low | Low | Unclear |
| Zhuang (2024) | Low | Unclear | Unclear | Unclear | Low | Low | Unclear |
| Xie (2020) | Low | Unclear | Unclear | Unclear | Low | Low | Unclear |
| Liu (2019) | Low | Unclear | Unclear | Unclear | Low | Low | Unclear |
| Zhao (2024) | Low | Unclear | Unclear | Unclear | Low | Low | Unclear |
| Hou (2019) | Low | Unclear | Unclear | Unclear | Low | Low | Unclear |
| Lu (2015) | Unclear | Unclear | Unclear | Unclear | Low | Low | Unclear |
| Yao (2019) | Low | Unclear | Unclear | Unclear | Low | Low | Unclear |
| Zhuang (2020) | High | Unclear | Unclear | Unclear | Low | Low | Unclear |
| Yang (2008) | Unclear | Unclear | Unclear | Unclear | Low | Low | Unclear |
| Sha (1999) | Unclear | Unclear | Unclear | Unclear | Low | Low | Unclear |
| Yuan (2022) | Low | Unclear | Unclear | Unclear | Low | Low | Unclear |
| Zhang (2024) | Unclear | Unclear | Unclear | Unclear | Low | Low | Unclear |
| Lai (2017) | Low | Unclear | Unclear | Unclear | Low | Low | Unclear |
| Ye (2014) | Unclear | Unclear | Unclear | Unclear | Low | Low | Unclear |
| Wu (2018) | Low | Unclear | Unclear | Unclear | Low | Low | Unclear |
| Liu (2021) | Low | Unclear | Unclear | Unclear | Low | Low | Unclear |
| Teng (2018) | Unclear | Unclear | Unclear | Unclear | Low | Low | Unclear |

# Supplementary TABLE S6 Results of network meta-analysis

# Supplementary TABLE S6.1 Results of network meta-analysis of AFC

| Interventions | CT | Acu | Acu + Moxi |
| --- | --- | --- | --- |
| Acu + Moxi | 2.04 (1.31,2.77) | 0.02 (-1.72,1.76) | 0 |
| Acu | 2.02 (0.45,3.59) | 0 |  |
| CT | 0 |  |  |

# Supplementary TABLE S6.2 Results of network meta-analysis of FSH

| Interventions | AST | Moxi + AAT | Acu + Moxi + UM | Acu | MT + Moxi | Acu + Moxi | CIAA | Acu + CIAA + AAT | Moxi | UA + Moxi | AAT | Acu + AAT | ANA | EA | CT |
| --- | --- | --- | --- | --- | --- | --- | --- | --- | --- | --- | --- | --- | --- | --- | --- |
| CT | 3.03(0.45,5.60) | 2.29(-1.26,5.85) | 2.17(-1.38,5.72) | 1.64(0.72,2.56) | 1.62(-1.93,5.18) | 1.27(0.20,2.35) | 1.05(-0.20,2.31) | 0.99(-2.55,4.54) | 1.03(-0.75,2.80) | 0.84(-2.69,4.37) | 0.81(-1.23,2.85) | 0.77(-2.76,4.30) | 0.66(-2.89,4.20) | -0.12(-3.66,3.42) | 0 |
| EA | 3.15(-1.23,7.53) | 2.42(-2.60,7.43) | 2.30(-2.72,7.31) | 1.76(-1.90,5.42) | 1.74(-3.28,6.76) | 1.39(-2.31,5.10) | 1.18(-2.58,4.93) | 1.12(-3.90,6.13) | 1.15(-2.81,5.11) | 0.96(-4.04,5.96) | 0.93(-3.16,5.02) | 0.89(-4.11,5.89) | 0.78(-4.23,5.79) | 0 |  |
| ANA | 2.37(-2.02,6.75) | 1.64(-3.38,6.65) | 1.52(-3.50,6.53) | 0.98(-2.68,4.64) | 0.96(-4.06,5.98) | 0.62(-3.09,4.32) | 0.40(-3.36,4.16) | 0.34(-4.68,5.35) | 0.37(-3.59,4.33) | 0.18(-4.82,5.18) | 0.15(-3.94,4.24) | 0.11(-4.89,5.11) | 0 |  |  |
| Acu + AAT | 2.26(-2.11,6.63) | 1.53(-3.48,6.54) | 1.41(-3.60,6.41) | 0.87(-2.78,4.52) | 0.85(-4.16,5.86) | 0.51(-3.18,4.20) | 0.29(-3.46,4.03) | 0.23(-4.78,5.23) | 0.26(-3.69,4.21) | 0.07(-4.93,5.06) | 0.04(-4.04,4.12) | 0 |  |  |  |
| AAT | 2.22(-1.07,5.51) | 1.48(-2.61,5.58) | 1.37(-2.73,5.46) | 0.83(-1.41,3.07) | 0.81(-3.29,4.91) | 0.46(-1.84,2.77) | 0.25(-2.15,2.64) | 0.18(-3.91,4.28) | 0.22(-2.49,2.92) | 0.03(-4.05,4.11) | 0 |  |  |  |  |
| UA + Moxi | 2.19(-2.18,6.56) | 1.46(-3.55,6.47) | 1.34(-3.67,6.35) | 0.80(-2.85,4.45) | 0.78(-4.23,5.79) | 0.44(-3.26,4.13) | 0.22(-3.53,3.96) | 0.16(-4.85,5.16) | 0.19(-3.76,4.14) | 0 |  |  |  |  |  |
| Moxi | 2.00(-1.13,5.13) | 1.27(-2.71,5.24) | 1.15(-2.82,5.12) | 0.61(-1.39,2.61) | 0.59(-3.38,4.57) | 0.25(-1.83,2.32) | 0.03(-2.15,2.20) | -0.03(-4.00,3.93) | 0 |  |  |  |  |  |  |
| Acu + CIAA + AAT | 2.03(-2.35,6.42) | 1.30(-3.72,6.32) | 1.18(-3.84,6.20) | 0.64(-3.02,4.31) | 0.63(-4.39,5.65) | 0.28(-3.42,3.98) | 0.06(-3.70,3.82) | 0 |  |  |  |  |  |  |  |
| CIAA | 1.97(-0.89,4.84) | 1.24(-2.53,5.01) | 1.12(-2.64,4.88) | 0.58(-0.97,2.14) | 0.57(-3.20,4.34) | 0.22(-1.43,1.87) | 0 |  |  |  |  |  |  |  |  |
| Acu + Moxi | 1.75(-1.04,4.55) | 1.02(-2.69,4.73) | 0.90(-2.81,4.61) | 0.36(-1.05,1.78) | 0.35(-3.37,4.06) | 0 |  |  |  |  |  |  |  |  |  |
| MT + Moxi | 1.41(-2.99,5.80) | 0.67(-4.35,5.70) | 0.55(-4.47,5.58) | 0.02(-3.66,3.69) | 0 |  |  |  |  |  |  |  |  |  |  |
| Acu | 1.39(-1.35,4.13) | 0.66(-3.01,4.33) | 0.54(-3.13,4.20) | 0 |  |  |  |  |  |  |  |  |  |  |  |
| Acu + Moxi + UM | 0.85(-3.54,5.24) | 0.12(-4.90,5.14) | 0 |  |  |  |  |  |  |  |  |  |  |  |  |
| Moxi + AAT | 0.73(-3.66,5.12) | 0 |  |  |  |  |  |  |  |  |  |  |  |  |  |
| AST | 0 |  |  |  |  |  |  |  |  |  |  |  |  |  |  |

Note: CT: conventional therapy, Acu: acupuncture, Moxi: moxibustion, AAT: abdominal acupuncture therapy, CIAA: catgut implantation at acupoint, EA: electroacupuncture, AST: auricular seed therapy, ANA: awn needle acupuncture, UA: umbilical acupuncture, AA: acupoint application, MT: massage therapy, UM: umbilical moxibustion; AFC: antral follicle count; FSH: follicle-stimulating hormone; LH: luteinizing hormone; E_2_: estradiol.

# Supplementary TABLE S6.3 Results of network meta-analysis of LH

| Interventions | Moxi + AA | CIAA | MT + Moxi | Acu + Moxi + UM | Acu | Acu + Moxi | AAT | Acu + CIAA + AA | Acu + AA | AST | Moxi | CT |
| --- | --- | --- | --- | --- | --- | --- | --- | --- | --- | --- | --- | --- |
| CT | 3.31 (-1.20,7.81) | 1.90 (-0.34,4.14) | 2.15 (-2.35,6.65) | 1.78 (-2.70,6.26) | 1.52 (0.27,2.77) | 1.15 (-0.54,2.85) | 1.10 (-3.38,5.57) | 0.80 (-3.68,5.28) | 0.58 (-3.89,5.05) | 0.58 (-2.01,3.17) | 0.05 (-4.45,4.55) | 0 |
| Moxi | 3.26 (-3.11,9.62) | 1.85 (-3.17,6.88) | 2.10 (-4.26,8.47) | 1.73 (-4.62,8.09) | 1.47 (-3.20,6.15) | 1.11 (-3.71,5.92) | 1.05 (-5.30,7.40) | 0.75 (-5.60,7.10) | 0.53 (-5.81,6.87) | 0.53 (-4.66,5.72) | 0 |  |
| AST | 2.73 (-2.47,7.92) | 1.33 (-2.10,4.75) | 1.57 (-3.62,6.76) | 1.20 (-3.97,6.38) | 0.94 (-1.93,3.82) | 0.58 (-2.52,3.67) | 0.52 (-4.65,5.69) | 0.22 (-4.95,5.40) | 0.00 (-5.16,5.17) | 0 |  |  |
| Acu + AA | 2.73 (-3.62,9.07) | 1.32 (-3.68,6.32) | 1.57 (-4.77,7.91) | 1.20 (-5.13,7.53) | 0.94 (-3.70,5.59) | 0.58 (-4.20,5.36) | 0.52 (-5.80,6.85) | 0.22 (-6.11,6.55) | 0 |  |  |  |
| Acu + CIAA + AA | 2.51 (-3.85,8.86) | 1.10 (-3.91,6.11) | 1.35 (-5.00,7.70) | 0.98 (-5.35,7.32) | 0.72 (-3.93,5.37) | 0.35 (-4.44,5.14) | 0.30 (-6.03,6.63) | 0 |  |  |  |  |
| AAT | 2.21 (-4.14,8.55) | 0.80 (-4.20,5.81) | 1.05 (-5.29,7.40) | 0.68 (-5.65,7.02) | 0.42 (-4.22,5.07) | 0.06 (-4.73,4.84) | 0 |  |  |  |  |  |
| Acu + Moxi | 2.15 (-2.66,6.96) | 0.75 (-2.06,3.56) | 1.00 (-3.81,5.80) | 0.63 (-4.16,5.42) | 0.37 (-1.74,2.47) | 0 |  |  |  |  |  |  |
| Acu | 1.78 (-2.89,6.46) | 0.38 (-2.18,2.95) | 0.63 (-4.04,5.30) | 0.26 (-4.39,4.91) | 0 |  |  |  |  |  |  |  |
| Acu + Moxi + UM | 1.52 (-4.83,7.88) | 0.12 (-4.89,5.13) | 0.37 (-5.98,6.72) | 0 |  |  |  |  |  |  |  |  |
| MT + Moxi | 1.15 (-5.21,7.52) | 0.25 (-5.27,4.78) | 0 |  |  |  |  |  |  |  |  |  |
| CIAA | 1.40 (-3.63,6.43) | 0 |  |  |  |  |  |  |  |  |  |  |
| Moxi + AA | 0 |  |  |  |  |  |  |  |  |  |  |  |

Note: CT: conventional therapy, Acu: acupuncture, Moxi: moxibustion, AAT: abdominal acupuncture therapy, CIAA: catgut implantation at acupoint, AST: auricular seed therapy, AA: acupoint application, MT: massage therapy, UM: umbilical moxibustion; LH: luteinizing hormone.

# Supplementary TABLE S6.4 Results of network meta-analysis of E_2_

| Interventions | MT + Moxi | Moxi + AA | Acu + Moxi | AAT | Acu | CIAA | ANA | Acu + Moxi + UM | UA + Moxi | Acu + CIAA + AA | Acu + AA | Moxi | AST | EA | CT |
| --- | --- | --- | --- | --- | --- | --- | --- | --- | --- | --- | --- | --- | --- | --- | --- |
| CT | 11.92 (8.19,15.65) | 3.20 (0.16,6.24) | 1.53 (0.61,2.44) | 1.52 (-0.22,3.26) | 1.27 (0.47,2.08) | 1.27 (0.21,2.34) | 1.26 (-1.76,4.28) | 1.09 (-1.92,4.09) | 0.95 (-2.05,3.94) | 0.85 (-2.16,3.86) | 0.80 (-2.20,3.79) | 0.80 (-0.71,2.31) | 0.32 (-1.81,2.45) | -0.19 (-3.20,2.82) | 0 |
| EA | 12.11 (7.32,16.90) | 3.38 (-0.89,7.66) | 1.71 (-1.43,4.86) | 1.71 (-1.76,5.19) | 1.46 (-1.65,4.58) | 1.46 (-1.73,4.65) | 1.45 (-2.81,5.71) | 1.27 (-2.98,5.53) | 1.13 (-3.11,5.38) | 1.04 (-3.22,5.29) | 0.99 (-3.26,5.23) | 0.99 (-2.38,4.35) | 0.51 (-3.18,4.20) | 0 |  |
| AST | 11.60 (7.30,15.89) | 2.88 (-0.84,6.59) | 1.20 (-1.12,3.53) | 1.20 (-1.55,3.96) | 0.95 (-1.33,3.23) | 0.95 (-1.43,3.33) | 0.94 (-2.76,4.64) | 0.77 (-2.92,4.45) | 0.63 (-3.05,4.30) | 0.53 (-3.16,4.22) | 0.48 (-3.20,4.15) | 0.48 (-2.14,3.09) | 0 |  |  |
| Moxi | 11.12 (7.10,15.14) | 2.40 (-1.00,5.79) | 0.73 (-1.04,2.49) | 0.73 (-1.58,3.03) | 0.48 (-1.23,2.19) | 0.47 (-1.37,2.32) | 0.46 (-2.91,3.84) | 0.29 (-3.07,3.65) | 0.15 (-3.21,3.51) | 0.05 (-3.32,3.42) | 0.00 (-3.36,3.36) | 0 |  |  |  |
| Acu + AA | 11.12 (6.34,15.91) | 2.40 (-1.87,6.67) | 0.73 (-2.41,3.86) | 0.73 (-2.74,4.19) | 0.48 (-2.63,3.58) | 0.47 (-2.71,3.65) | 0.46 (-3.79,4.72) | 0.29 (-3.95,4.53) | 0.15 (-4.09,4.39) | 0.05 (-4.20,4.30) | 0 |  |  |  |  |
| Acu + CIAA + AA | 11.07 (6.28,15.86) | 2.35 (-1.93,6.63) | 0.68 (-2.47,3.83) | 0.68 (-2.80,4.15) | 0.43 (-2.69,3.54) | 0.42 (-2.77,3.62) | 0.41 (-3.85,4.68) | 0.24 (-4.01,4.49) | 0.10 (-4.15,4.35) | 0 |  |  |  |  |  |
| UA + Moxi | 10.97 (6.19,15.76) | 2.25 (-2.02,6.52) | 0.58 (-2.56,3.71) | 0.58 (-2.89,4.04) | 0.33 (-2.78,3.43) | 0.33 (-2.86,3.51) | 0.31 (-3.94,4.57) | 0.14 (-4.10,4.38) | 0 |  |  |  |  |  |  |
| Acu + Moxi + UM | 10.83 (6.05,15.62) | 2.11 (-2.16,6.38) | 0.44 (-2.70,3.58) | 0.44 (-3.03,3.91) | 0.19 (-2.92,3.30) | 0.19 (-3.00,3.37) | 0.17 (-4.08,4.43) | 0 |  |  |  |  |  |  |  |
| ANA | 10.66 (5.86,15.46) | 1.94 (-2.35,6.22) | 0.26 (-2.89,3.42) | 0.26 (-3.22,3.75) | 0.01 (-3.11,3.14) | 0.01 (-3.19,3.21) | 0 |  |  |  |  |  |  |  |  |
| CIAA | 10.65 (6.77,14.53) | 1.92 (-1.30,5.15) | 0.25 (-1.15,1.66) | 0.25 (-1.79,2.29) | 0.00 (-1.33,1.34) | 0 |  |  |  |  |  |  |  |  |  |
| Acu | 10.64 (6.83,14.46) | 1.92 (-1.22,5.07) | 0.25 (-0.97,1.47) | 0.25 (-1.67,2.17) | 0 |  |  |  |  |  |  |  |  |  |  |
| AAT | 10.40 (6.28,14.51) | 1.67 (-1.83,5.18) | 0.00 (-1.97,1.97) | 0 |  |  |  |  |  |  |  |  |  |  |  |
| Acu + Moxi | 10.39 (6.55,14.23) | 1.67 (-1.51,4.85) | 0 |  |  |  |  |  |  |  |  |  |  |  |  |
| Moxi + AA | 8.72 (3.91,13.53) | 0 |  |  |  |  |  |  |  |  |  |  |  |  |  |
| MT + Moxi | 0 |  |  |  |  |  |  |  |  |  |  |  |  |  |  |

Note: CT: Conventional Therapy, Acu: Acupuncture, Moxi: Moxibustion, AAT: Abdominal Acupuncture Therapy, CIAA: Catgut Implantation at Acupoint, EA: Electroacupuncture, AST: Auricular Seed Therapy, ANA: Awn Needle Acupuncture, UA: Umbilical Acupuncture, AA: Acupoint Application; MT: Massage Therapy, UM: Umbilical Moxibustion; E_2_: estradiol.

# Supplementary TABLE S6.5 Results of network meta-analysis of Kupperman score

| Interventions | MT + Moxi | CIAA | Acu + Moxi | CT | Acu |
| --- | --- | --- | --- | --- | --- |
| Acu | 5.38 (-0.29,11.05) | 3.56 (-1.31,8.43) | 1.78 (-2.80,6.37) | 0.75 (-3.21,4.72) | 0 |
| CT | 4.63 (0.58,8.68) | 2.80 (-0.02,5.63) | 1.03 (-1.27,3.33) | 0 |  |
| Acu + Moxi | 3.60 (-1.06,8.26) | 1.77 (-1.87,5.42) | 0 |  |  |
| CIAA | 1.83 (-3.12,6.77) | 0 |  |  |  |
| MT + Moxi | 0 |  |  |  |  |

Note: CT: conventional therapy, Acu: acupuncture, Moxi: moxibustion, CIAA: catgut implantation at acupoint, MT: massage therapy.

# Supplementary FIGURE: Forest plots & sensitivity analysis plot


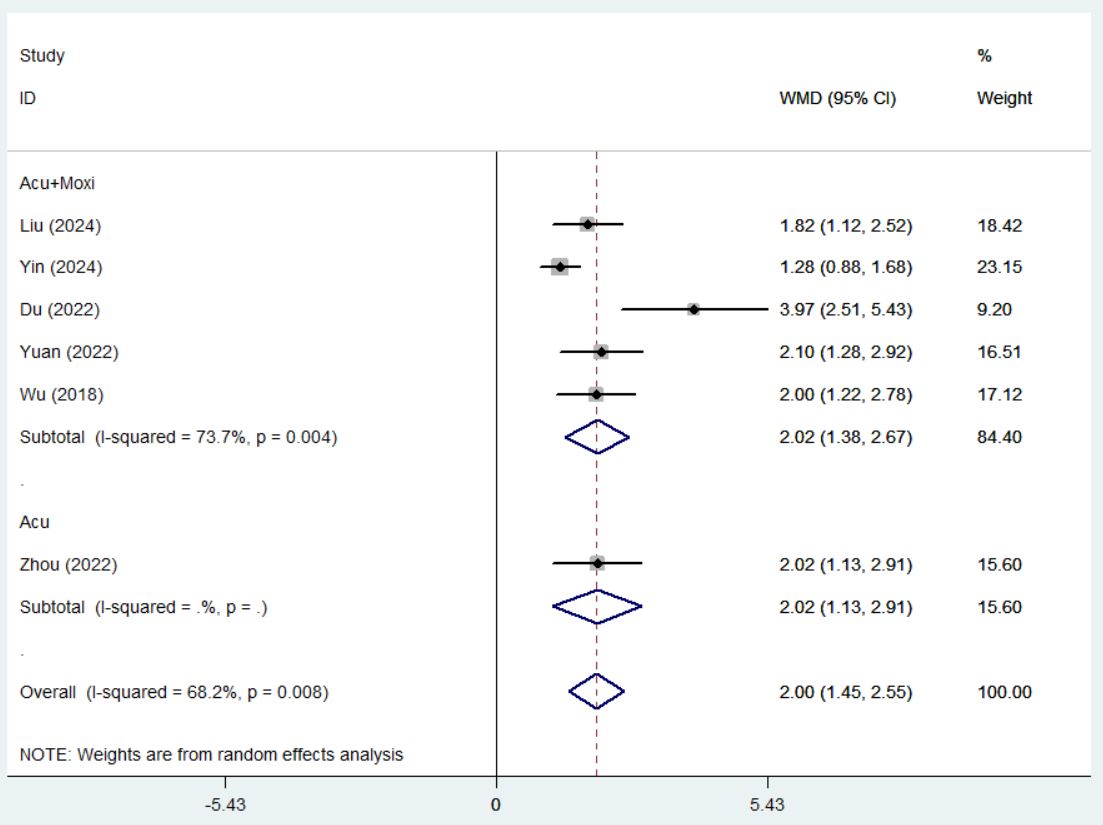


Supplementary FIGURE S1 The forest plot of AFC.


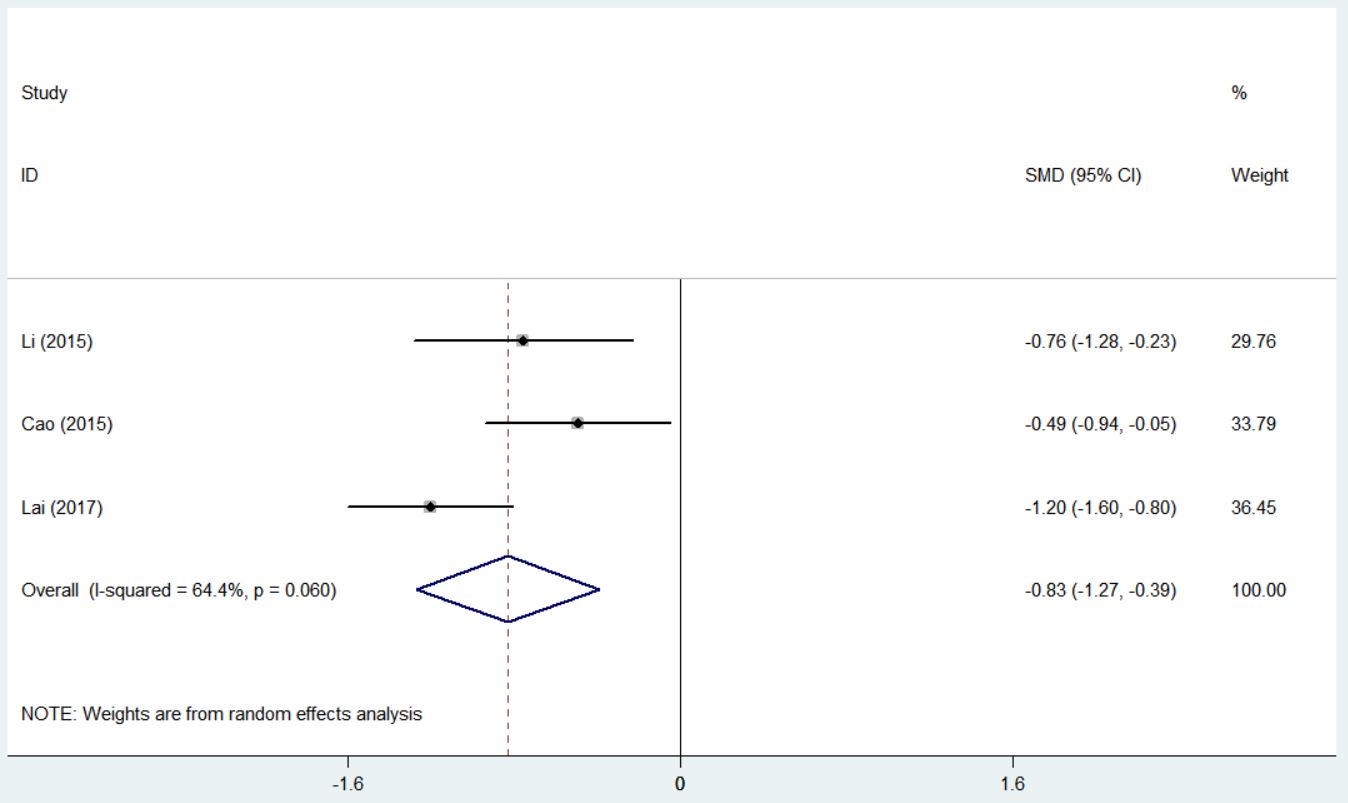


Supplementary FIGURE S2.1 The forest plot of FSH (AAT).


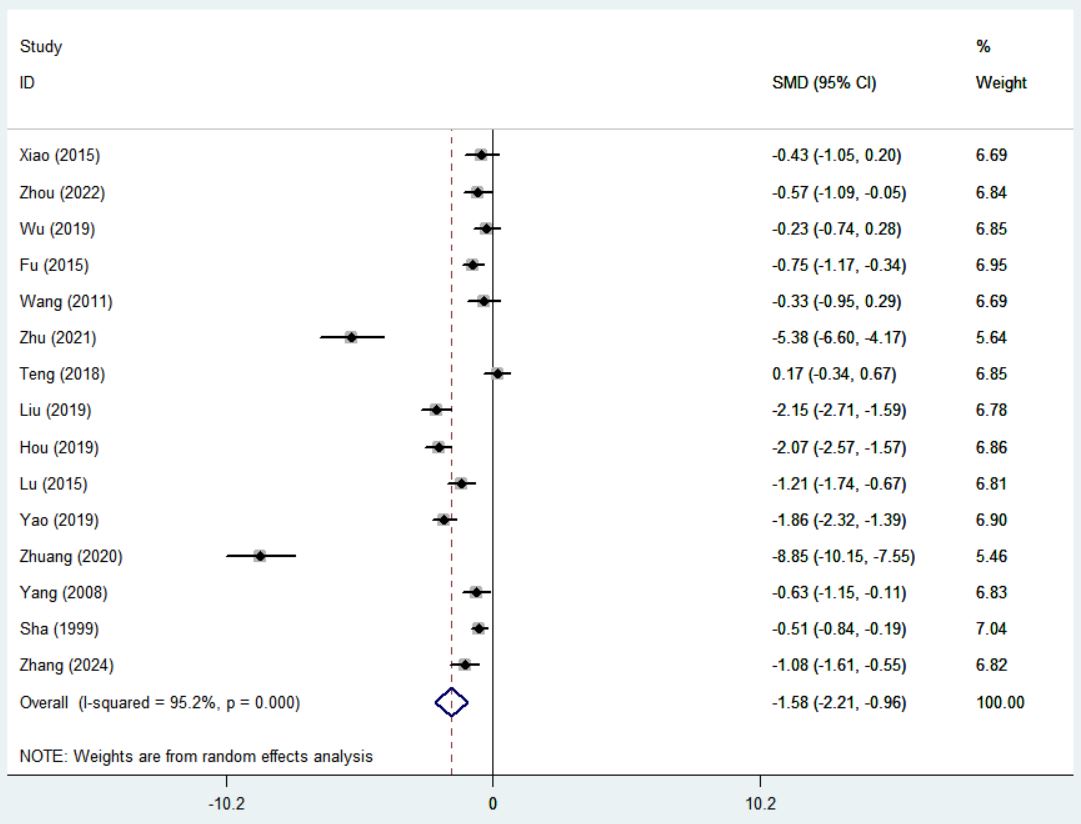


Supplementary FIGURE S2.2 The forest plot of FSH (Acu).


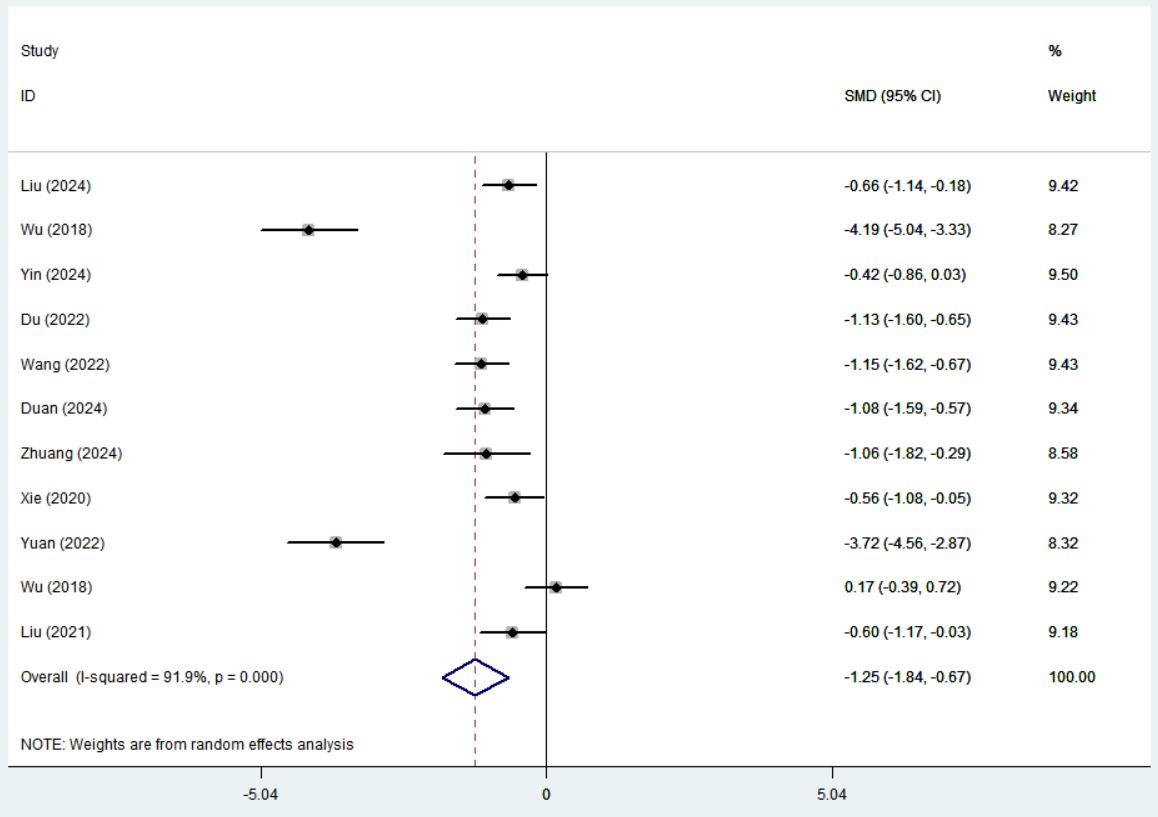


Supplementary FIGURE S2.3 The forest plot of FSH (Acu + Moxi).


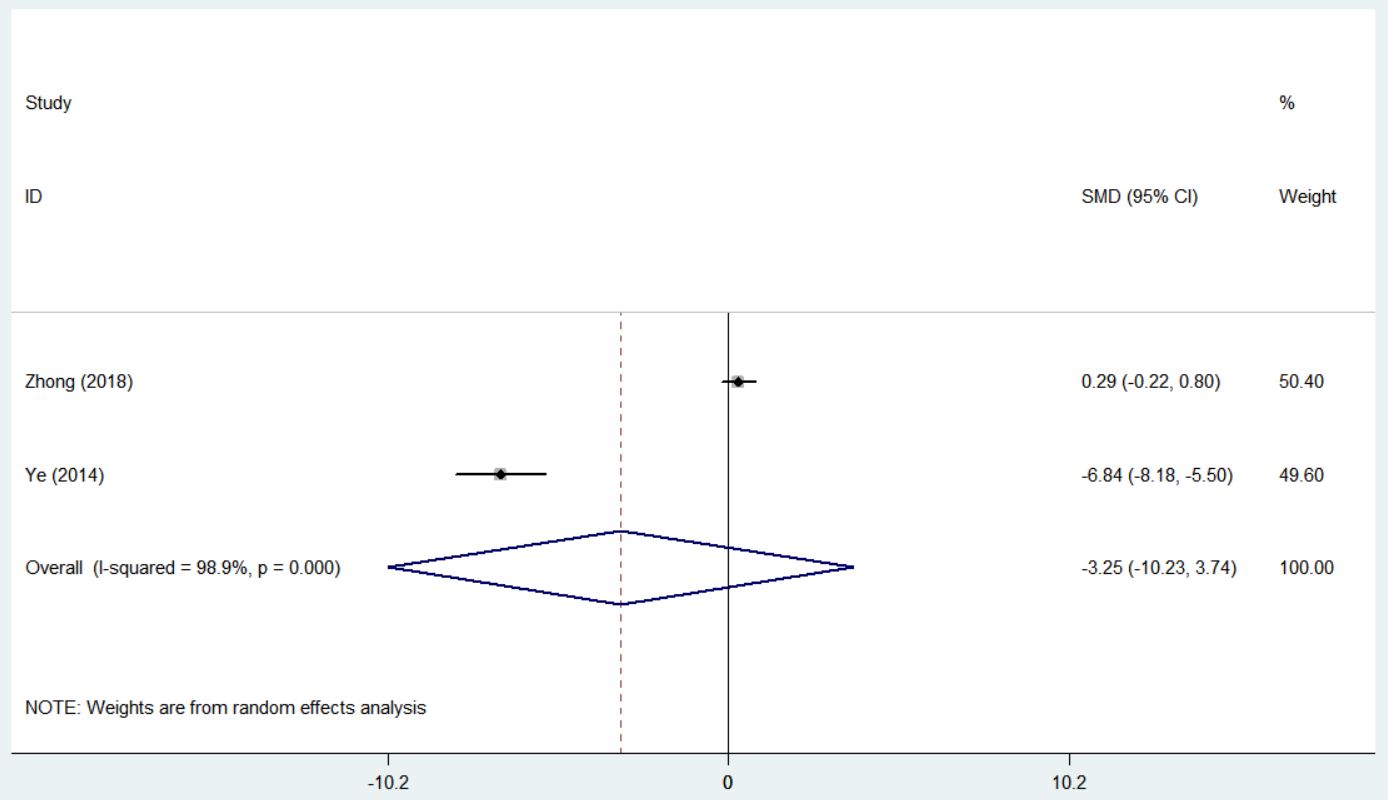


Supplementary FIGURE S2.4 The forest plot of FSH (AST).


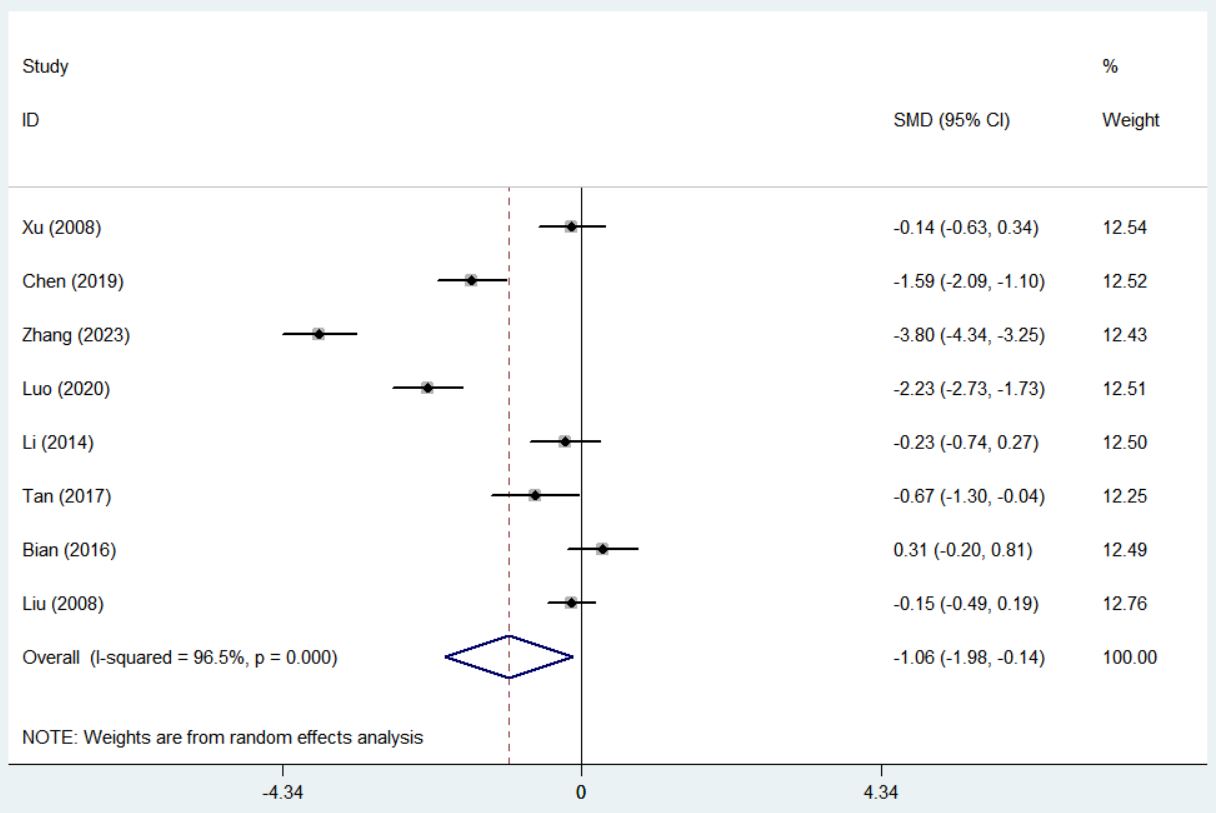


Supplementary FIGURE S2.5 The forest plot of FSH (CIAA).


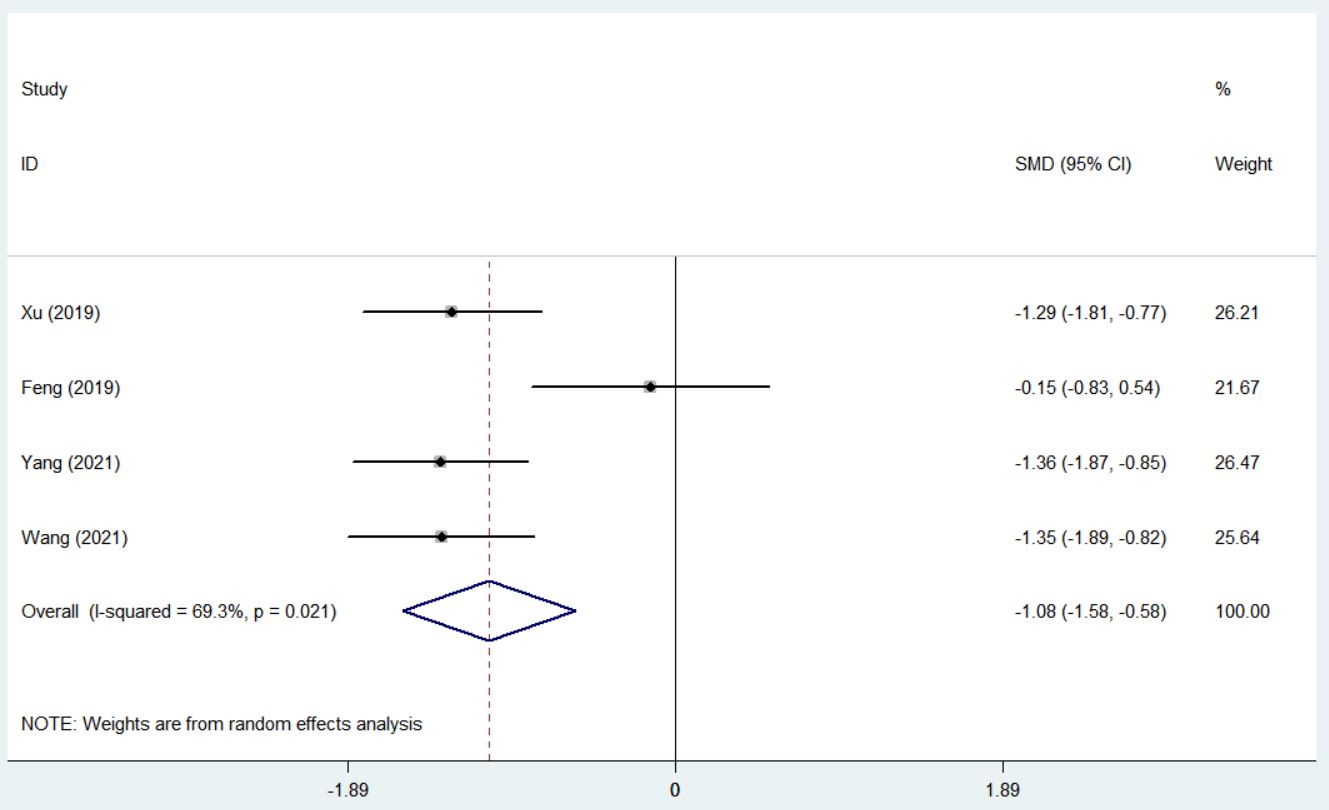


Supplementary FIGURE S2.6 The forest plot of FSH (Moxi).


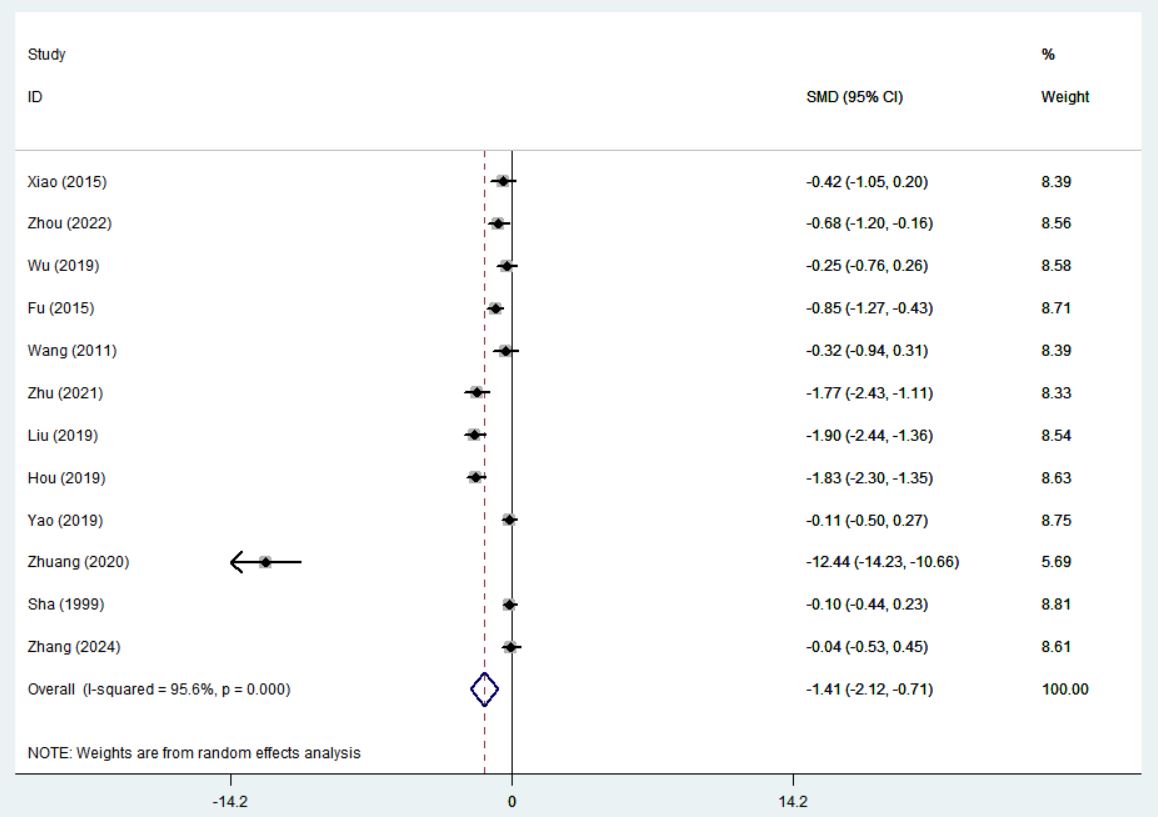


Supplementary FIGURE S3.1 The forest plot of LH (Acu).


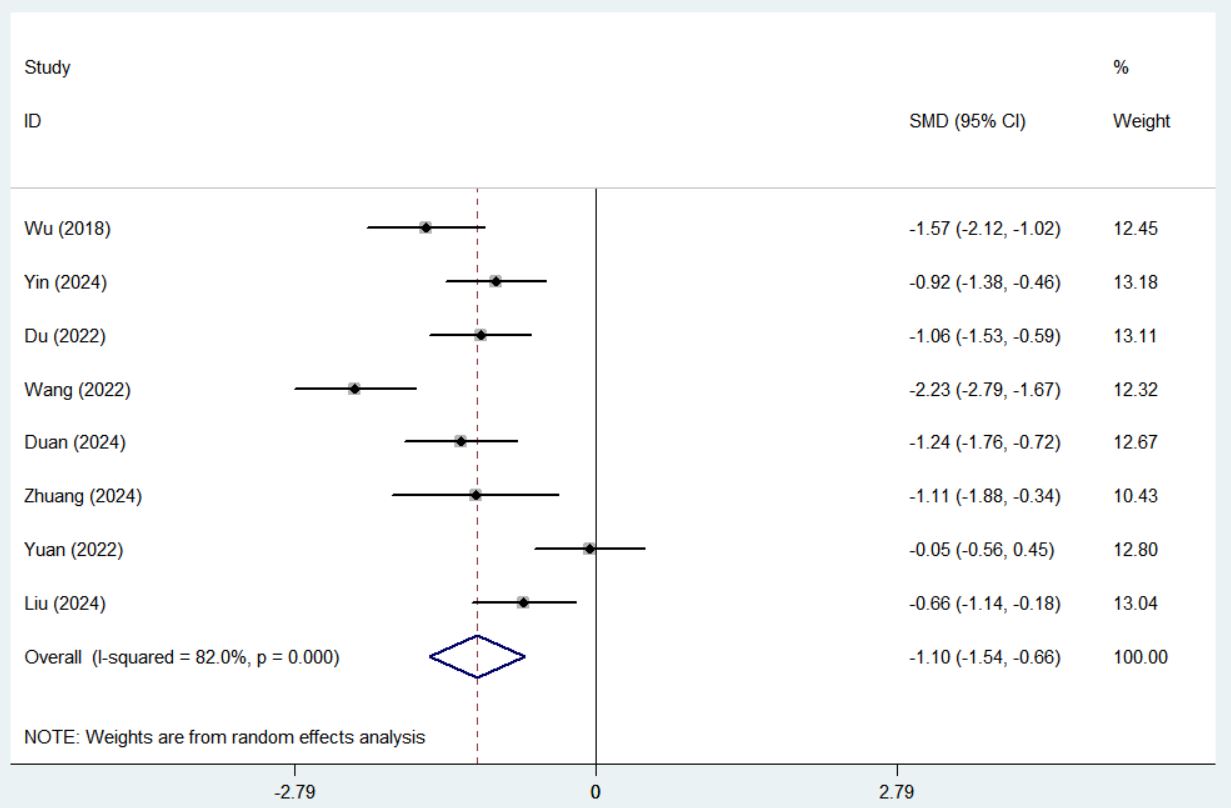


Supplementary FIGURE S3.2 The forest plot of LH (Acu + Moxi).


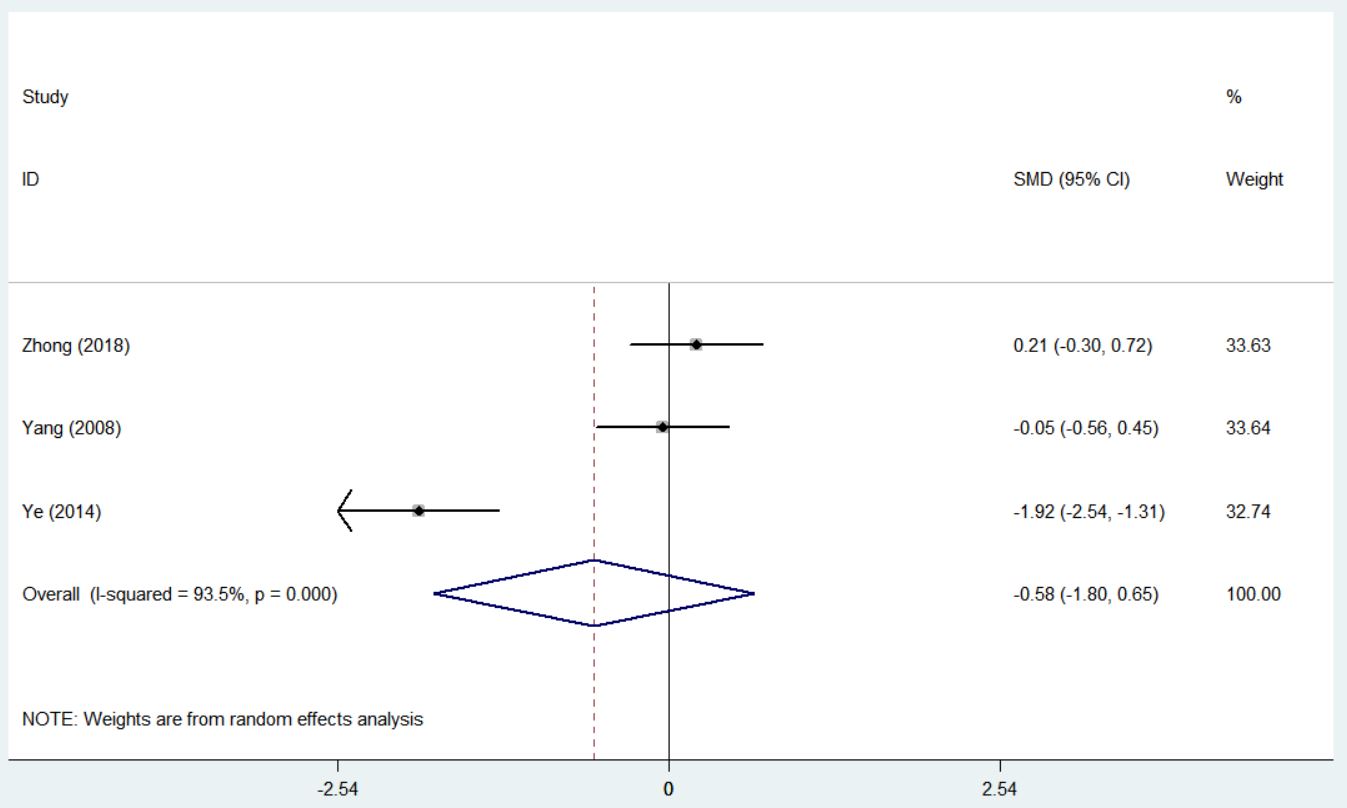


Supplementary FIGURE S3.3 The forest plot of LH (AST).


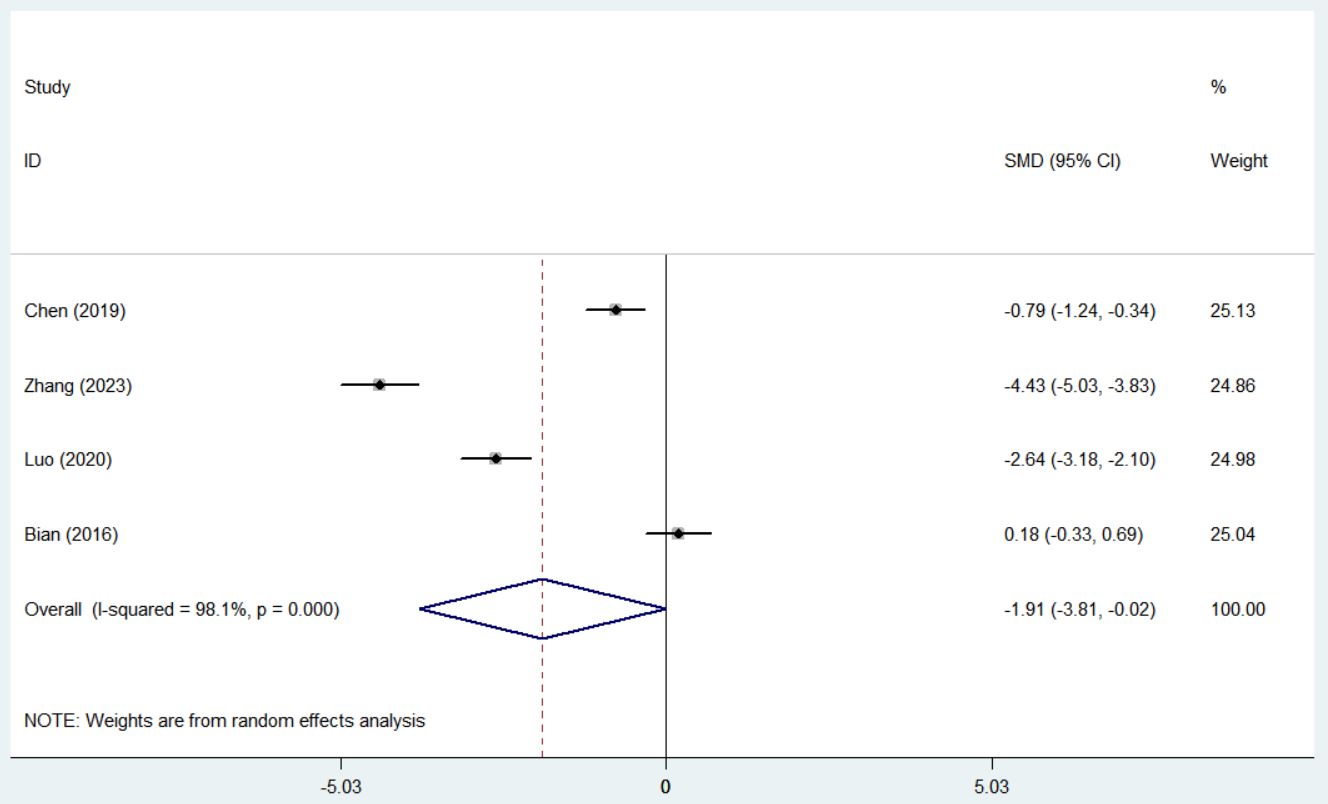


Supplementary FIGURE S3.4 The forest plot of LH (CIAA).


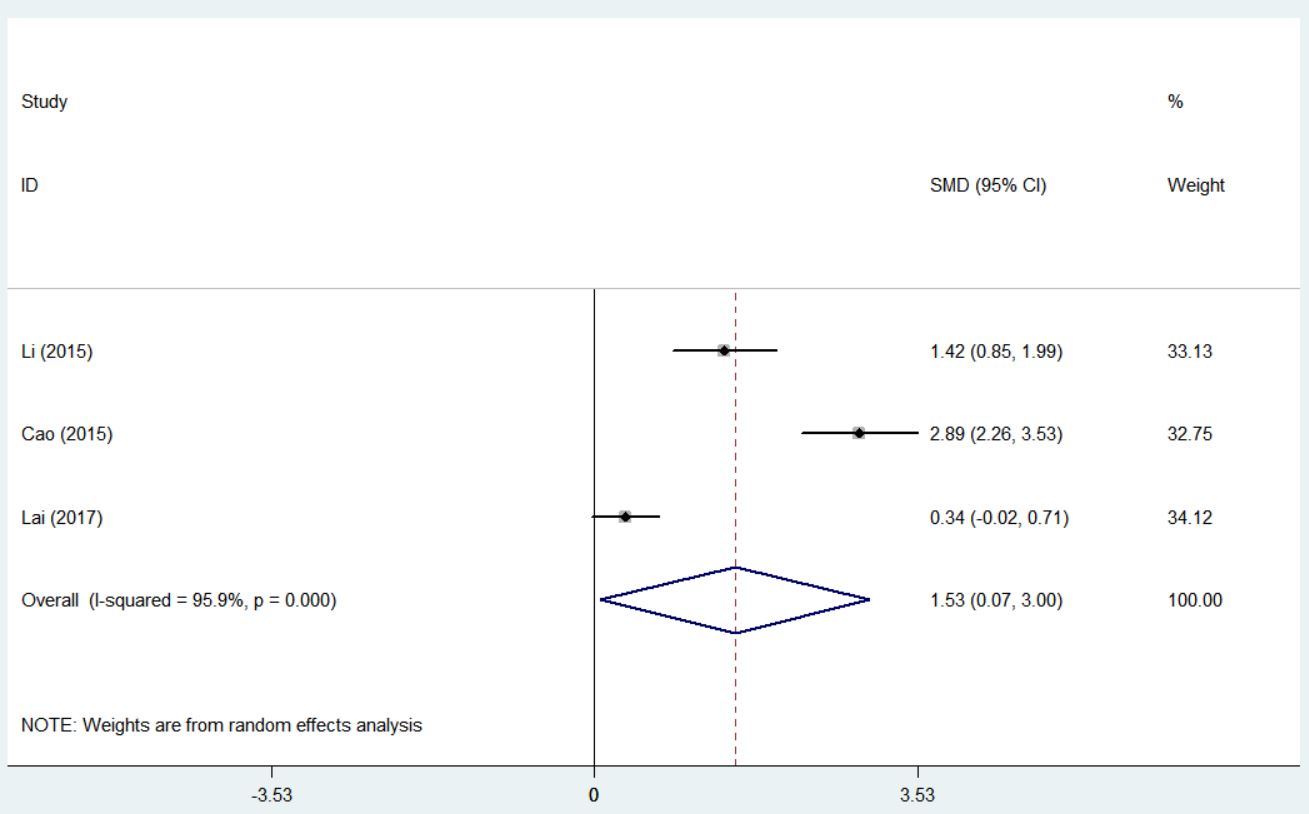


Supplementary FIGURE S4.1 The forest plot of E_2_ (AAT).


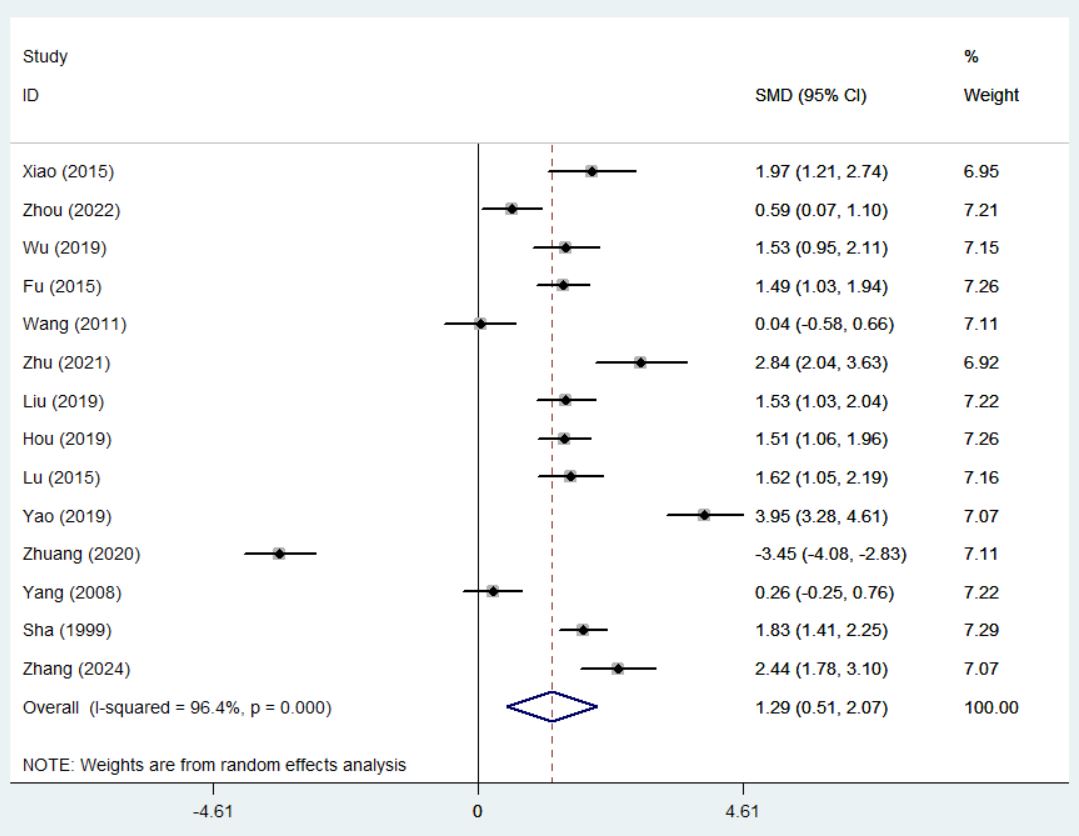


Supplementary FIGURE S4.2 The forest plot of E_2_ (Acu).


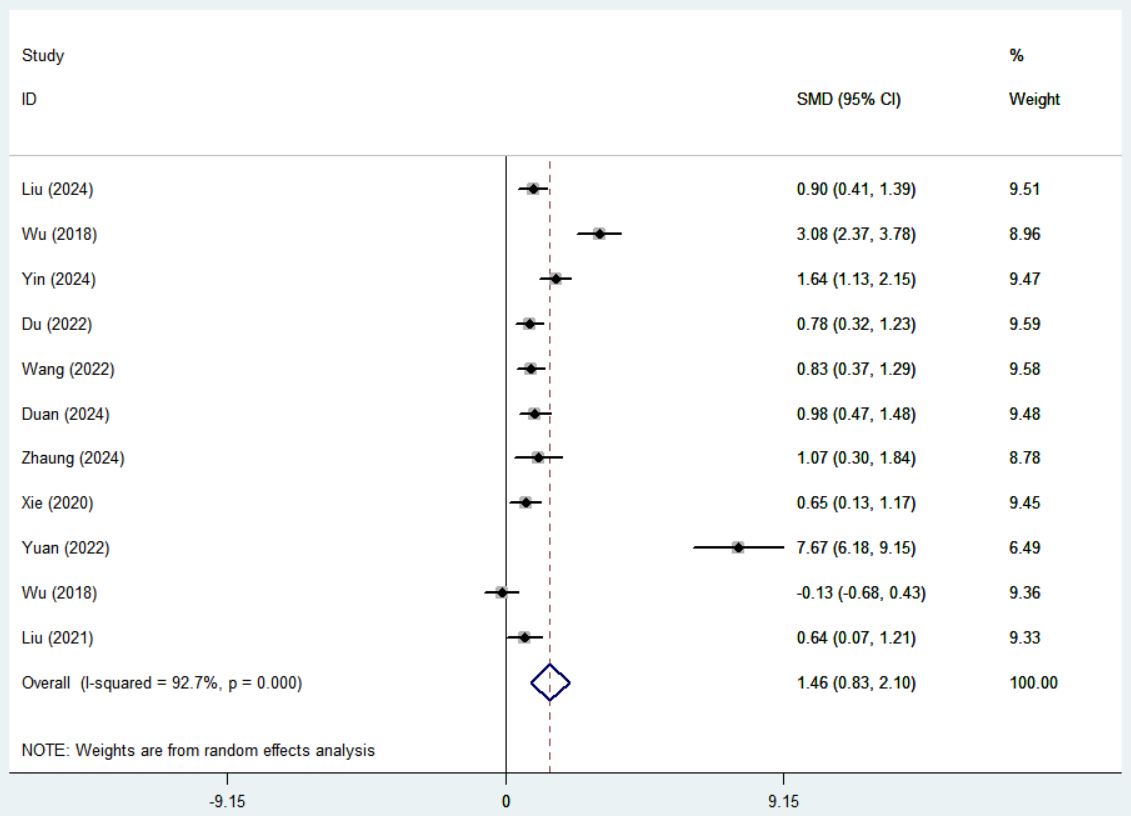


Supplementary FIGURE S4.3 The forest plot of E_2_ (Acu + Moxi).


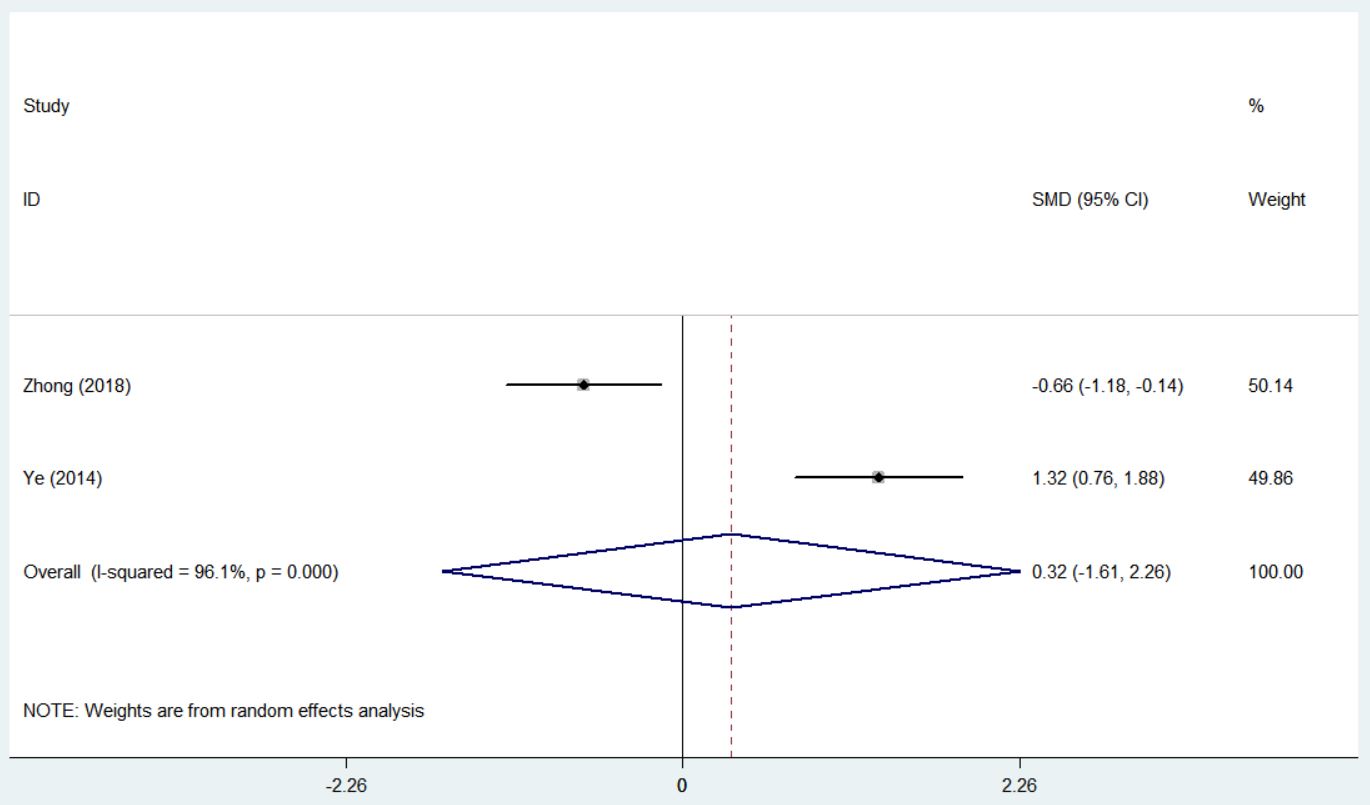


Supplementary FIGURE S4.4 The forest plot of E_2_ (AST).


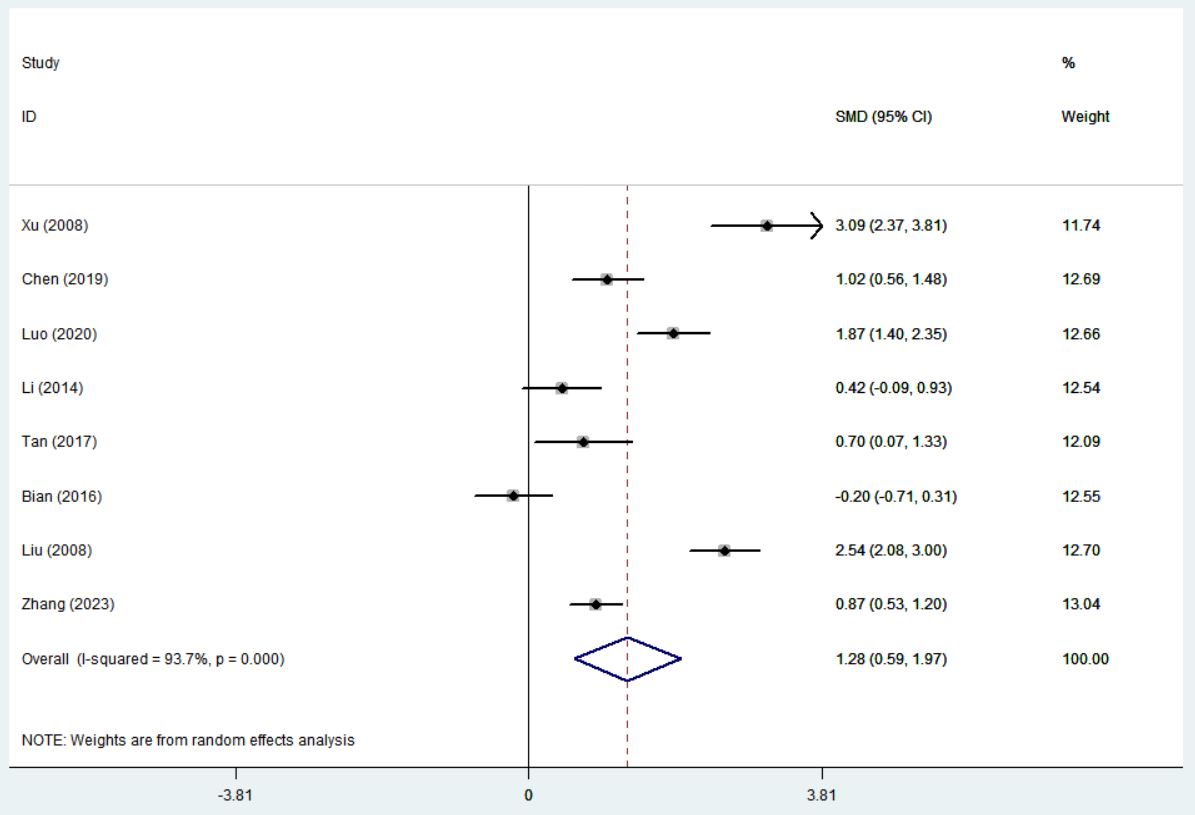


Supplementary FIGURE S4.5 The forest plot of E_2_ (CIAA).


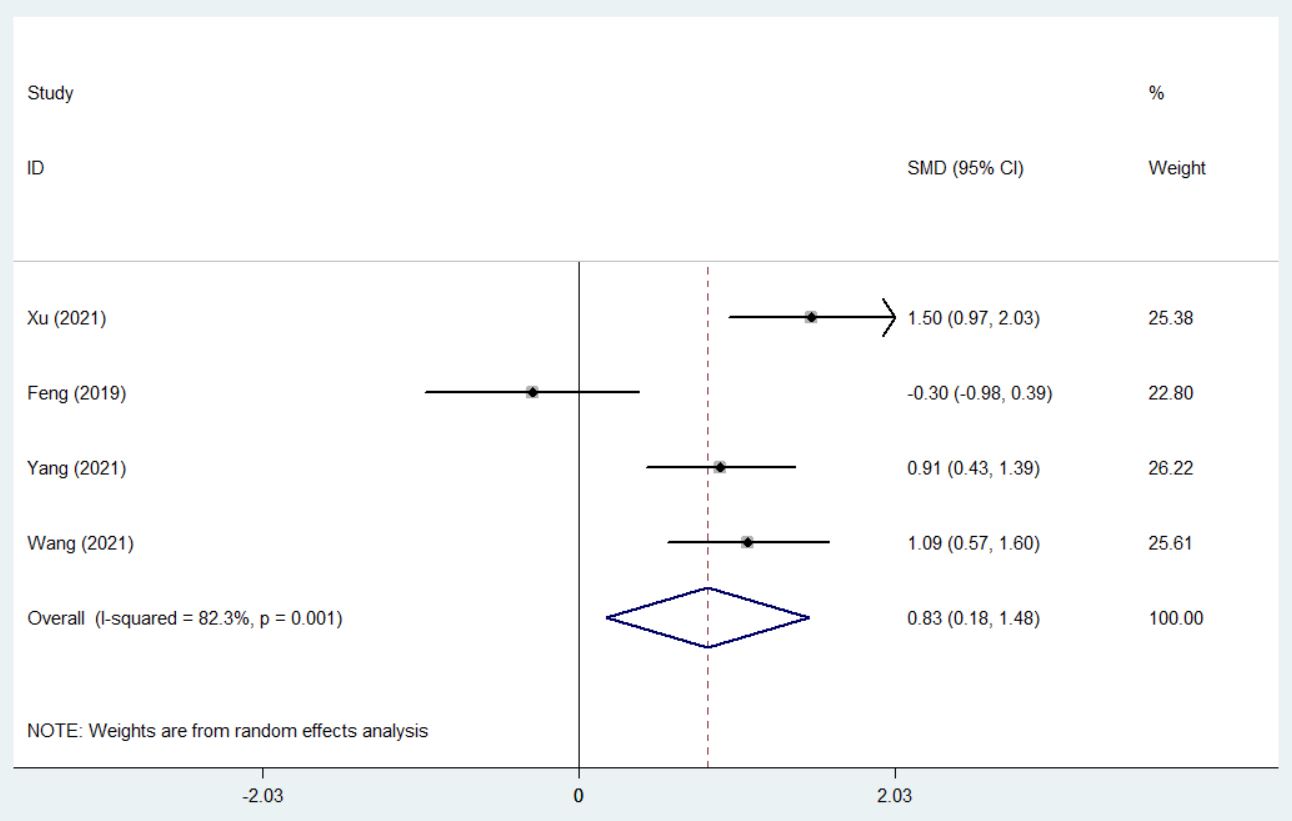


Supplementary FIGURE S4.6 The forest plot of E_2_ (Moxi).


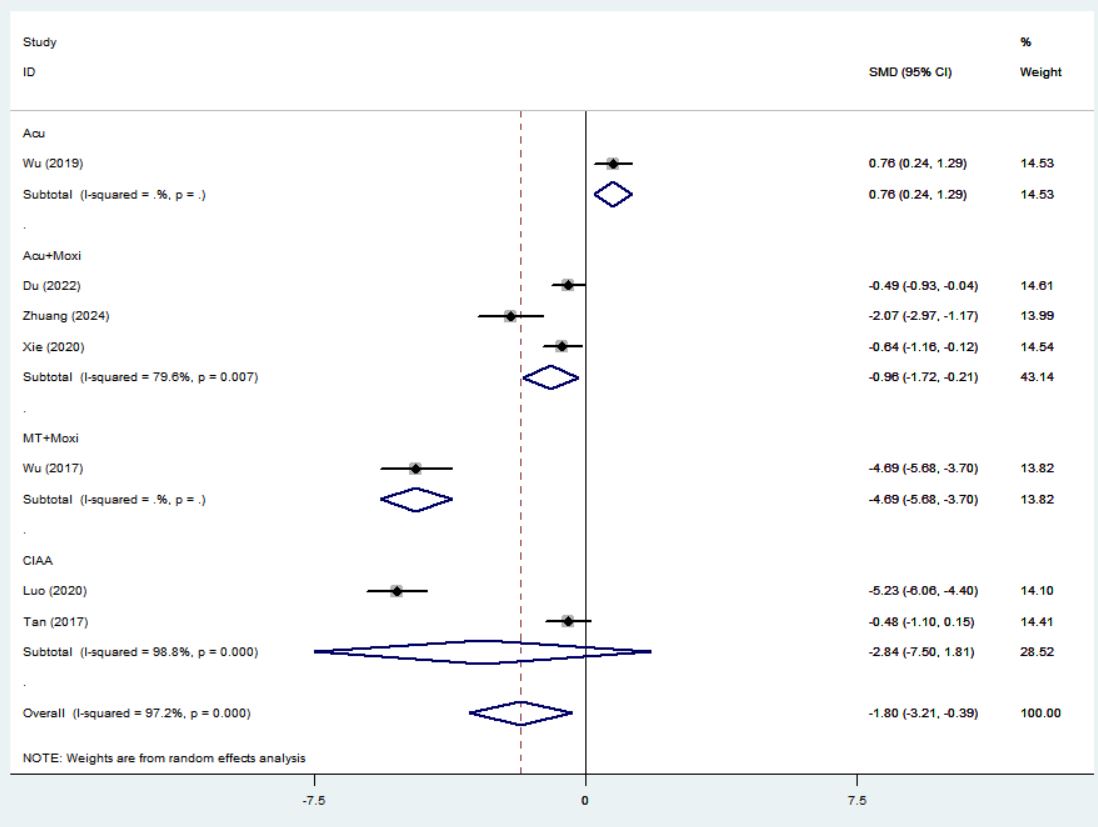


Supplementary FIGURE S5 The forest plot of Kupperman (Acu, Acu + Moxi, MT + Moxi, CIAA).


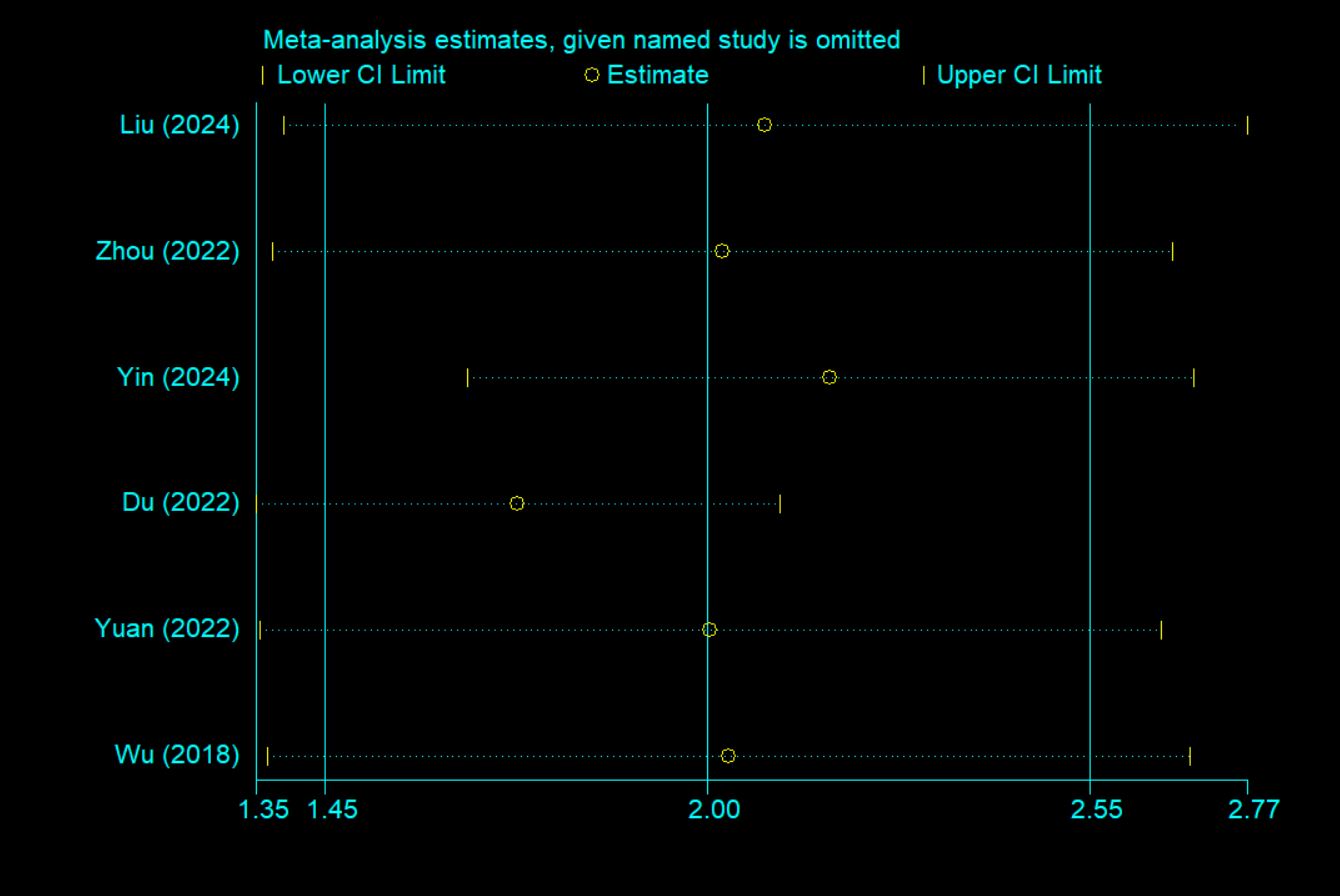


Supplementary FIGURE S6 The sensitivity analysis plot of AFC.


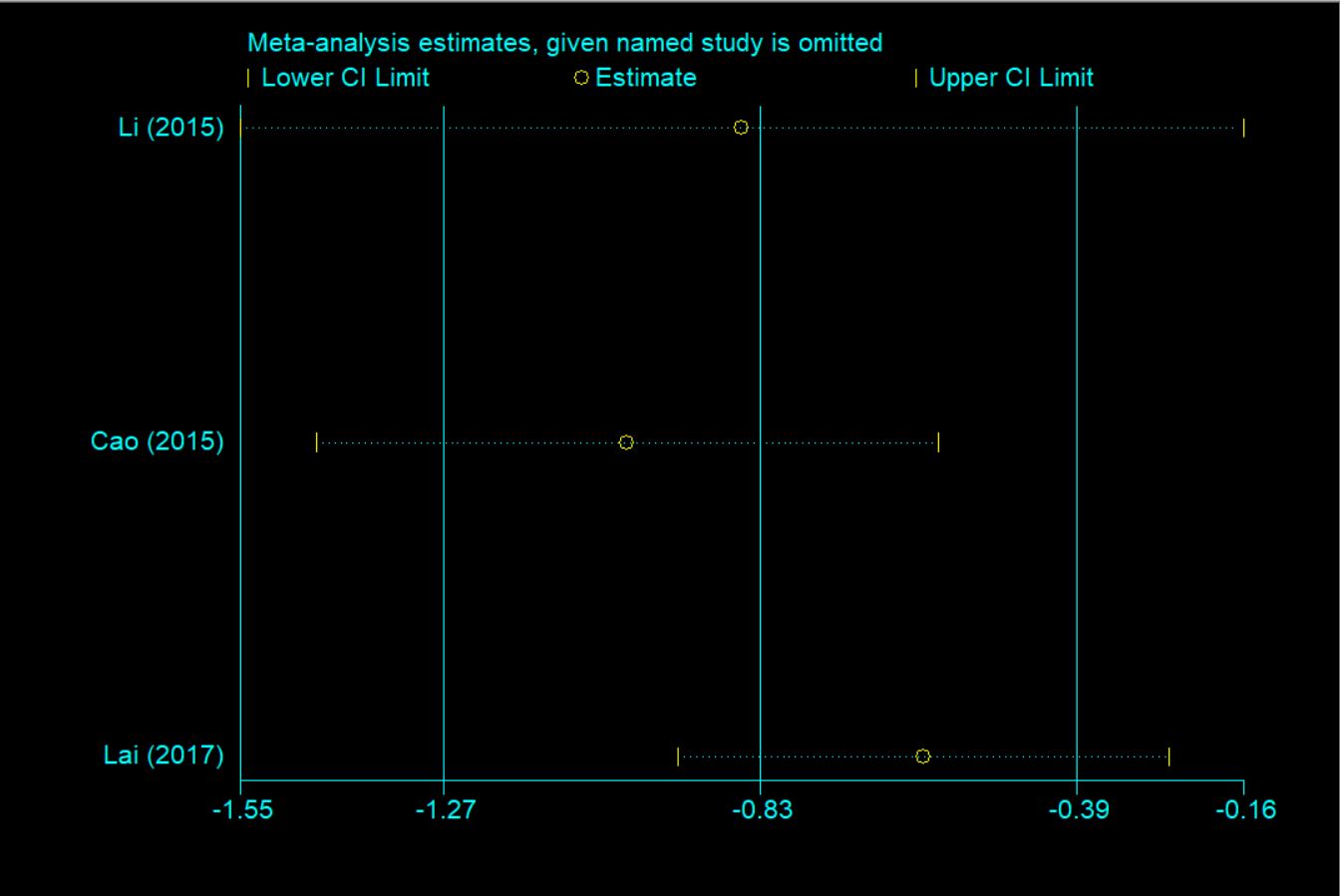


Supplementary FIGURE S7.1 The sensitivity analysis plot of FSH (AAT).


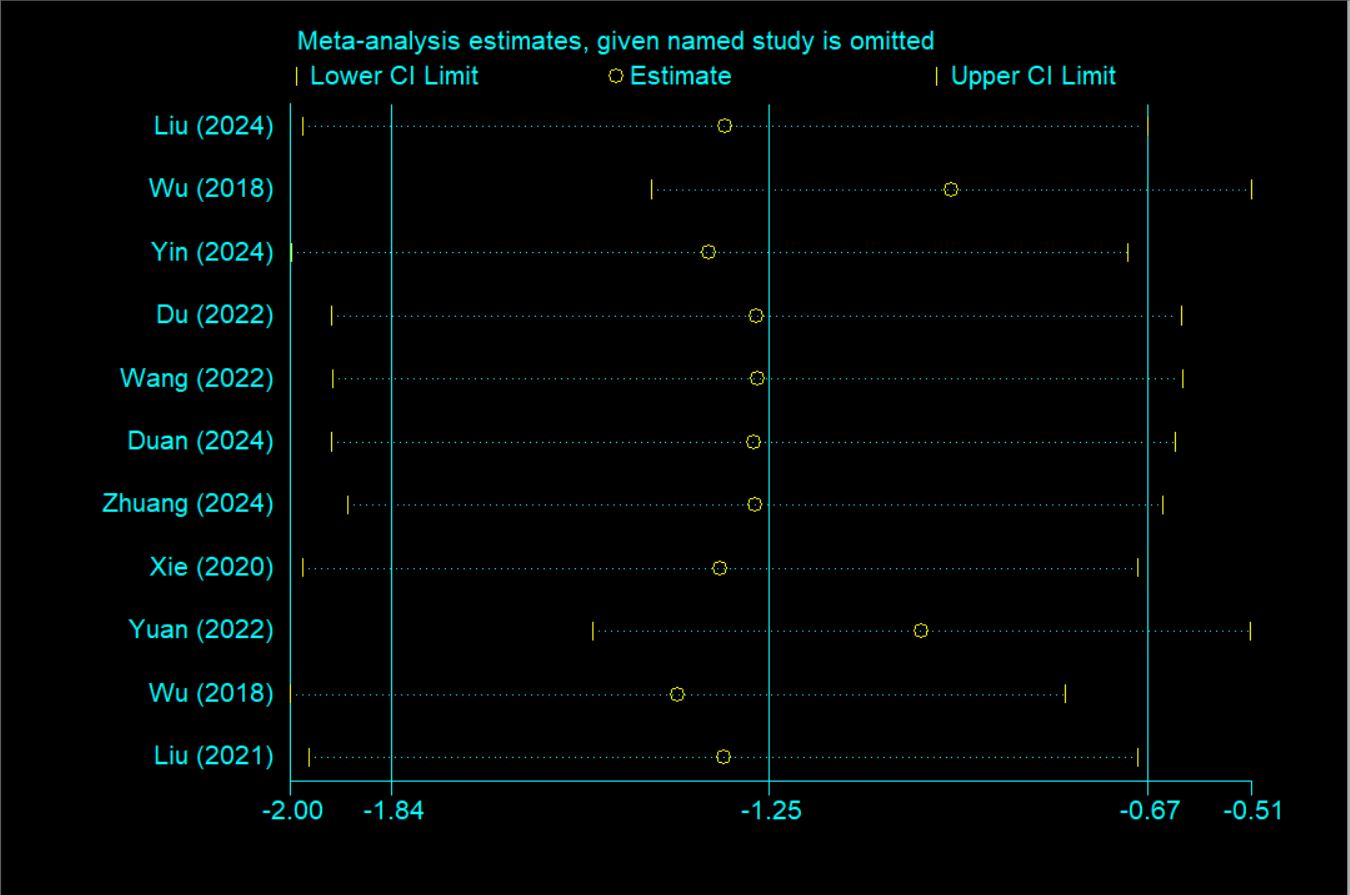


Supplementary FIGURE S7.2 The sensitivity analysis plot of FSH (Acu).


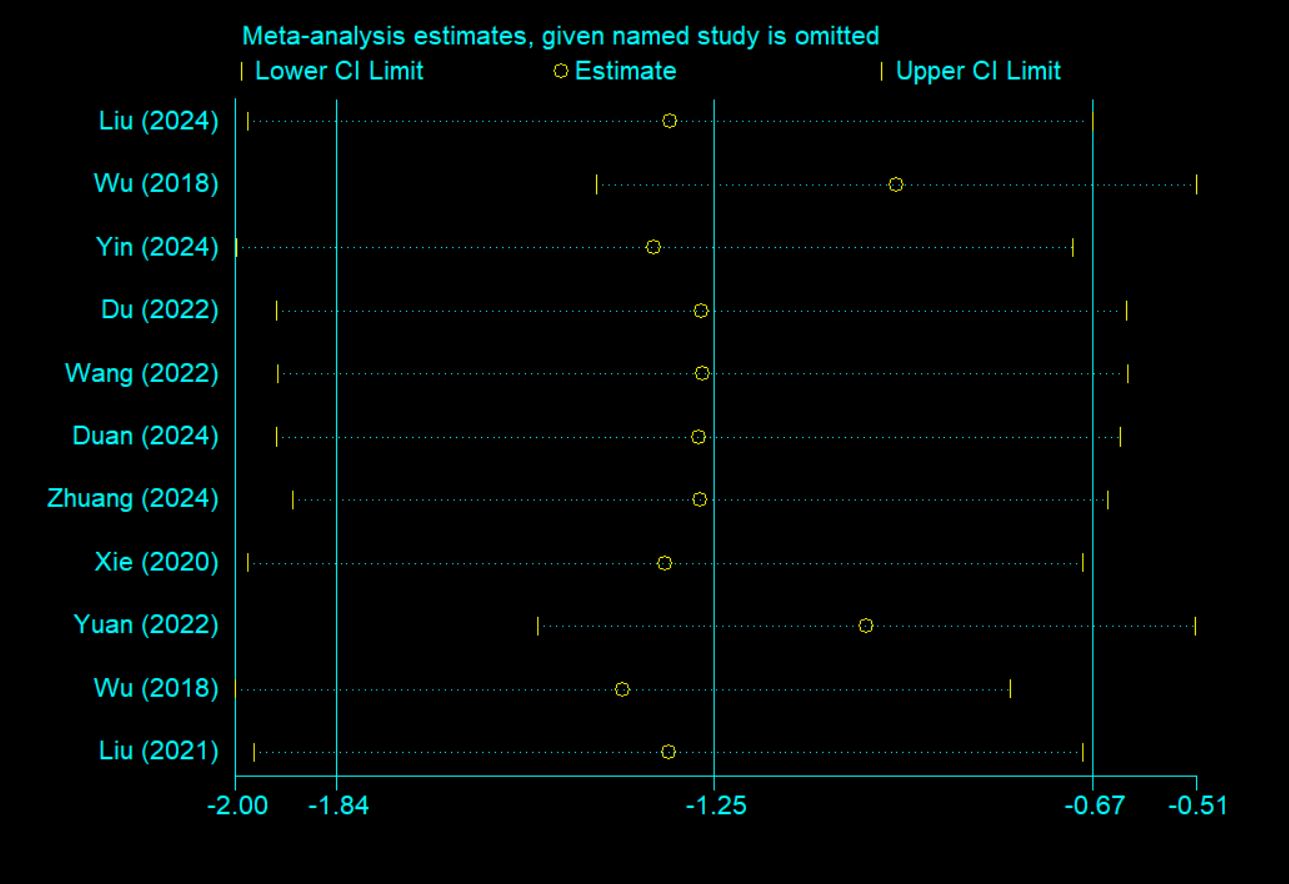


Supplementary FIGURE S7.3 The sensitivity analysis plot of FSH (Acu + Moxi).


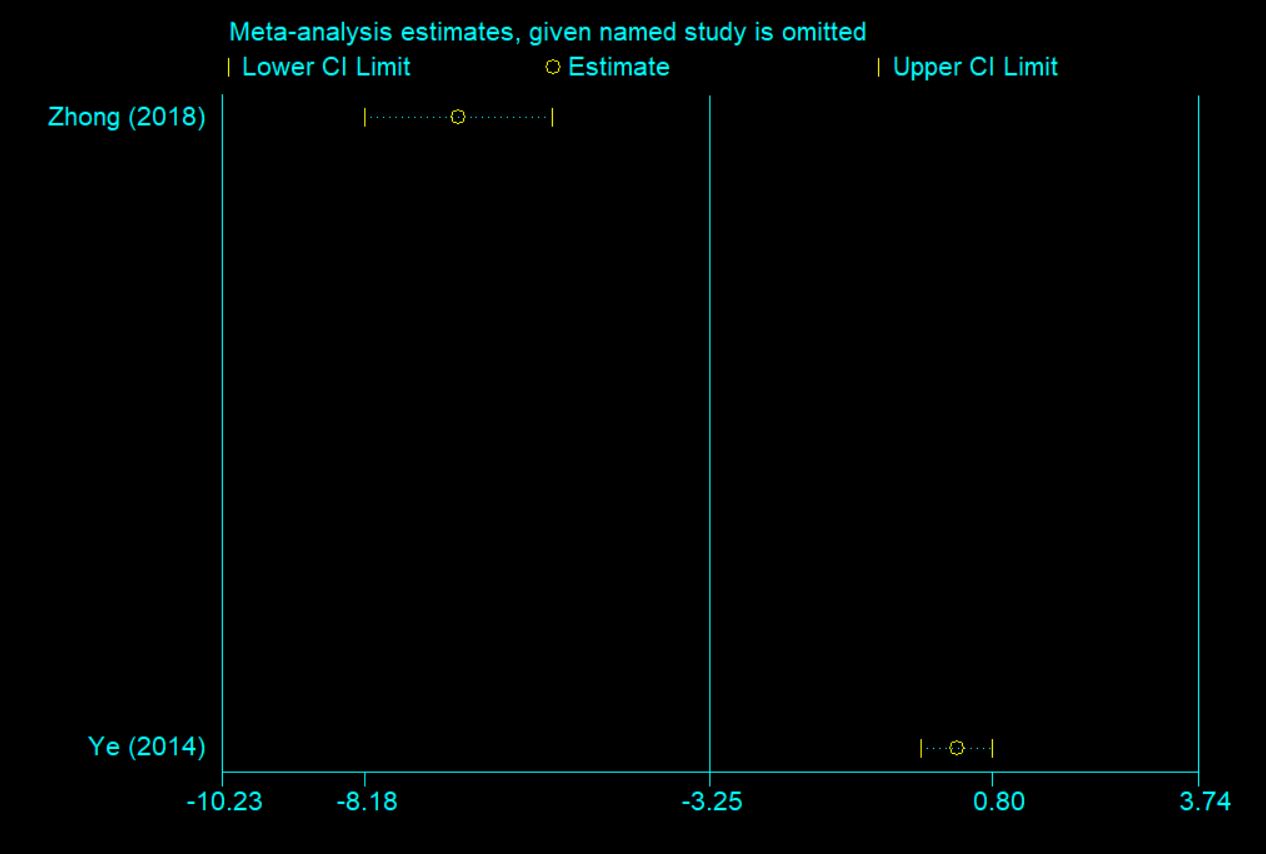


Supplementary FIGURE S7.4 The sensitivity analysis plot of FSH (AST).


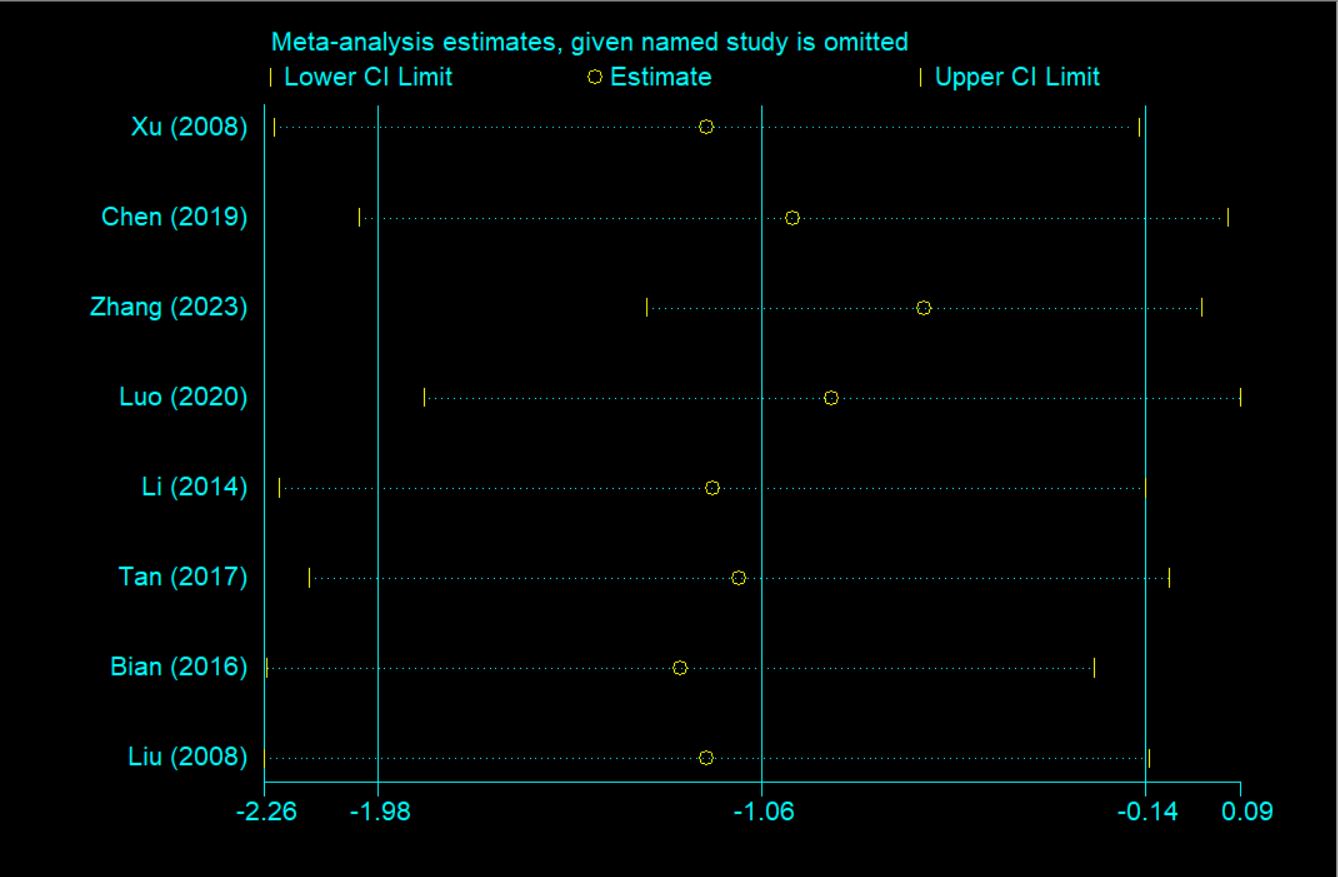


Supplementary FIGURE S7.5 The sensitivity analysis plot of FSH (CIAA).


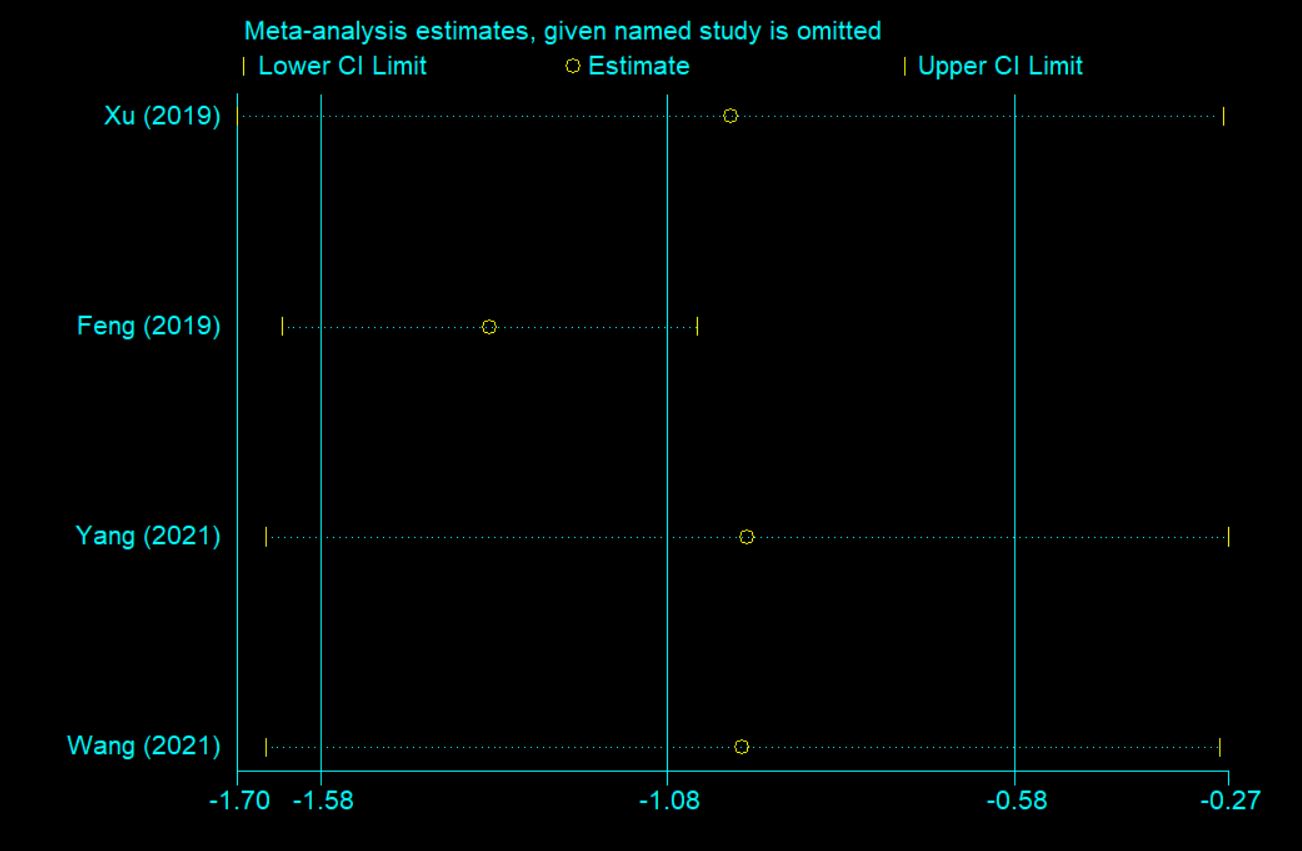


Supplementary FIGURE S7.6 The sensitivity analysis plot of FSH (Moxi).


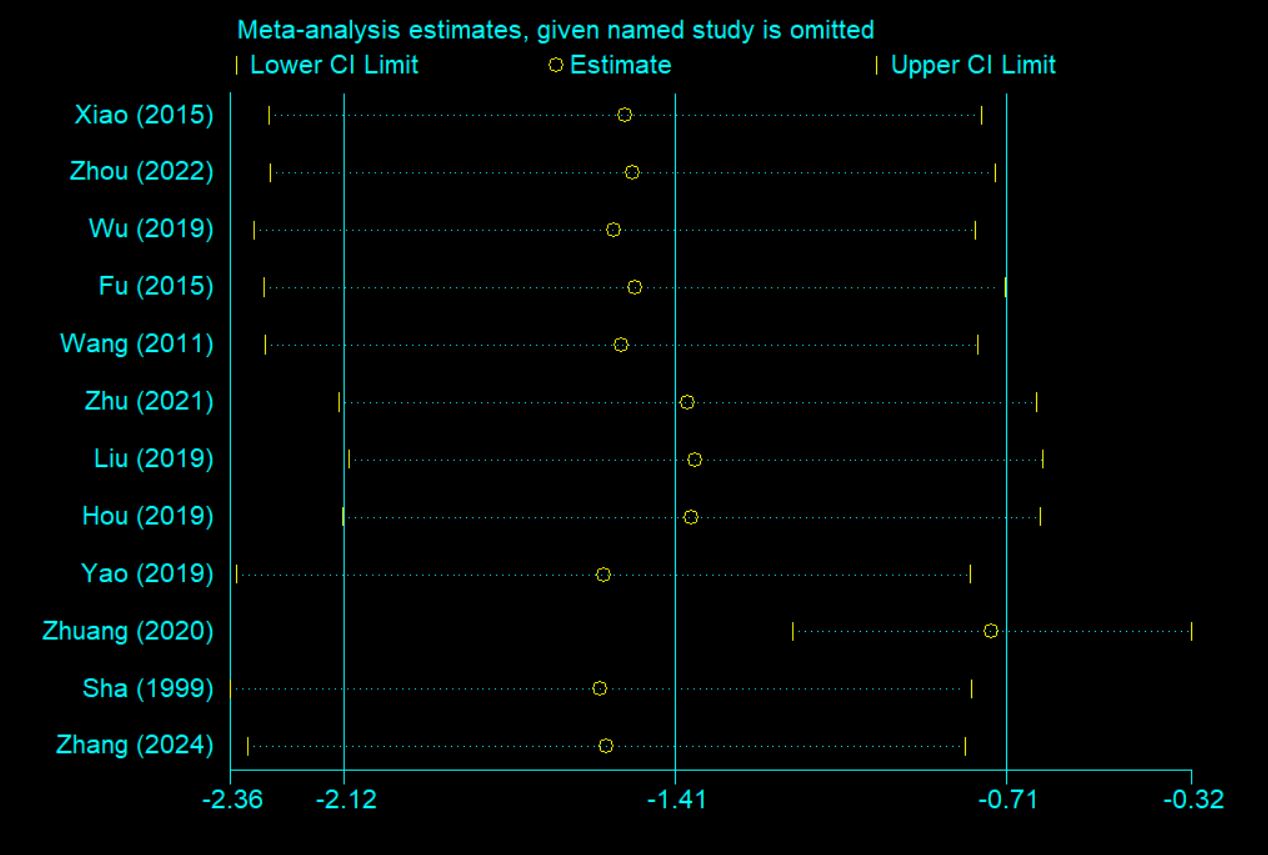


Supplementary FIGURE S8.1 The sensitivity analysis plot of LH (Acu).


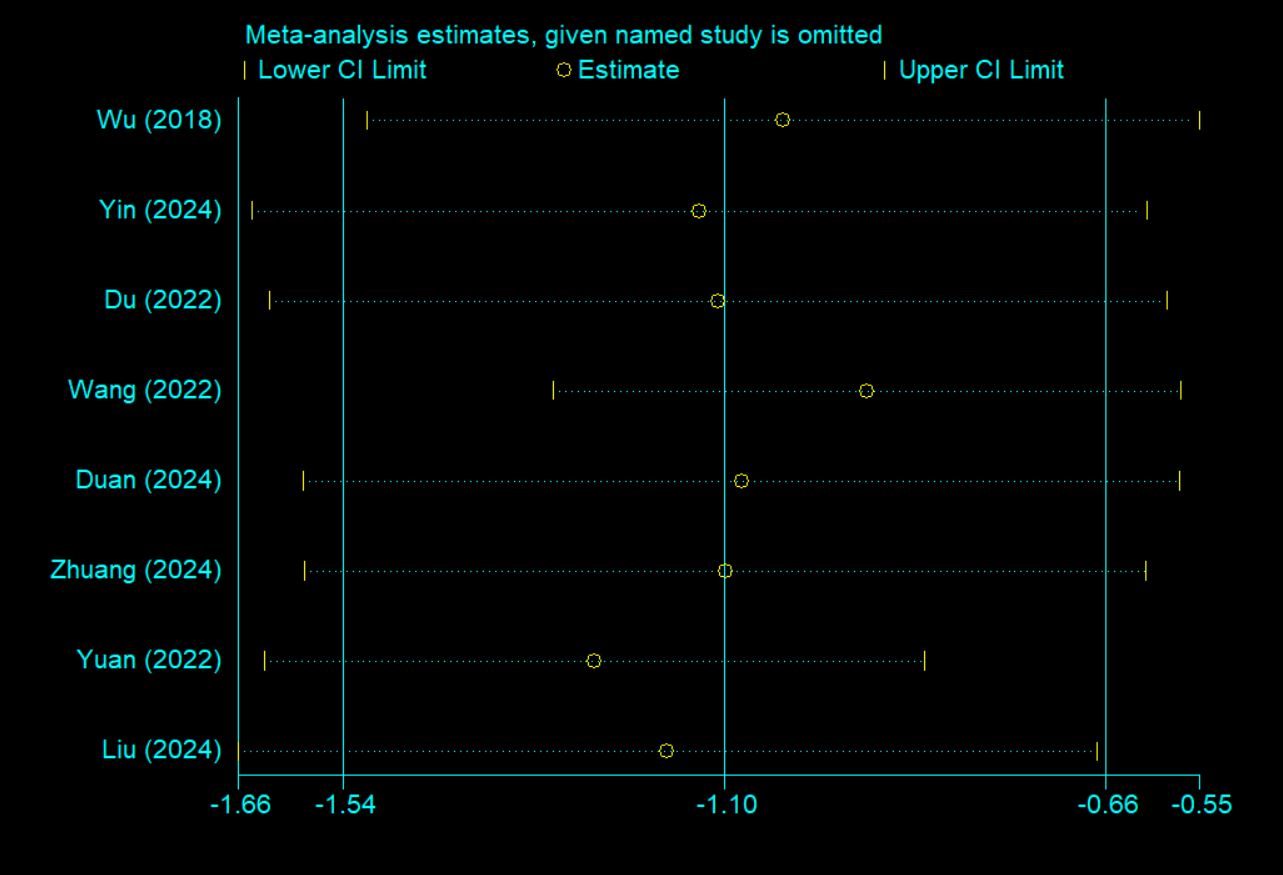


Supplementary FIGURE S8.2 The sensitivity analysis plot of LH (Acu + Moxi).


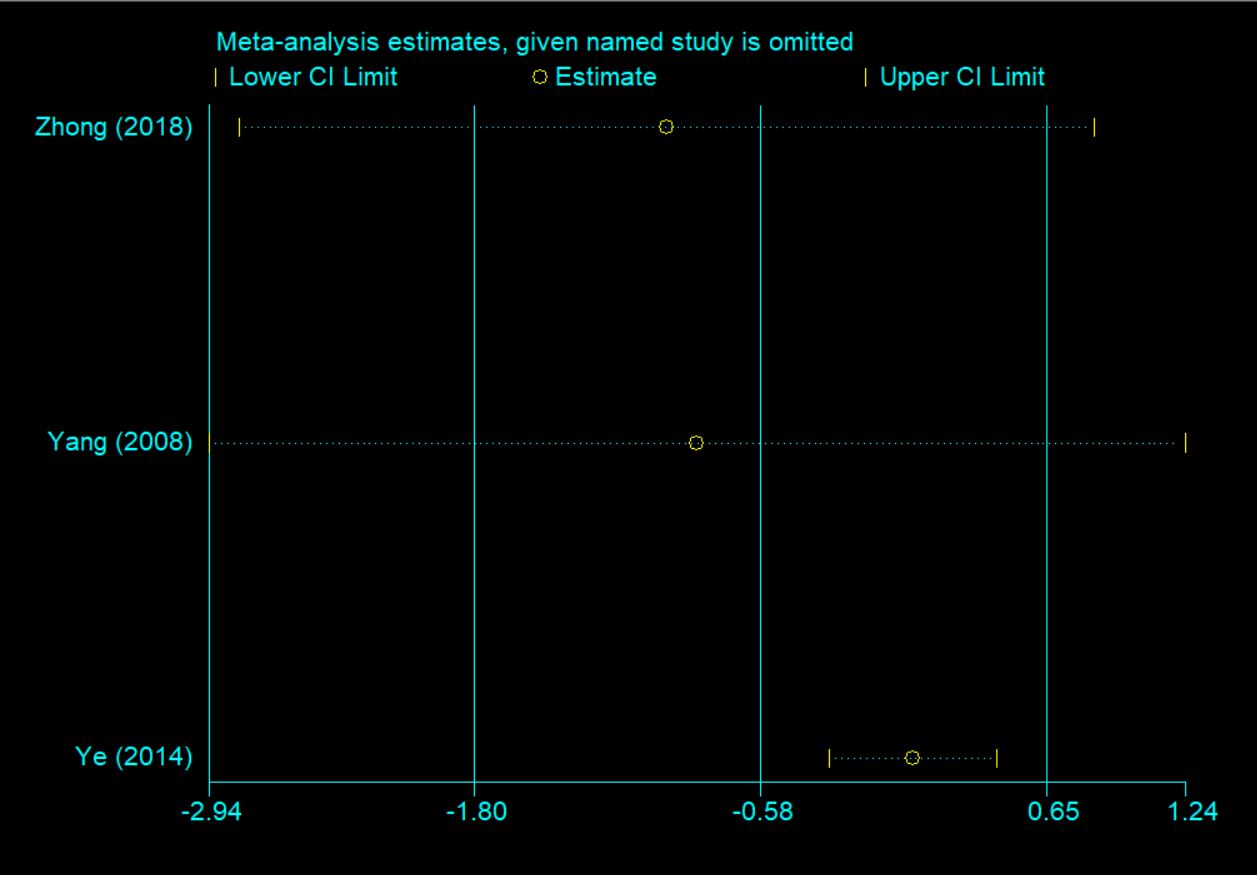


Supplementary FIGURE S8.3 The sensitivity analysis plot of LH (AST).


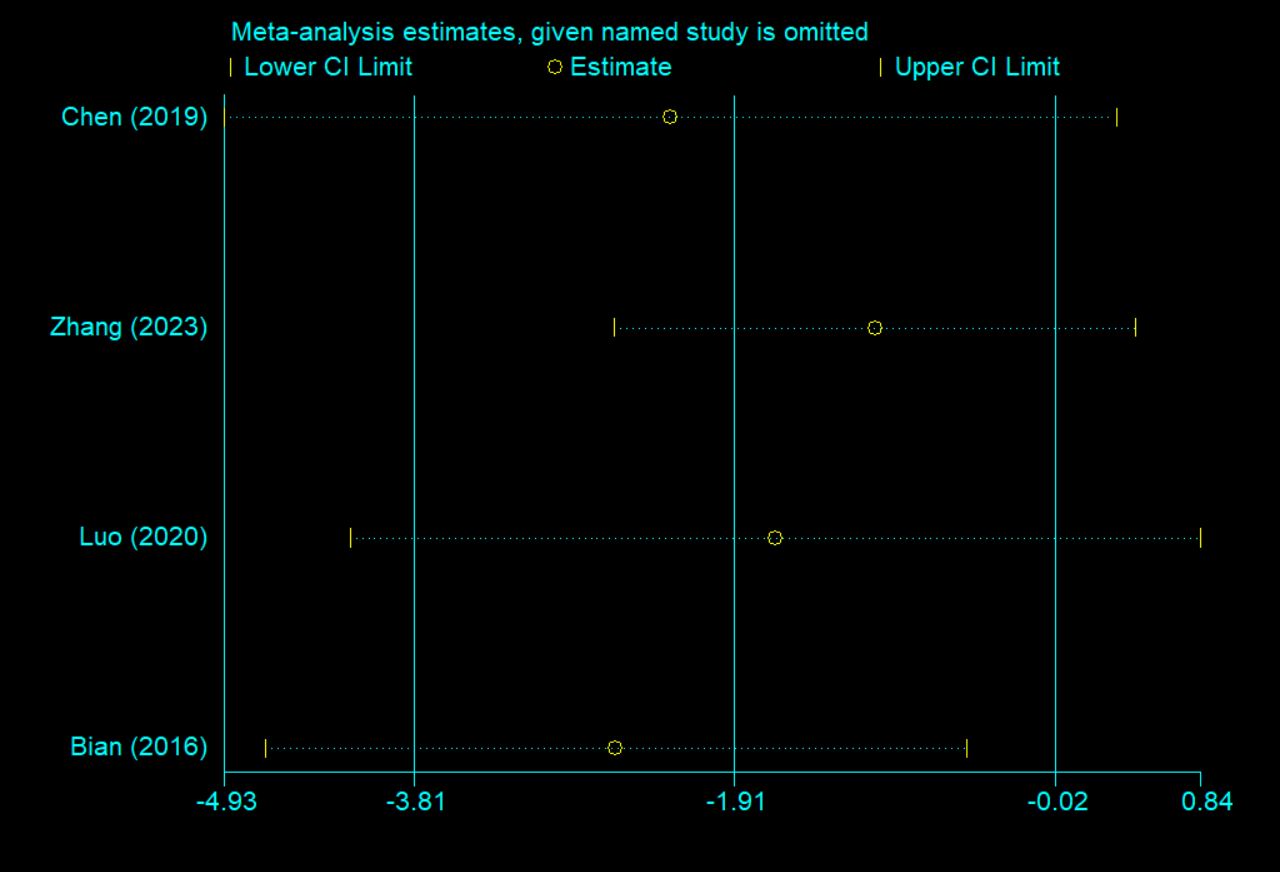


Supplementary FIGURE S8.4 The sensitivity analysis plot of LH (CIAA).


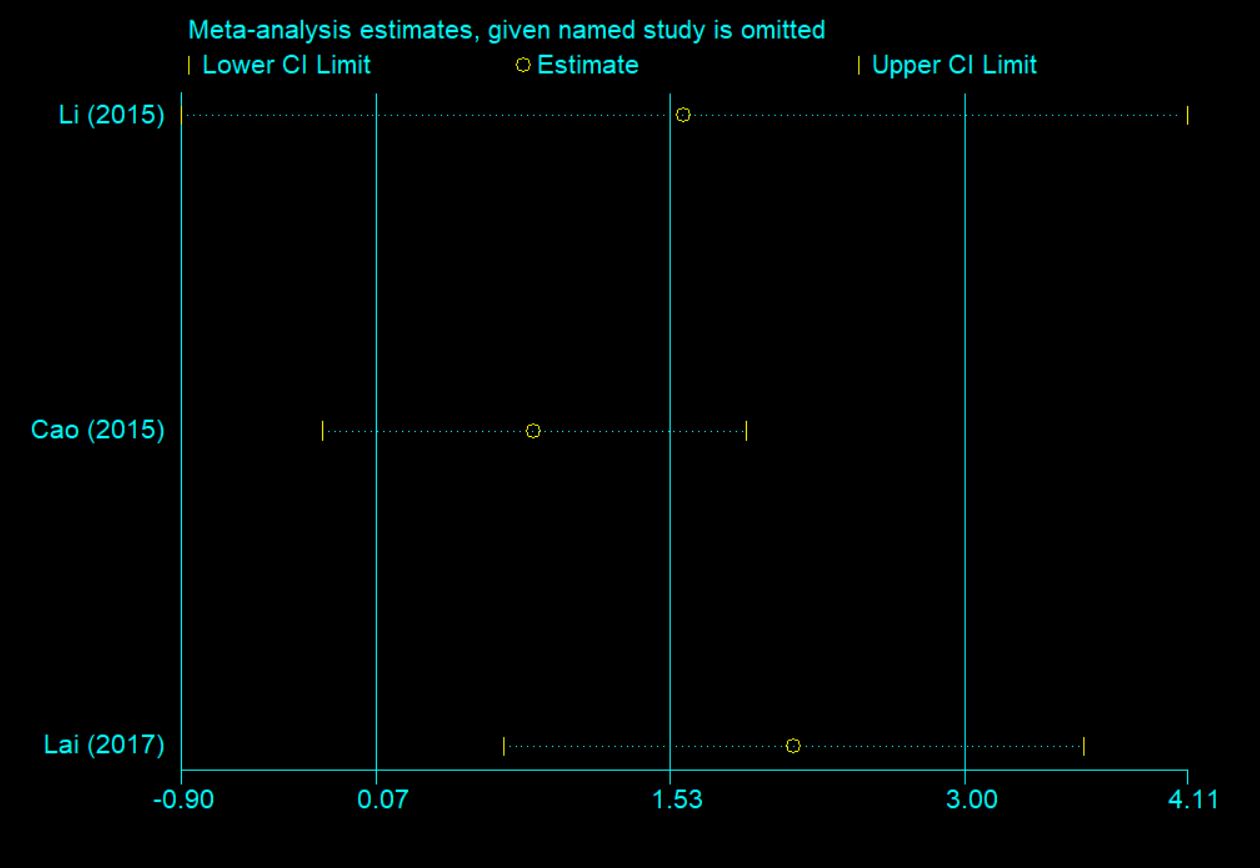


Supplementary FIGURE S9.1 The sensitivity analysis plot of E_2_ (AAT).


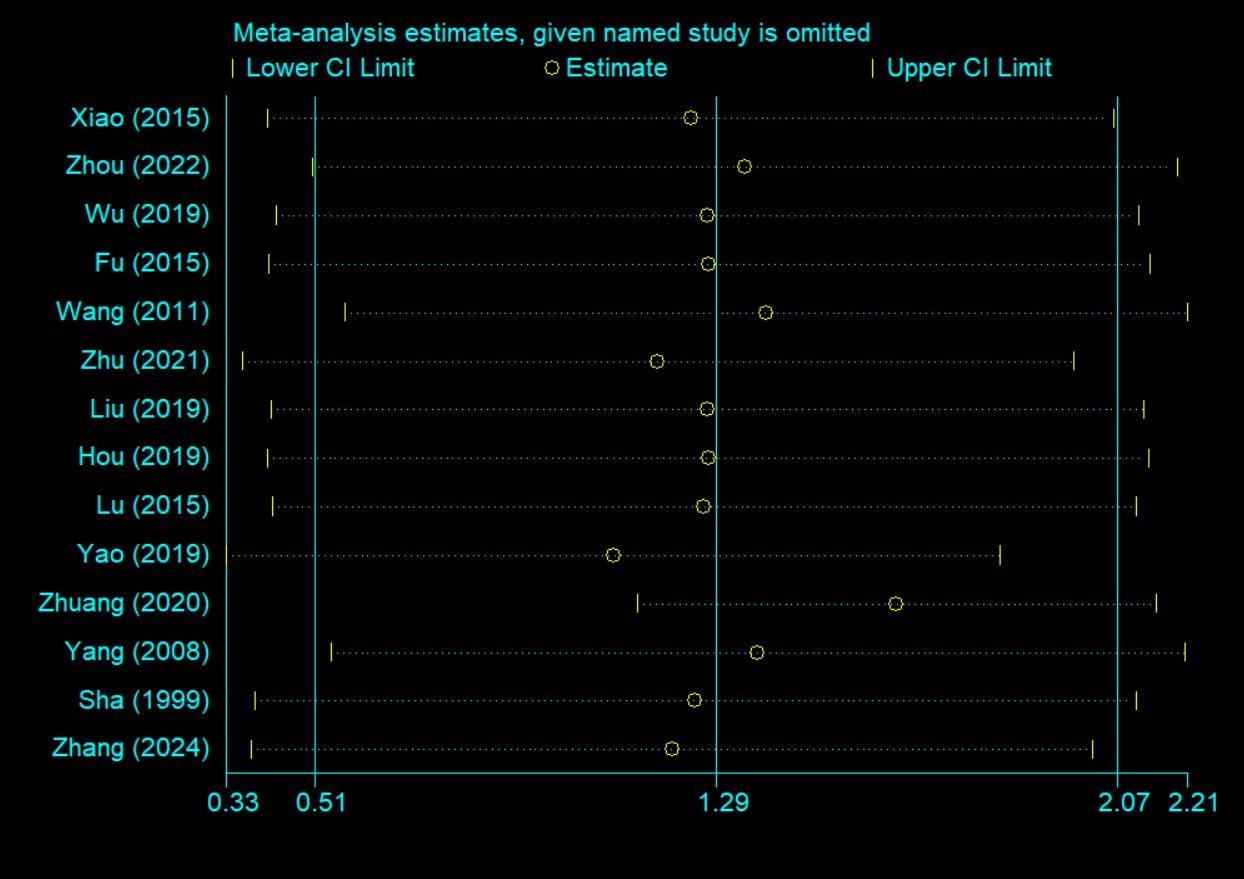


Supplementary FIGURE S9.2 The sensitivity analysis plot of E_2_ (Acu).


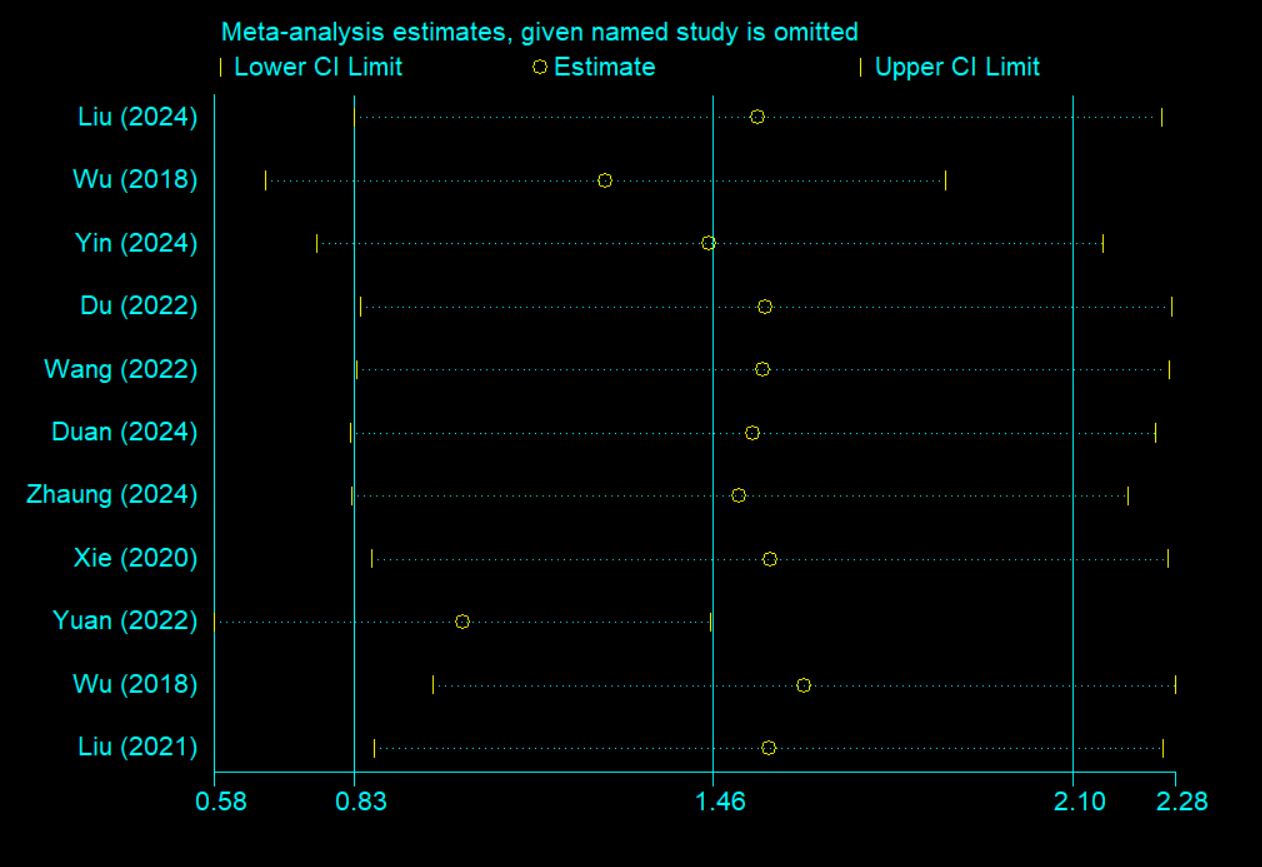


Supplementary FIGURE S9.3 The sensitivity analysis plot of E_2_ (Acu + Moxi).


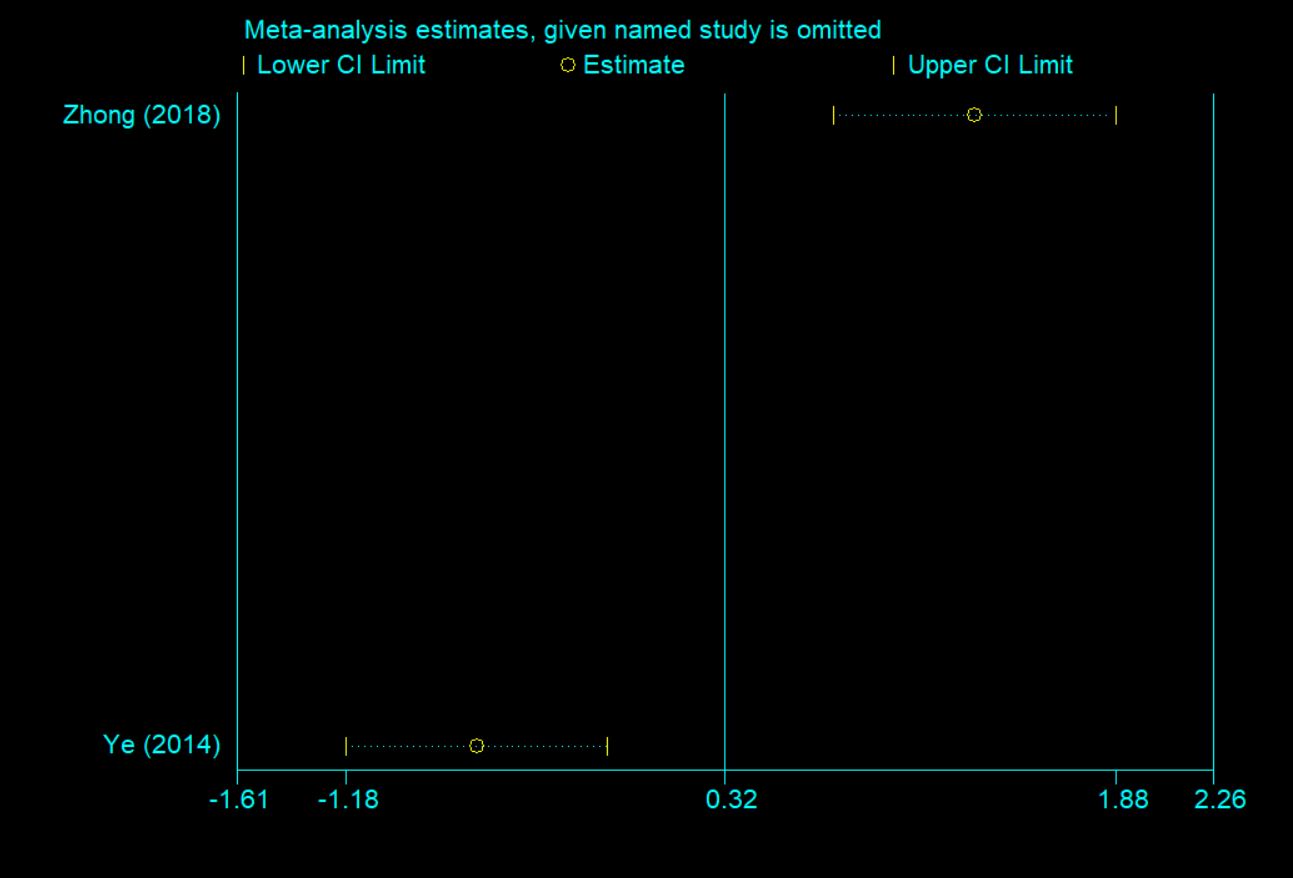


Supplementary FIGURE S9.4 The sensitivity analysis plot of E_2_ (AST).


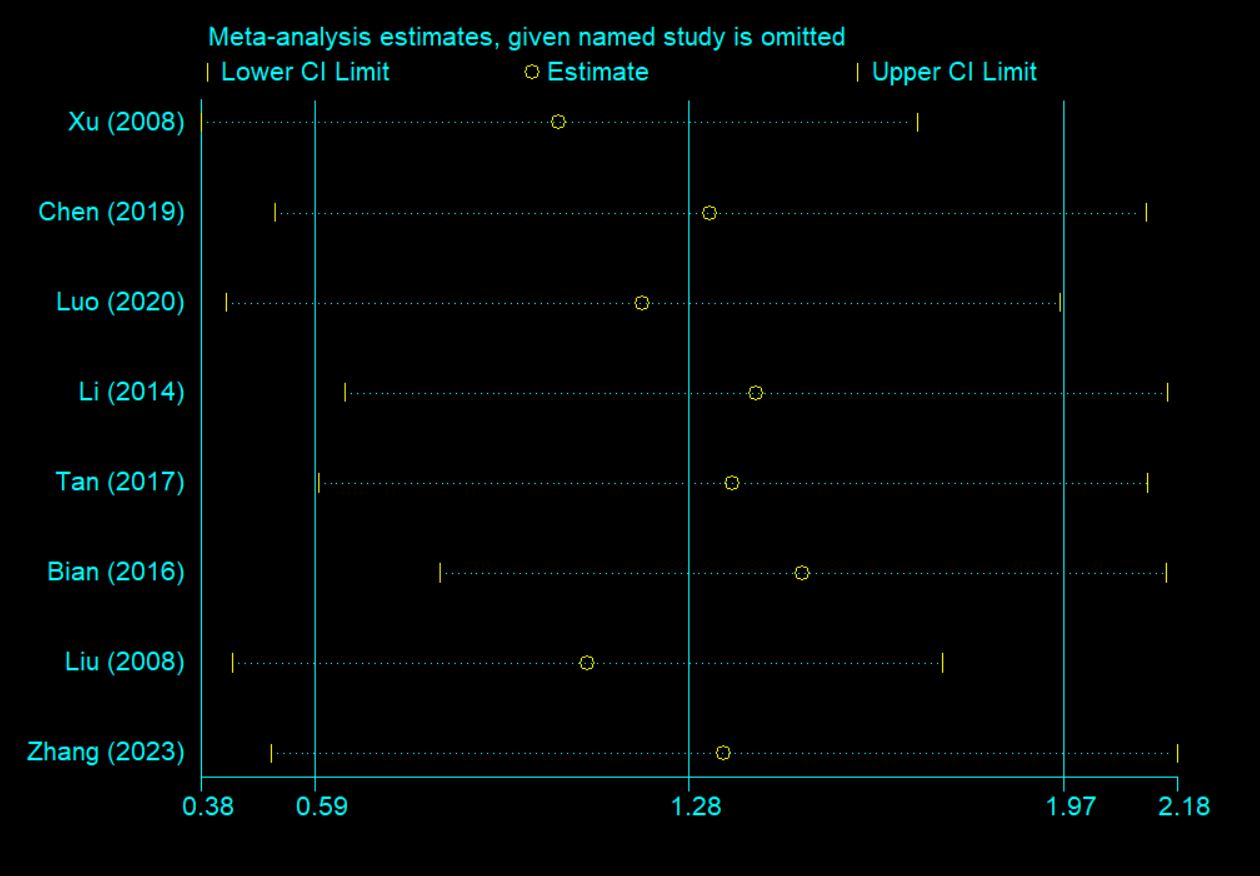


Supplementary FIGURE S9.5 The sensitivity analysis plot of E_2_ (CIAA).


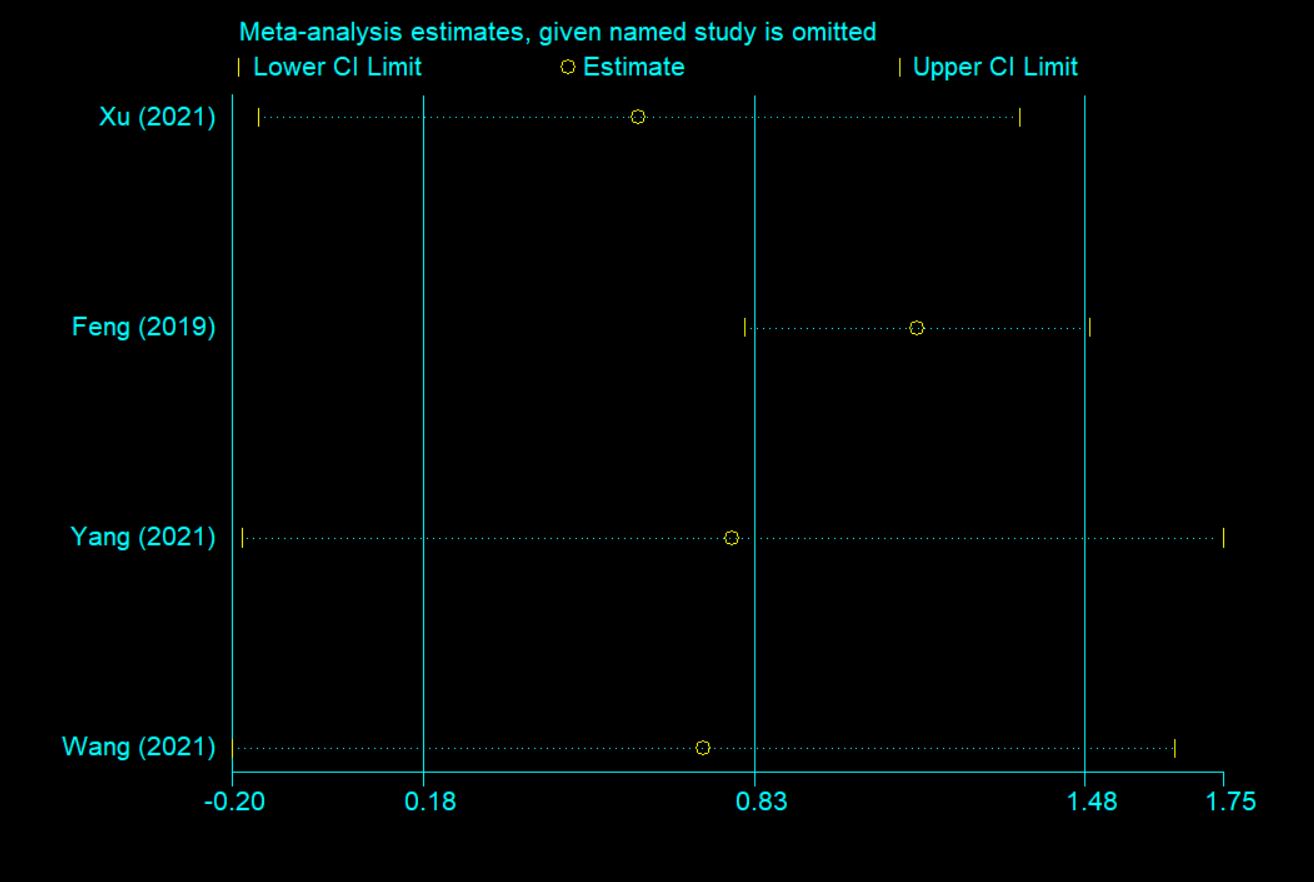


Supplementary FIGURE S9.6 The sensitivity analysis plot of E_2_ (Moxi).


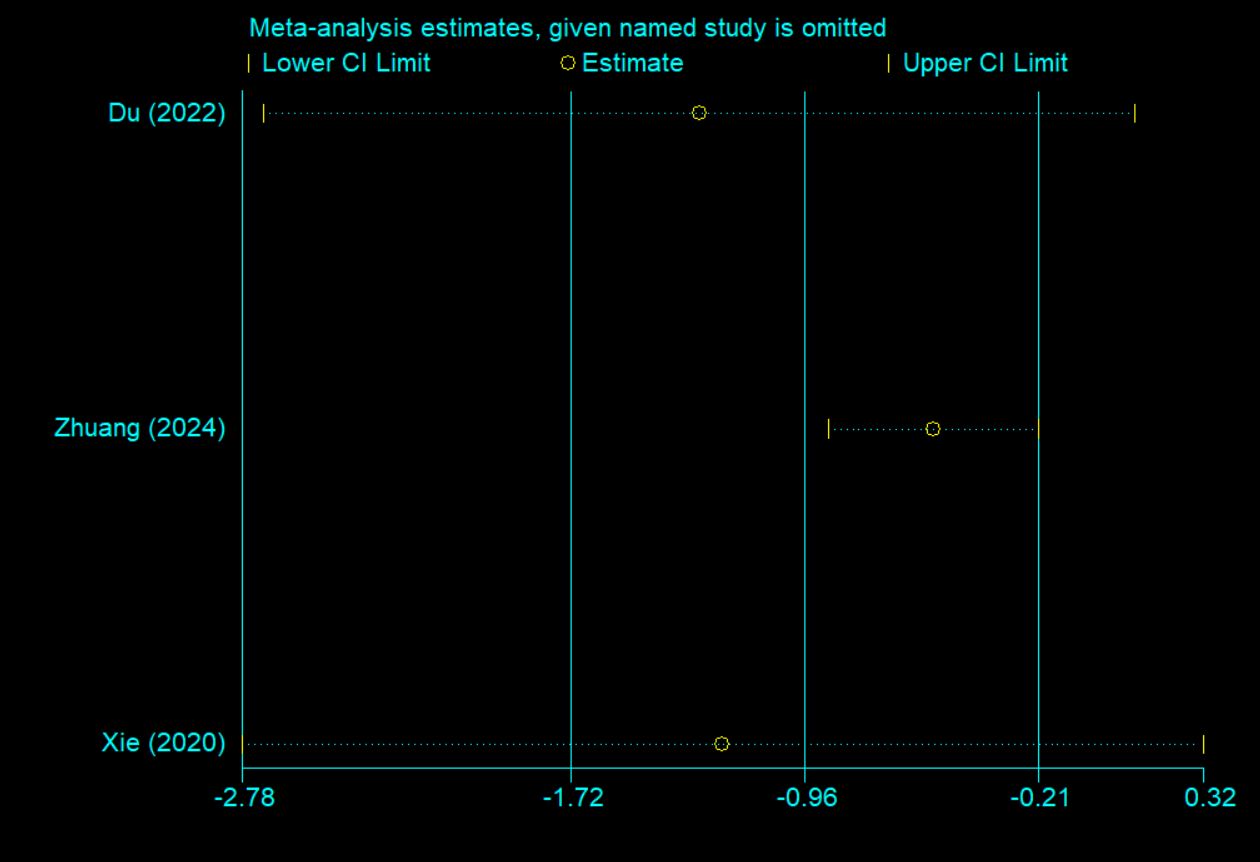


Supplementary FIGURE S10.1 The sensitivity analysis plot of Kupperman (Acu + Moxi).


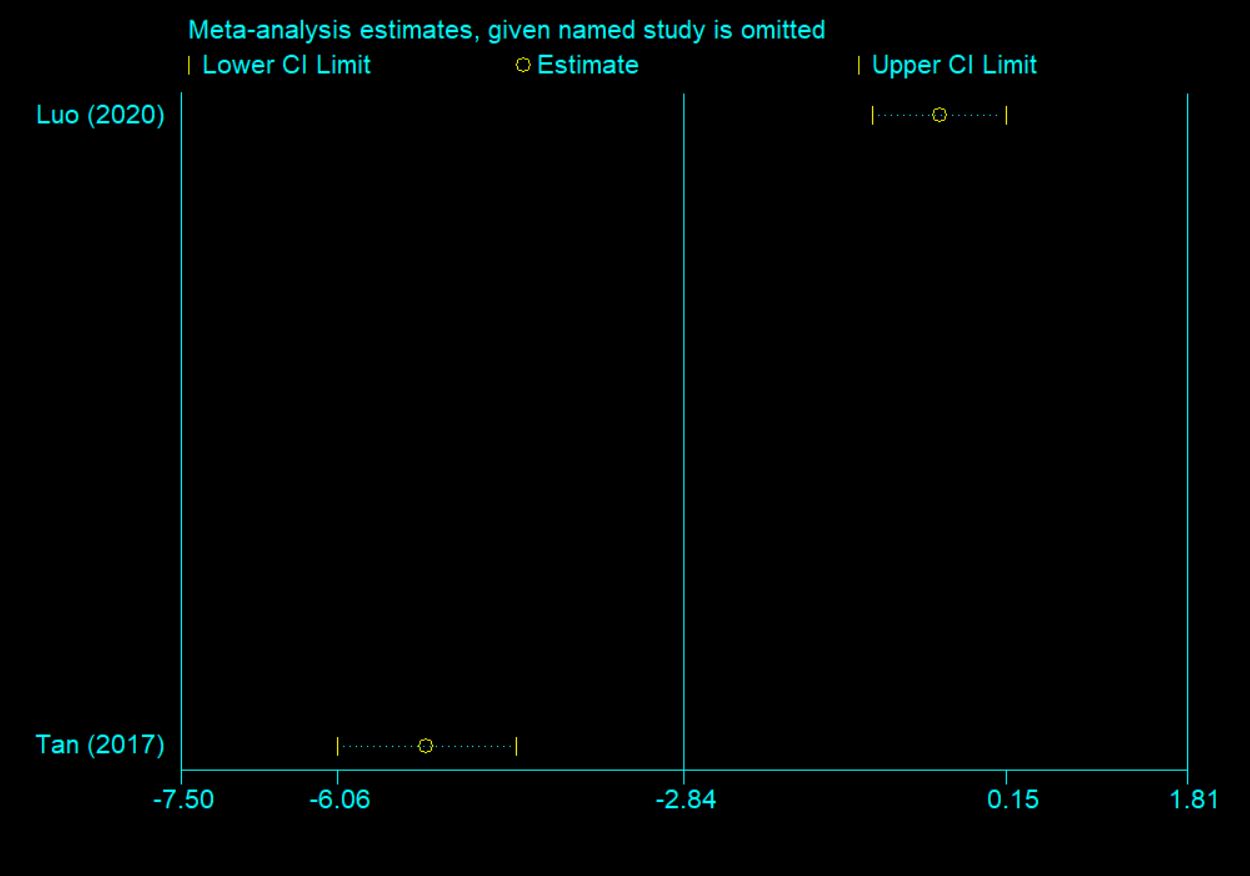


Supplementary FIGURE S10.2 The sensitivity analysis plot of Kupperman (CIAA).
